# Systematic review and meta-analysis of the seroprevalence of hepatitis E virus in the general population across non-endemic countries

**DOI:** 10.1371/journal.pone.0216826

**Published:** 2019-06-07

**Authors:** Barbara Wilhelm, Lisa Waddell, Judy Greig, Ian Young

**Affiliations:** 1 Big Sky Health Analytics, Vermilion, Alberta, Canada; 2 National Microbiology Laboratory at Guelph, Public Health Agency of Canada, Guelph, Ontario, Canada; 3 School of Occupational and Public Health, Ryerson University, Toronto, Ontario, Canada; Centers for Disease Control and Prevention, UNITED STATES

## Abstract

**Background:**

Hepatitis E virus (HEV) has commonly been associated with large waterborne outbreaks of human jaundice in endemic areas but it has been increasingly recognised as a cause of sporadic human cases of jaundice in non-endemic areas, in individuals with no history of travel. Zoonotic exposure is widely hypothesized to be an important potential transmission route in these sporadic human cases. Serosurveys conducted to determine the frequency of HEV human exposure report wide ranges in prevalence across studies and locations. Our study objective was to compute meta-analysis summary estimates of human seroprevalence of HEV IgG within countries considered HEV non-endemic, where possible, and to determine whether this varied significantly across these countries, as well as investigating the role of potential HEV seroprevalence predictors such as population age structure.

**Materials and methods:**

A broad literature search was conducted in six electronic databases. Citations were appraised, and relevant data extracted using forms designed and pre-tested *a priori*. Meta-analysis and meta-regression were conducted in R, with HEV IgG seroprevalence in blood donors or the general population being the outcome of interest, and country, assay, population age and sex structure, and chronological time investigated as predictors of the outcome.

**Results:**

From 4163 unique citations initially captured, data were extracted from 135 studies investigating HEV serology in blood donors or the general population, of 31 countries among those categorised as ‘very high human development’ by the United Nations. Country of sampling and assay employed were consistently significant predictors of HEV IgG seroprevalence with chronological time being a non-significant predictor in the dataset of captured studies.

**Conclusions:**

While country of sampling and assay employed were significant predictors of HEV seroprevalence, comparison of HEV seroprevalence across non-endemic countries is hampered by the lack of a gold standard assay and uncertainty regarding residual bias across studies, as well as regional differences within some countries.

## Introduction

Hepatitis E virus (HEV) is a member of the family *Hepeviridae*; within the genus *Orthohepevirus*, *species Orthohepevirus A* includes eight recognised genotypes of HEV [1]. Genotypes 1 and 2 HEV have only been detected in humans, and these infections frequently result in outbreaks of jaundice, in areas traditionally considered endemic, which are resource-poor, where HEV is spread by the fecal-oral route often via contaminated water [2,3]. However, since the mid-1990s, sporadic cases of locally acquired hepatitis E have been reported in non-endemic regions (i.e. in industrialised countries with public health infrastructure not permissive of waterborne disease outbreaks), and in individuals with no history of travel to endemic regions [4]. Since the first report of HEV detection in swine in 1990, pigs and subsequently other animals have been hypothesized as exposure sources for these sporadic locally acquired Hepatitis E cases, caused by infections with HEV genotype 3 and to a lesser extent genotype 4 [1,5]. Exposure for source attribution of human cases can be challenging to determine serologically since although there are multiple genotypes of HEV, only 1 serotype is recognised [1].

In some non-endemic regions, for example within the European Union, concern has been expressed that the incidence of clinical cases of locally acquired Hepatitis E, (i.e. involving genotypes 3 or 4) is increasing, although the extent to which this increase may reflect increased physician awareness and enhanced testing is unclear [6]. Additionally, most individuals infected with HEV in non-endemic countries seroconvert asymptomatically and these asymptomatic viremic individuals could potentially contaminate the blood supply if they donate blood while viremic [7]. Consequently, serosurveys of blood donors and other defined groups have been conducted to study the frequency of asymptomatic HEV infection in non-endemic regions. Some significant variation in HEV IgG seroprevalence has been reported, from 4.7% of blood donors in Scotland [8] to 52.5% of blood donors in southwestern France [9]. Interpretation of the variations in prevalence reported by these individual studies is challenging, since HEV IgG seroprevalence has been associated with increasing age [10– 13], sex [10,14], and assay employed [15–17]. However, currently the potential association between these predictors and HEV seroprevalence *across* studies is unclear.

Investigation of human HEV IgG seroprevalence in non-endemic countries requires definition of relevant sampling locations. The human development index of the United Nations’ Human Development Programme offers a transparent method of categorising countries based on a combination of metrics including life expectancy, expected years of schooling, mean years of schooling, and gross national income (GNI) per capita [18]. Inclusion in the ‘very high development’ category of this index could be deemed evidence that a country’s public health capacity and infrastructure would preclude large waterborne outbreaks of viral hepatitis. Therefore, HEV seroprevalence studies conducted in countries categorised ‘very high development’ would likely reflect exposure to HEV genotypes 3 or 4, unless participants have a history of travel to endemic areas.

Investigation of HEV seroprevalence across countries and studies also requires appropriate methodology. Systematic review methodology has been used for decades to describe and synthesize human medical research, and guidelines for execution and reporting of systematic reviews have been developed [19, 20]. Following systematic review, meta-analysis allows the pooling of results to compute a summary estimate of effect; if data regarding potential predictors have been captured, meta-regression, i.e. the regression of one or more study-level covariates on the dependent variable (in this case, HEV seroprevalence) allows computation of measures of association between predictors and outcome, across studies [21]. Therefore, systematic review and meta-analysis are potentially useful methods for partitioning true variation in HEV seroprevalence across countries relative to sampling error and investigating the magnitude of variation explained by various predictors [21]. For this reason, systematic review and meta-analysis are increasingly important methods for informing policy decisions pertaining to healthcare issues [22].

The objective of this systematic review was to compute meta-analysis summary estimates of human seroprevalence of HEV IgG within non-endemic countries in the general population, where possible, and to determine whether this varied significantly across these countries. Sub-objectives were to estimate the proportion of variance of HEV seroprevalence explained at the study and country level, as well as investigating the potential association between demographic parameters such as population age structure, and HEV seroprevalence across non-endemic countries.

## Methods

### Scope

This systematic review was conducted following a protocol prepared *a priori* and reported according to the Preferred Reporting Items for Systematic Reviews and Meta-Analyses (PRISMA) guidelines [20]. The study protocol is available in S1 File and a checklist of the PRISMA assessment for this systematic review is presented in S7 File.

Inclusion/exclusion criteria for the SR were defined using the CoCoPop acronym [22]:

*Condition* (outcome of interest): Measurement of HEV IgG antibodies was deemed relevant. Total HEV antibodies, IgM antibodies, and detection of HEV RNA (e.g. using reverse transcriptase polymerase chain reaction) were deemed not relevant outcomes for this review. Included studies were required to employ a defined, reproducible assay.

*Context*: Environmental factors can have a substantial impact on the prevalence or incidence of a condition. Some demographic descriptors have been associated with odds of HEV seropositivity including socio-economic status [23], occupation [24], recreational activities [9], dietary preferences [11], and rural, relative to urban, residence [25]. Therefore, these parameters were captured when reported by investigators. A complete list of contextual parameters captured is listed in the data extraction tool available in S2 File.

*Population*: Inclusion: people living in countries categorized as ‘very high’ in the human development index by the United Nations [18]. A list of the countries categorised as ‘very high’ is presented in S3 File. Given the consistent association reported within individual studies, between subject age and odds of seropositivity, descriptors of population age and sex structure were captured when these parameters were reported by investigators. Travellers, recent immigrants, and traveling members of armed forces were excluded from the systematic review due to the difficulty in establishing the country of origin of infection in these groups. Liver patients were categorically excluded from this systematic review, as were groups consisting of only Hepatitis E patients. Data from defined sub-groups of the general population, potentially differing from the general population regarding their probability of HEV exposure, (e.g. farmers or targeted patient groups such as hemophiliacs), were captured in the overall systematic review, but will be analysed and reported separately from the general population.

Inclusion criteria for meta-analysis were: studies employing an assay that was reported in five or more studies (assays employed in four or less studies were categorised as ‘other’ in analysis), sampling groups identified as representative of the general population, or blood donors. Due to the association reported between subject age and probability of HEV IgG sero-positivity, studies only sampling children or pregnant women were identified in the systematic review, but not included in meta-analysis.

Investigation of the potential association of HEV IgG seroprevalence and risk factors for human HEV exposure beyond possible population structure confounders and spatial-temporal relationships, will be reported in a companion paper.

### Search strategy

A broad electronic search was conducted in the following electronic bibliographic databases on 29 November 2016: Embase (biomedical and pharmacological database produced by Elsevier), PubMed, Scopus, Global Health (Public Health and Tropical Medicine (PHTM) database, previously produced by the Bureau of Hygiene and Tropical Diseases (BHTD), and the human health and diseases information extracted from CAB ABSTRACTS), Epub Ahead of Print, In-Process & Other Non-Indexed Citations in Ovid MEDLINE(R) Daily and Ovid MEDLINE(R). A pretested search algorithm was developed to capture all relevant research:

("hepatitis E virus" OR "Hepatitis E virus" OR HEV) AND (blood OR serum OR serology OR sero-prevalence OR plasma OR “plasma products”)

Specific algorithms used in each database are presented in the study protocol S1 File. The following literature reviews were hand-searched for additional citations potentially missed by the electronic search: [2, 4, 26–32].

The following websites were also hand-searched for additional citations, for the past three years: The International Liver Congress of the European Association for the Study of the Liver; European Congress of Clinical Microbiology and Infectious Diseases; and IDWeek. A search was conducted using the Google search engine, employing the same terms as the electronic bibliographic search, 25 May 2017.

### Systematic review management

**Fig 1:**
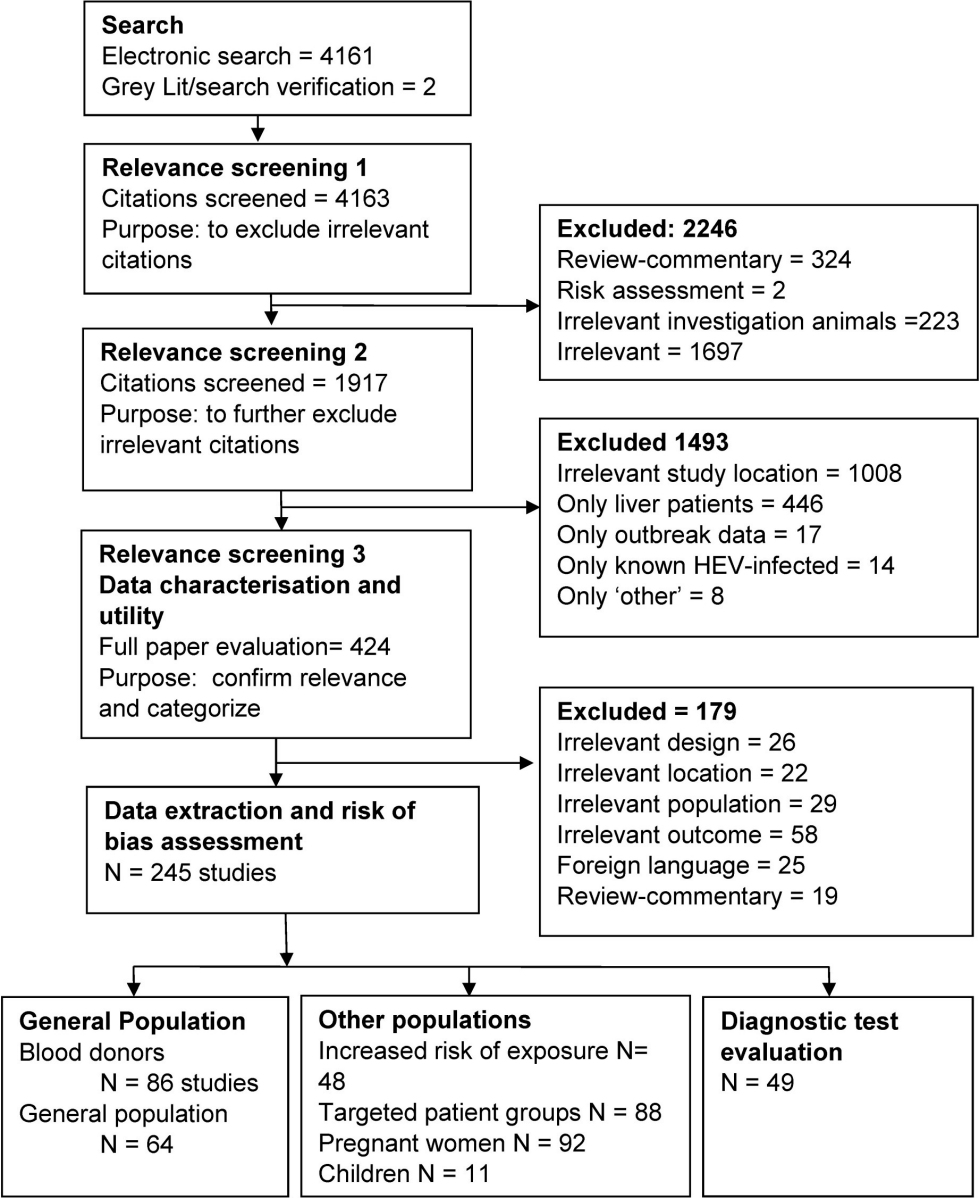
Flow of citations and articles through the systematic review process.

Captured citations were saved to Refworks (Proquest LLC, Ann Arbor, Michigan, USA), de-duplicated, then uploaded to the Distiller electronic platform (Evidence Partners, Ottawa, ON, Canada). Reviewing forms were drafted for first and second level relevance screening, risk of bias assessment, and data extraction. These were pre-tested *a priori* on a selected subset of citations and full papers. Reviewing at each level (Fig 1) was initiated after agreement of 0.80 or greater was achieved across reviewers assessed by Cohen’s kappa statistic. Risk of bias of individual studies was assessed using the following criteria: considering whether all intended study outcomes relevant to this review were reported, the presence of potential confounders was considered and adjusted for, as well as an overall assessment of the risk of bias for the population(s) studied.

Reviewing was conducted independently by two epidemiologists, at each level of the systematic review. First level relevance screening was performed on the abstract, to assess relevance and exclude completely irrelevant citations. Second level relevance screening confirmed relevance and categorised studies by population investigated. Data extraction included capture of parameters such as location and year of sampling, assay(s) employed, storage and handling of samples, and demographics of the population sampled. All forms used in this systematic review are presented in S2 File.

### Analysis

Descriptive statistics of the dataset were computed in Excel (Microsoft 365, Microsoft Corp).

Meta-analysis was conducted in the R Studio platform (R Studio, 250 Northern Ave, Boston, MA, USA) using the R software environment [33]. Random effects meta-analysis was selected based on the assumption of true variation of HEV seroprevalence across studies. Heterogeneity was quantified by calculation of Higgins’ *I*^2^ [34] and T^2^, an estimate of τ^2^, which represents the true variance in prevalence across studies [21]. The restricted maximum likelihood (REML) method was selected to compute T^2^ due to its minimal bias and efficiency [35].

Heterogeneity of effect estimates within a dataset was categorised as ‘low’ if *I*^2^ ≤ 60% and T (the computed estimate of τ, or the true standard deviation) was less than the meta-analysis summary estimate of prevalence. For datasets not categorized with ‘low’ heterogeneity, the median and range of individual study prevalence estimates are presented in lieu of meta-analysis summary estimates and 95% confidence intervals. Meta-analysis model assumptions of normality were investigated using the Shapiro-Wilk normality test [36], and a visual examination of the quantile-quantile Normal plots [37].

Potential publication bias, a form of small study bias, was assessed by Egger’s regression test [38], the rank correlation test [39], and the trim-and-fill method of Duval and Tweedie [40]. These tests were applied to all datasets meeting the ‘lenient’ criteria outlined by Ionnidis and Trikalinos (2007) [41]: minimum of five surveys within the dataset, and Higgins’ *I*^2^ ≤ 50%.

Variables previously reported as predictors of HEV IgG seroprevalence in the literature within studies were investigated as a source of variability across studies using random effects meta-regression, including age, sex, ethnicity, geographic location, and population sub-groups. Sex and age were investigated as continuous variables. The sex structure of a study population was captured as the proportion of the study population reported as male, and the age structure was captured by reported median age of the population. While both blood donor and general population sampling frames were *a priori* deemed appropriate for investigating HEV IgG seroprevalence within a country, similarity in seroprevalence between these groups was investigated during analysis, prior to consideration of pooling data from these two groups.

“Assay” was investigated as a categorical predictor of HEV IgG seroprevalence, if the assay in question was reported in five or more studies. Given reports that the prevalence of both HEV exposure and incidence of clinical Hepatitis E cases is increasing over time [4], chronological time was also investigated as a potential predictor of HEV IgG seroprevalence across studies. While outbreaks of waterborne jaundice caused by HEV have been reported since the 1980s, the beginning of the investigation of HEV in non-endemic countries coincided with the detection of HEV in swine [5]. For this reason, the association between time and HEV seroprevalence in non-endemic countries was investigated by computing sampling year starting with the year 1990 designated as year 0. Since collinearity between chronological time and assay could not be quantitatively assessed in meta-regression, an adjusted year of sampling was computed for each study group captured, and then centred, to mitigate the potential association between calendar chronological time, and development and uptake of assays with improved diagnostic sensitivity and specificity [37]. For those studies not reporting the year of sample collection, we imputed the year of collection as year of publication -2, and then computed an adjusted and centred year of sampling as described above. For each included study, therefore, an ‘adjusted’ year of sampling was computed as a potential predictor.

Preliminary investigation of a subset of countries having 10 or more surveys in blood donors or the general population was conducted using the R package ‘metafor’ [42] for meta-analysis and meta-regression. The hierarchical structure of the dataset (surveys within studies within countries) was investigated in multilevel modeling in ‘metafor’. As well, the association between predictors and HEV seroprevalence previously reported within studies, was studied across studies captured in our dataset. We considered for inclusion in the multivariable model all variables for which *P* < 0.20 in univariable analysis. In general, the objective of meta-regression is to quantitatively investigate the association between predictors and heterogeneity, or true variation in seroprevalence, across studies. However, the objective of this preliminary meta-regression was more specifically to explore whether the presence or absence of reporting of potential predictors within included studies should be considered in defining additional inclusion-exclusion criteria for meta-analysis datasets. For example, if median age of the population sampled (frequently reported as a significant predictor of HEV seroprevalence within studies) were demonstrated to be significant predictor of HEV seroprevalence across studies, and some studies failed to report this parameter, the failure to report of population age structure by an individual study could be considered as a potential criterion for exclusion from the meta-analysis datasets.

Subsequent analysis of the datasets for HEV IgG seroprevalence for all included countries were analysed using the R package ‘meta’ [43]. The Freeman-Tukey double arcsine transformation was applied to datasets, given the relatively low HEV seroprevalence reported in many studies [22, 44]. For those datasets in which the majority of studies reported prevalence > 10% and/or the Shapiro Wilk statistic was significant (*P* < 0.05), the logit transformation was applied [45]. Random effects meta-regression in the R package ‘meta’, using the ‘metareg’ command, was employed to assess the association between a predictor variable and HEV IgG seroprevalence across studies [46]. All variables for which *P* < 0.20 in univariable analysis were considered for inclusion in the multivariable model.

## Results

The search captured 4161 unique citations; two additional citations were captured by search verification [47, 48]. At first level relevance screening, 2246 citations were excluded, most frequently since they were not relevant to the systematic review question (Fig 1). Second level relevance screening excluded an additional 1493 citations, most frequently due to sampling in study locations outside of the review scope, or sampling ONLY liver patients, or presenting ONLY data investigating ‘other’ topics such as potential sources of human HEV exposure (‘Only other’, Fig 1). At the third level screening, another 179 studies were deemed irrelevant, for reasons including irrelevant location of sampling, or irrelevant study design such as outbreak data or case reports, or measured outcomes other than HEV IgG (for example, HEV RNA or total immunoglobulin, or investigations in which the specific kit or assay method used was unclear). Diagnostic test evaluations were reported in 49 studies, describing assays of HEV IgG (n = 49), and IgM (n = 24). Data on HEV IgG seroprevalence was extracted from 245 unique studies. A list of their major characteristics, including country of sampling, population sampled, and assays employed, is presented in the supplementary material S1 Table. Data were captured from 31 countries categorised as ‘very high’ human development. Of the broad population types captured by our review, targeted patient groups (e.g. HIV patients) were the most frequently sampled (n = 135 surveys), followed by blood donors (n = 110 surveys). France (n = 34), Germany (29), and Italy (31) were the countries in which most sampling was conducted (Table 1). Within this broad group of 245 HEV IgG seroprevalence studies, 135 studies sampled either blood donors or the general population. Some studies reported multiple unique surveys of different populations; in total 183 unique surveys, consisting of blood donors (n = 108 surveys) or the general population (n = 75) were captured and are summarized in the remainder of this systematic review.

**Table 1:** Characteristics of 245 studies reporting Hepatitis E virus IgG seroprevalence, conducted in 31 countries, sampling blood donors, the general population, and other selected groups.

	Number (proportion) of studies within country						
Country	Blood donors	General Population	Increased risk of exposure^1^	Targeted patient groups^2^	Pregnant women	Children^3^	Total number studies
**Argentina**	2 (20%)	2 (20%)		5 (50%)		1 (10%)	10
**Australia**	2 (66%)		1 (33%)				3
**Austria**	1 (50%)	1 (50%)					2
**Canada**	1 (33%)		1 (33%)	1 (33%)			3
**Croatia**	1 (20%)	1 (20%)	1 (20%)	2 (40%)			5
**Czech Republic**		2 (100%)					2
**Denmark**	2 (50%)		2 (50%)				4
**Estonia**			1 (100%)				1
**France**	8 (24%)	3 (9%)	5 (15%)	15 (44%)	1 (3%)	2 (6%)	34
**Germany**	9 (31%)	5 (17%)	4 (14%)	8 (28%)	1 (3%)	2 (7%)	29
**Greece**	3 (25%)	1 (8%)	2 (16%)	6 (50%)		1	13
**Hong Kong**		3 (100%)					3
**Iceland**		1 (50%)	1 (50%)				2
**Ireland**	1 (50%)	1 (50%)					2
**Israel**	2 (40%)		1 (20%)	1 (20%)	1 (20%)		5
**Italy**	10 (32%)	4 (13%)	9 (29%)	8 (26%)			31
**Japan**	7 (26%)	8 (30%)	3 (11%)	7 (26%)		2 (7%)	27
**South Korea**	1 (20%)	3 (60%)	1 (20%)				5
**Netherlands**	5 (29%)	5 (29%)	1 (6%)	6 (35%)			17
**New Zealand**	2 (66%)					1 (33%)	3
**Norway**	1 (33%)	1 (33%)	1 (33%)				3
**Poland**	1 (16%)	1 (16%)	2 (33%)	2 (33%)			6
**Portugal**	2 (16%)	3 (25%)	3 (25%)		1 (8%)	3 (25%)	12
**Qatar**	1 (100%)						1
**Russia**	1 (100%)						1
**Saudi Arabia**	3 (43%)	3 (43%)		1 (14%)			7
**Spain**	3 (10%)	5 (17%)	1 (3%)	15 (52%)	5 (17%)		29
**Sweden**	1 (16%)	2 (32%)	1 (16%)	2 (32%)			6
**Switzerland**	3 (43%)		2 (29%)	1 (14%)	1 (14%)		7
**UAE**					2 (100%)		2
**UK**	4 (33%)	2 (16%)	1 (8%)	5 (42%)			12
**USA**	10 (34%)	6 (21%)	5 (17%)	7 (24%)	1 (3%)		29
TOTAL studies*	87	62	48	92	13	10	

* Total is >245 because some studies report outcomes for more than one sample population or country.

^1^ Increased risk of exposure = for example, occupational exposure, recreational exposure, intravenous drug use.

^2^ Targeted patient group = for example, hemodialysis patients, transplant patients, hospital ward and outpatients.

^3^ Children = participants < 18 years old

While a broad range of assays were employed across 135 studies sampling blood donors or the general population, several commercial kits predominated, including the kit from Abbott laboratories (n = 22 surveys), especially in earlier work, and the Wantai (n = 40), Mikrogen (n = 13), and MP Biomedical, formerly Genelabs (n = 25) kits in more recent investigations (Table 2). Some assays were more frequently employed in investigations from specific countries (Table 2).

**Table 2:** Year of publication and assays employed by HEV seroprevalence surveys of blood donors or the general population.

	Year of publication					Assay employed									
	Pre -2000	2000- 2004	2005- 2009	2010- 2014	2015 on	Abbott	Bio-elisa	Dia. Pro	DSI	EIA-gen	In-house	Mikrogen	MP Biomedical	Wantai	Other
**Country**															
**Argentina**	1	0	0	4	1	1	0	2	0	0	0	0	0	3	0
**Australia**	1	0	0	1	0	0	0	0	0	0	0	0	1	1	0
**Austria**	0	0	0	1	1	0	0	0	0	0	0	0	0	2	0
**Canada**	0	0	0	0	1	0	0	0	0	0	0	0	0	1	0
**Croatia**	0	0	0	0	2	0	0	1	0	0		0	0	0	1
**Czech Republic**	0	0	0	1	1	0	0	1	0	0	0	0	0	0	1
**Denmark**	0	0	2	0	2	0	0	0	0	0	3	0	0	1	0
**France**	1	0	2	5	3	0	0	0	0	0	1	0	4	6	0
**Germany**	2	0	0	19	0	2	0	0	0	0	4	9	4	1	1
**Greece**	3	0	0	1	1	3	0	0	0	1	0	0	1	0	0
**Hong Kong**	1	1	0	0	0	0	0	0	0	0	0	0	2	0	0
**Iceland**	0	0	0	0	2	0	0	1	0	0	0	0	0	1	0
**Ireland**	0	0	0	0	2	0	0	0	0	0	0	0	0	2	0
**Israel**	1	0	0	0	1	0	0	0	1	0	1	0	0	0	0
**Italy**	5	0	1	3	4	4	0	3	1	1	0	0	0	3	1
**Japan**	0	4	10	2	1	0	0	0	0	0	15	0	0	0	2
**Korea**	0	1	1	3	0	0	0	0	0	0	0	0	3	2	0
**Netherlands**	2	0	2	7	1	1	0	0	0	0	0	0	4	7	0
**NZ**	0	0	1	0	0	0	0	0	0	0	0	0	0	1	0
**Norway**	0	2	0	0	1	2	0	0	0	0	0	0	0	1	0
**Poland**	0	0	0	0	2	0	0	0	0	0	0	0	0	1	1
**Portugal**	0	0	0	2	3	0	0	0	0	0	0	3	0	1	1
**Qatar**	0	0	0	0	1	0	0	0	0	0	0	0	0	1	0
**Russia**	0	1	0	0	0	0	0	0	0	0	1	0	0	0	0
**Saudi Arabia**	5	1		2	0	3	1	0	0	0	3	0	0	0	1
Spain	3	1	1	2	3	3	2	0	0	0	0	0	1	1	1
**Sweden**	1	0	1	0	5	2	0	0	1	1	0	1	0	0	2
**Switzerland**	1	0	0	3	1	1	0	1	0	0	0	0	1	2	0
**UK**	0	0	0	3	6	0	0	0	0	0	0	0	1	8	0
**USA**	6	6	2	5	3	0	0	0	0	0	13	0	3	2	4
**TOTAL surveys**	33	16	23	64	47	22	3	9	3	3	41	13	25	40	24

A preliminary univariable meta-regression study of predictors previously reported significant within studies was conducted to identify potential predictors across studies, within each country from which approximately 10 or more surveys of blood donors or the general population were conducted. Details of these datasets for France, Germany, Italy, the Netherlands, and the USA are presented in S4 File. Assay employed was frequently a significant predictor of seroprevalence across surveys (five of seven datasets/countries investigated). Year of sampling was a significant predictor of HEV IgG seroprevalence in several countries (France, Italy, and Netherlands). The relatively small number of surveys in these datasets (9–22 surveys per country) did not provide sufficient power for investigation of multivariable meta-regression. Median age of the population, proportion of males in the population, and membership in the general population, relative to blood donors, were all non-significant predictors of HEV IgG seroprevalence *across* studies, within the selected country datasets.

Multilevel models featuring surveys clustered within studies were fitted; however, profile plots of the variance components consistently suggested mis-specification of the model for each of the countries investigated [49]. Consequently, meta-regression models were fitted with robust standard errors, in an alternative attempt to estimate unbiased standard errors given the hierarchical nature of the dataset; these models failed to converge. No further efforts were made to adjust for the hierarchical nature of the dataset.

Therefore, meta-analysis was performed on the pooled dataset of surveys sampling either blood donors or the general population for each country included in the systematic review, if two or more surveys were obtained from the country of interest. If the dataset were categorised as ‘high’ heterogeneity, meta-analysis was performed on that country’s dataset, stratified by assay where possible. To further investigate ‘country’ as a potential predictor of seroprevalence, meta-analysis was performed on the combined dataset of blood donor and general population surveys captured by this systematic review, stratified by assay. Country and chronological time were investigated by meta-regression within each assay-stratified dataset as outlined above.

**Fig 2:**
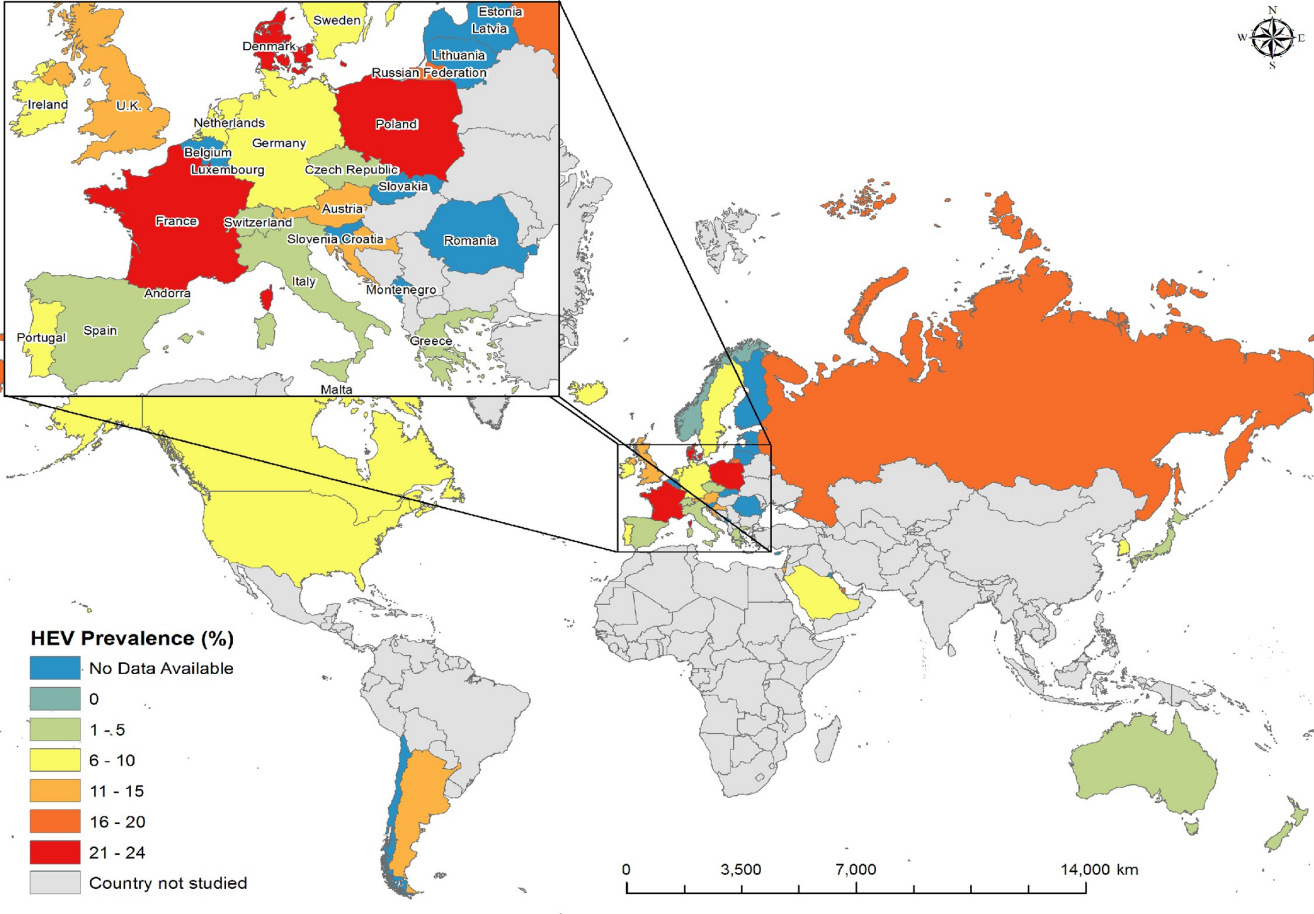
HEV IgG seroprevalence across countries categorised as ‘very high’ human development.

Summary estimates of HEV IgG seroprevalence in blood donors or the general population, presented by country, stratified where possible by assay employed, are presented in Table 3. A list of the included studies with their individual datasets as well as full citations for each study are presented in S5 and S6 Files. Regional differences were noted within some countries. For example, analysis of the entire UK dataset yielded a summary estimate with high heterogeneity (*I*^2^ = 95.6%, T^2^ = 0.007). However, stratified findings, separating England (HEV seroprevalence = 13.8%, 95% CI (12.8%; 14.9%) and Scotland (HEV seroprevalence = 4.9%, 95% CI (4.0%; 5.9%), substantially reduced the heterogeneity originally observed (Table 3). Similarly, in Italy, HEV IgG seroprevalence, measured by the same assay, varied significantly at the 95% confidence level between studies conducted within the same country [50, 51]. A chloropleth map summarizing the data presented in Table 3 is presented in Fig 2.

**Table 3:** Summary of Hepatitis E virus (HEV) IgG seroprevalence surveys in blood donors or the general population, presented by country, stratified where possible by assay employed.

Country	Assay^a^	Number surveys (Number sampled)	Hetero-geneity Cat-egorized^b^	HEV Seroprevalence Meta-analysis summary estimate^c^ (95% Confidence Intervals)	I^2^ (Tau^2^)	HEV Seroprevalence Median (Minimum, Maximum)	Citations
**Argentina**	All	6 (2764)	High	N/A	N/A	11.9% [1.8%, 14.8%]	[64–66]
	Wantai	3 (2264)	Low	15.2% [7.7%, 24.3%]	0 (0)	14.8% [14.2%, 16.7%]	[65]
	Dia.Pro	2 (1369)	Low	5.8% [4.2%, 7.8%]	45.1% (0.0008)	7.4% [4.4%, 9.4%]	[64–65]
**Australia**	All	2 (3516)	High	N/A	N/A	[0.04%, 6.0%]	[67–68]
**Austria**	Wantai	2 (2200)	High	N/A	N/A	[12.5%; 15.4%)	[69–70]
**Canada**	Wantai	1 (4102)	N/A	5.9%	N/A	NA	[10]
**Croatia**	All	2 (1073)	High	N/A	N/A	[2.7%, 20.3%]	[71–72]
**Czech Republic**	All	2 (1949)	High	N/A	N/A	[2.5%, 5.7%]	[73–74]
**Denmark^e^**	All	4 (1631)	N/A	N/A	N/A	[10.7%, 32.9%]	[58] [75]
**France^d^**	All	11(20100)	High	N/A	N/A	23.6% [3.2%, 52.5%]	[9] [13] [25] [57] [76–82]
	Wantai	6 (16838)	High	N/A	N/A	6.6% [0.224, 0.525]	[9][57][80–82]
	MP Biomedical	4 (2984)	High	N/A	N/A	17.9% [16.6%, 26.1%]	[25][76–78]
**Germany^d^^,^^e^**	All	21 (11105)	N/A	N/A	N/A	8.08% [2.0%, 16.8%]	[15] [16] [83–93]
	Mikrogen	9 (6708)	High	N/A	N/A	9.7% [6.0%, 16.8%]	[15] [85–88] [91–92]
	MP Biomedical	4 (1383)	Low	3.6% [2.1%, 5.3%]	54.4% (0.001)		[15] [16] [83] [89]
**Greece**	All	5 (4797)	High	N/A	N/A	2.2% [0.2%, 9.4%]	[94–97]
	Abbott	3 (3332)	High	N/A	N/A	0.5% [0.2%, 2.2%]	[94] [96]
**Hong Kong**	MP Biomedical	2 (1289)	Low	17.9% [15.4%, 20.5%]	24.6% (0.0002)	[16.1%, 18.8%]	[98] [99]
**Iceland^e^**	All	2 (195)	N/A	N/A	N/A	[6.2%, 9.2%]	[54]
**Ireland**	Wantai	2 (1274)	Low	6.2% [3.8%; 9.1%]	57.0% (0.001)	[5.3%, 8.1%]	[100–101]
**Israel**	All	2 (777)	Low	10.5% [8.4%; 12.8%]	0 (0)	[10.6%, 14.6%]	[102–103]
**Italy** **^e^**	All	13(7008)	High	N/A	N/A	2.6% [1.1%, 4.9%]	[50] [51] [55] [104–110]
	Wantai	3 (585)	High	N/A	N/A	4.9% [1.4%, 48.9%]	[104–105] [109]
	Abbott	4 (4827)	High	N/A	N/A	1.3% [0.7%, 2.6%]	[50] [51] [55]
**Japan**	All	17 (54721)	High	N/A	N/A	4.3% [0.5%, 15.8%]	[12] [111–122]
**Korea^e^**	All	5 (3201)	High	N/A	N/A	9.4% [4.7%, 39.9%]	[123–126]
	MP Biomedical	3 (604)	Low	13.5% [10.5%, 16.8%]	14.8% (0.0003)	5.8% [4.7%, 11.9%]	[123–125]
	Wantai	2 (2597)	High	N/A	N/A	[5.9%, 23.1%]	[125–126]
**Netherlands^d^^,^^e^**	All	14 (15209)	N/A	N/A	N/A	6.5% [1.8%, 38.3%]	[17][127–135]
	MP Biomedical	5 (6782)	High	N/A	N/A	1.9% [1.8%, 7.2%]	[17][127–128] [133][135]
	Wantai	7 (7152)	High	N/A	N/A	19.8% [4.3%, 38.3%]	[129][130–132]
**Norway**	All	3 (1603)	High	N/A	N/A	0.5% [0%, 13.5%]	[136–137]
**New Zealand**	Wantai	1 (265)	N/A	4.2% [2.2%, 7.5%]	N/A	NA	[138]
**Poland**	All	2 (2269)	High	N/A	N/A	Range (3.4%, 43.7%)	[14] [139]
**Portugal**	All	5 (3049)	High	N/A	N/A	9.0% [2.1%, 19.9%]	[140–144]
	Mikrogen	3 (2691)	High	N/A	N/A	9.0% [2.6%, 19.9%]	[141] [143–144]
**Qatar**	Wantai	1 (5042)	N/A	20.2% [19.1%, 21.4%]	N/A	N/A	[145]
**Russia**	In-house	1 (185)	N/A	17.8%	N/A	N/A	[146]
**Saudi Arabia**	All	8 (12855)	High	N/A	N/A	10.1% [0.1%, 18.7%]	[60] [147–151]
	Abbott	3 (1818)	High	N/A	N/A	10.8% [8.4%, 14.9%]	[147–148]
**Spain^e^**	All	8 (7367)	-N/A	N/A	N/A	4.2% [2.2%, 20.0%]	[152–158]
	Abbott	3 (1418)	Low	3.3% [2.4%, 3.8%]	0.0% (0.0001)	4.1% [2.9%, 4.2%]	[153] [155–156]
	Bioelisa	2 (1480)	High	N/A	N/A	[3.5%, 7.5%]	[152] [157]
**Sweden^e^**	All	7 (2957)	N/A	N/A	N/A	9.3% [4.8%, 19.0%]	[53] [159–160]
	Abbott	2 (457)	Low	6.6% [3.1%, 11.1]	56.7% (0.002)	[5.2%, 11.3%]	[159–160]
**Switzerland^e^**	All	5 (5353)	N/A	N/A	N/A	4.9% [4.2%, 21.8%]	[161–163]
	Wantai	2 (4159)	Low	20.1% [18.1%, 22.2%]	38.0% (0.0002)	[19.5%. 21.8%]	[161] [163]
**UK^d^^,^^e^**	All	9 (7031)	N/A	N/A	N/A	13.0% [3.6%, 15.8%]	[8] [164–168]
	Wantai	8 (6531)	High	N/A	N/A	13.2% [4.9%, 15.8%]	[8] [164–168]
	Wantai_ England	6 (4444)	Low	13.8% [12.8%, 14.9%]	9.3% (<0.001)	13.7% [11.8%, 16.2%]	[164–168]
	Wantai_ Scotland	2 (2087)	Low	4.9% [4.0%, 5.9%]	0	[4.7%, 5.7%]	[8]
**USA^e^**	All	22 (57822)	N/A	N/A	N/A	10.1% [0, 21.8%]	[11][24][84] [146][169–178]
	MP Biomedical	3 (9537)	High	N/A	N/A	7.3% [1.4%, 10.5%]	[169][174] [178]
	Wantai	2 (1939)	High	N/A	N/A	[13.5%, 24.8%]	[176]

^a‘^All’ = the analysed dataset included all of the studies sampling blood donors or the general population within a given country, regardless of assay employed.

^b^Heterogeneity categorized as ‘Low’ was presented if I^2^ < = 60, and (MA summary estimate > Tau).

^c^Meta-analysis summary estimate and 95% confidence intervals are presented if I^2^ is <60%, considered low. Median and range of individual study estimates are presented with measures of heterogeneity, if heterogeneity categorized as ‘high’, and dataset consisted of 3 or more studies, or only a range of individual study estimates if only two studies are in the dataset.

^d^Meta-regression performed on the dataset including ‘All’ assays used to study populations in this country yielded a significant (P < 0.05) association between ‘Assay’ and HEV IgG sero-prevalence.

^e^This dataset included multiple sampling of one or more study populations, using different assays. Datasets which included the application of multiple assays to the same study population were not included in meta-analysis but stratified so that assay-specific meta-analysis could be performed, where possible.

The potential for significant variation in HEV IgG seroprevalence across countries is also supported by the meta-regression performed on datasets stratified by assay, presented in Table 4. For example, computing a meta-analysis summary estimate of HEV seroprevalence across countries, including only studies employing the Abbott kit, ‘country’ is a significant (*P* <0.0001) predictor of heterogeneity, explaining 87.21% of dataset heterogeneity. Similarly, country is a significant (*P* < 0.05) predictor of HEV IgG seroprevalence across the MP Biomedical- and Wantai-stratified datasets.

**Table 4:** Meta-regression of HEV IgG seroprevalence stratified by assay and examining chronological time and country in univariate analyses.

Assay	Predictors (Number of populations surveyed, Number of countries)	Predictor significance (R^2^)	Estimate 95% CI	I^2	Tau^2
**Abbott**					
	Null (22,9)	N/A	Med = 1.9% (0, 14.9%%)	96.0% [94.9%; 96.9%]	0.0087
Model 1	Country	<0.0001 (87.21%)		73.38%	0.0010 (SE = 0.0006)
Model 2	Year	0.9611 (0)		96.59%	0.0091 (SE = 0.0033)
**Mikrogen**					
	Null (13, 4)	N/A	Med = 9.9% (2.6%, 19.9%)	94.4% [92.1%; 95.9%]	0.105
Model 1	Country	0.0648 (25.0%)		94.41	0.079 (SE = 0.0040)
Model 2	Year	0.126 8.32%		95.27%	0.0096 (SE = 0.0042)
**MP Biomedical**					
	Null (25, 9)	N/A	Med = 5.5% (0.4%, 19.2%)	97.6% [97.1%; 98.0%]	0.015
Model 1	Country	0.0006 (48.35%)		95.54%	0.0879
Model 2	Year	0.8281 (0)			
**Wantai**					
	Null (40, 20)	N/A	Med = 14.6% (1.4%, 4.9%)	99.1% [99.0%; 99.2%]	0.027
Model 1	Country	0.025 (25.54%)		98.58%	0.021 (SE = 0.0061)
Model 2	Year	0.5909 0		99.27%	0.028 (SE = 0.0060)

Abbott assay dataset: included studies = [50–51] [55] [66] [90] [93–94] [96] [134] [136] [147–148] [153] [155–156] [162]

Mikrogen = [15] [16] [53] [85–88] [91–92] [141] [143–144] [158]

MPBiomedical = [15][25] [67] [76–78] [83][89] [97–99] [123–125] [127–128] [133] [135] [161] [165] [169][174][178]

Wantai = [8–10] [13–14] [16] [54] [57] [65] [68] [69–70] [75] [80–82] [100–101] [104–105][109] [125–126] [129–132] [137–138][140] [145] [158][161][163–168] [176]

In contrast, adjusted year of sampling, after stratifying for assay employed, is consistently a non-significant (*P* > 0.05) predictor of HEV IgG seroprevalence across studies, in assay-stratified datasets, for each of the commercial kits’ datasets for which meta-regression was performed (Abbott, Mikrogen, MP Biomedical and Wantai).

None of the datasets organized by country or by assay, met our systematic review criteria for investigation of potential publication bias, due either to the high heterogeneity or the small number of surveys across each dataset.

Risk of bias assessment for each included study is presented in the supplementary material S2 Table; overall findings for risk of bias criteria across studies sampling blood donors or the general population are presented in Table 5. The most frequently employed sampling strategy was convenience (n = 101/135 studies); the direction of the potential bias this sampling strategy could cause is difficult to predict. The predominance of convenience sampling could reflect the affiliations of many authors with universities, or university hospitals, which frequently furnished the sampling frame for the study. While failure to report participant ethnicity was the most frequently identified potential source of bias, non-reporting of the age structure of individual study populations was also frequently noted. However, overall, most of the studies included in meta-analysis (n = 92/135) were categorised as ‘low’ risk of bias.

**Table 5:** Risk of bias across studies of blood donors or the general population.

Parameter	Number of studies
**Representativeness justified**	
** Yes**	24 (18%)
** No**	111 (82%)
**Samples handled and processed appropriately**	
** Yes**	42 (31%)
** No**	4 (3%)
** Not reported**	89 (66%)
**Sampling strategy for individuals**	
** Whole registry**	12 (9%)
** Random**	3 (2%)
** Reported random**	13 (9%)
** Systematic**	8 (6%)
** Convenience**	101 (74%)
**Risk of bias from selective reporting**	
** Low**	118 (87%)
** Unclear**	13 (10%)
** High**	4 (3%)
**Risk of bias from confounding**	
** Low**	22 (16%)
** Unclear**	104 (78%)
** High**	9 (6%)
**Overall risk of bias**	
** Low**	92 (68%)
** Unclear**	29 (21%)
** High**	14 (11%)

## Discussion

Since HEV infection in swine was described in 1990, human HEV infections in non-endemic areas have been a topic of increasing interest in medicine, public health, and within national blood supply services [6,7]. However, despite several decades of scientific study, inconsistent findings have been reported in field surveys of human HEV IgG seroprevalence, differing hypotheses have been proposed regarding exposure sources in asymptomatic human HEV infections in non-endemic countries, and varying assessments of the potential public health impact of HEV across non-endemic countries have been expressed [1,7].

In every non-endemic country from which research was captured in this systematic review, a measurable proportion of the general population or blood donors sampled had serological evidence of HEV exposure, presumably acquired during asymptomatic infection. Researchers have correctly asserted that the variation observed across some surveys and countries is difficult to interpret given the range of assays, which vary in performance [52], as well as variation in HEV seroprevalence across population subgroups [7] and the associations between assay, country, and chronological time.

In the dataset of HEV surveys captured, median age, and proportion of the population which was male, were not associated with HEV seroprevalence across studies. However, descriptors of age structure in the population were missing from 30–50% of studies within individual country datasets, with sex being reported even less consistently relative to age. Given the high proportion of missing data, analysis of the subset of studies reporting age of the population sampled could have generated biased estimates of association between these predictors and HEV IgG seroprevalence across studies, the direction and magnitude of which are unknown. Overall, the non-significant association observed across studies between age structure and seroprevalence, is evidence of investigators employing sampling frames with similar age structures.

In contrast, ‘assay’ was a significant predictor of HEV seroprevalence within and across studies. This predictor also had the potential to behave as an effect modifier across countries, since some assays were more frequently employed in some countries (Tables 2 and 3). Assay performance was investigated in several studies, frequently employing multiple assays on the same study population [15, 16, 53–55]. Variations in assay performance reported by these studies suggest that some individual participants may have been mis-classified by some assays. Currently, HEV IgG assay diagnostic sensitivity and specificity may be estimated from a comparison with subjects’ concurrent RT-PCR status, or categorisation in two or more serological assays [3]. Alternatively, in situations such as the study of HEV assay performance in blood donors, in which there may be neither a gold standard, nor a comparator test with known characteristics within the study population, latent class models allow simultaneous estimation of sensitivity and specificity of multiple assays [56]. These models may be fitted without making assumptions about the true disease status of each subject and can permit relaxation of the assumption of independence among assays which is necessary in other approaches [56].

The HEV IgG seroprevalence reported in individual studies, as well as computed HEV seroprevalence summary meta-analysis estimates, are a function of true prevalence, as well as the diagnostic sensitivity (the proportion of truly HEV ‘positive’ samples correctly categorised by the test in question) and specificity the proportion of truly HEV ‘negative’ samples correctly categorised by the test in question) of the assay employed. Therefore, quantitative estimation of HEV assay performance would allow future computation and comparison of true prevalence across studies. Currently, in the absence of estimates of assay performance, true HEV seroprevalence is difficult to estimate.

However, controlling for assay by stratifying the dataset, ‘country’ is a significant predictor of HEV IgG seroprevalence within meta-regression models for the Abbott, MP Biomedical, and Wantai datasets. The non-significant *P* value for the predictor ‘country’ in the Mikrogen dataset could reflect inadequate power to detect an association in this relatively small dataset. As well, the non-significant association could reflect the specific countries represented; none of the countries with more extreme summary estimates of seroprevalence, such as Australia, or France, are represented in this dataset. Our observation that HEV IgG seroprevalence varies significantly across non-endemic countries, after adjusting for assay, is consistent with previous reports, and suggests that both known and unknown predictors represented by ‘country’, may be associated with human HEV seroprevalence [7, 57]. As a predictor, ‘country’ could act as a surrogate measure for several risk factors that may impact IgG HEV seroprevalence including the national proportion of specific ethnic groups, national eating habits, or the prevalence of HEV infection in domestic pigs [1]. Within some countries there appear to be significant regional differences in human HEV IgG seroprevalence, such as in France, where the southwestern region has been reported to have significantly higher HEV IgG seroprevalence [13], or the UK, where Scotland has been reported to have lower seroprevalence relative to England (Table 3). These regional differences are characterized as unexplained heterogeneity in meta-analysis summary estimates of seroprevalence when viewed from the national perspective, until more detailed local studies investigate reasons for these apparent differences [3]. The regional differences in prevalence may reflect the varying burden of HEV contamination in food sources, potentially coinciding with dietary preferences such as consumption of raw or undercooked liver; water contamination; or even the frequency or magnitude of local ‘outbreaks’ of asymptomatic infection.

While the incidence of clinical cases of Hepatitis E has been reported to be on the rise in some European countries, in our dataset, chronological time was not a predictor of HEV IgG seroprevalence. This is consistent with other studies using several different lines of inquiry, both genomic and serological [58, 59]. Ideally, primary research investigating the effect of chronological time on HEV seroprevalence could employ the same HEV assay, with known performance characteristics, on samples collected at various points in time. However, the logistics of such a study, including adequate sample handling and storage, are challenging, and perhaps as a result this sort of study is rarely conducted. That said, it is noteworthy that one such study, conducted in Denmark, reported decreasing HEV seroprevalence over time [58]. The increased incidence of clinical cases of Hepatitis E in some European countries, therefore, may reflect increased awareness by clinicians resulting in more frequent diagnosis, or a true increase in incidence [6].

Even after characterisation of some potential regional differences, seroprevalence models may still contain residual unexplained model heterogeneity across studies employing the same assays (Tables 3 and 4), which could reflect variation in demographics (e.g. socio-economic risk factors), individual laboratory protocols (e.g. treatment of ‘grey zone’ findings), or other factors.

Most studies included in meta-analysis did not justify the representativeness of their study populations relative to a larger target population; however, in individual studies this was usually not a stated objective. Therefore, it is difficult to estimate the degree to which the overall dataset of included studies is similar with respective national populations. This may be further exacerbated by the frequent employment of convenience sampling strategies (75%, 100/134 studies); the potential magnitude and direction of bias introduced with this type of sampling strategy is impossible to predict. However, the ‘general population’ and blood donor study groups, did not differ significantly with regards to HEV IgG seroprevalence and in this systematic review were considered equally potentially representative of the target population. The influence of ethnicity on overall population HEV IgG seroprevalence has been previously reported [60]. For this reason, failure to consider or describe individuals’ ethnicity as a potential predictor, was deemed to be a potential source of bias. Recent research suggests that in North America, ethnic minorities may be under-represented in the blood donor pool [61]. Similarly, sex was also inconsistently associated with seropositivity within individual studies; we considered the failure to report the proportion of each sex within an individual study, not suggestive of overall risk of bias. In contrast, studies not reporting any description of the age structure of the study population were deemed to have unclear risk of bias as the relationship between age and seroprevalence has within-studies been well established [10–13].

The specific conditions for sample handling, particularly holding temperature, were frequently not reported (31% (41/134 studies, reporting), and could have an important impact on the study results particularly for studies that test stored samples. Findings from studies reporting samples held at an inadequate temperature were deemed more likely to under-estimate HEV IgG seroprevalence.

While considering the captured seroprevalence studies to have a hierarchical data structure (surveys within studies within countries) seemed plausible, we were unable to fit multilevel models in this dataset. Consequently, we were not able to partition the proportion of variance occurring at each level of the dataset (survey-study-country), and their relative contributions remain unclear.

A systematic review investigating a specific intervention typically would include a formal evaluation of the degree of confidence in its findings, or underpinning weight of evidence; the Cochrane Collaboration’s Grading of Recommendation, Assessment, Development and Evaluation (GRADE) approach is widely used to assess the weight of evidence underpinning meta-analysis summary estimates of effect [62]. Currently no comparable tool exists for the assessment of systematic reviews of prevalence. However, the heterogeneity and its multiple sources across studies within our dataset suggest that future research may significantly affect the findings of this review.

This is especially true of countries with relatively greater magnitude of seroprevalence estimates, as well as high heterogeneity. Investigation of HEV seroprevalence within defined regions of these countries can help to identify areas of ‘hyper-endemnicity’ such as those reported in southwest France [9], or the Abruzzi region of Italy [3]. This research is helpful in defining natural experiments (i.e. locations within country having higher and lower seroprevalence). Further investigation of the distribution of potential risk factors across these regions could improve our understanding of important sources of human HEV exposure, and this work would be of value to local public health agencies.

It is important to note that comparisons which might be made across countries, given the data presented in Tables 3 and 4, would necessarily be made on the assumption of no residual bias (selection, information, or confounding) existing across studies.

Since the alternative to changes in smaller-scale, country-level serosurveys, i.e. the implementation of a large-scale international serosurvey, is unlikely to occur due to logistical and resource challenges, several changes in the potential conduct of smaller, within-country studies, are proposed. Future research targeting more comprehensive regional sampling could help to define local regions of higher mean HEV IgG seroprevalence. Sampling of potential human exposure sources within these regions for evidence of HEV exposure, or HEV detection, including food, domestic animals, and wildlife [63] could be used to generate more specific hypotheses regarding potential human HEV exposure sources and how these may contribute to variations in seroprevalence.

In the absence of changes in the conduct of within-country serosurveys, or estimation diagnostic test performance, especially diagnostic (as opposed to analytical) sensitivity, the comparison of seroprevalence across countries remains challenging. Currently comparison of HEV seroprevalence across countries requires synthesis of studies employing the same assay, with the inherent assumption that no residual bias exists across studies.

## Conclusions

Every non-endemic country from which data was captured reported that a variable proportion of the general population has serological evidence of HEV exposure, with country and assay employed being significant predictors of HEV seroprevalence. HEV IgG seroprevalence varied significantly in study groups representing the general population across some non-endemic countries, when controlling for assay. However, in the absence of data regarding assay diagnostic sensitivity and specificity, true HEV seroprevalence cannot be precisely computed. In datasets across non-endemic countries, stratified by assay, residual heterogeneity beyond that explained by ‘country’ could reflect variations in population attributes, agricultural practices, or specific laboratory protocols. Further research synthesis comparing the diagnostic sensitivity and specificity of the commonly employed assays would allow for meaningful comparisons of HEV seroprevalence across countries.

## Supporting information

S1 Table Major characteristics of included studies. (DOCX)

S2 Table Risk of bias assessment of included studies. (DOCX)

S1 File Study protocol. (DOCX)

S2 File Systematic review tools. (DOCX)

S3 File Relevant study locations. (DOCX)

S4 File Preliminary analysis of selected country datasets in ‘metafor’. (DOCX)

S5 File Meta-analysis datasets. (DOCX)

S6 File List of included studies. (DOCX)

S7 File PRISMA checklist. (DOCX)
