# Supplementary material for: Systematic review and meta-analysis of the seroprevalence of hepatitis E virus in the general population across non-endemic countries
Source: PLoS One. 2019 Jun 7;14(6):e0216826. doi: 10.1371/journal.pone.0216826 (PMC6555507; doi:10.1371/journal.pone.0216826)
Supplement: S1 Table — (DOCX) [file pone.0216826.s001.docx]

# S1 Table. Major characteristics of included studies investigating HEV seroprevalence in blood donors or the general population

| **Country** | **Citation** | **Populations^1^** | **Outcome measured** | **Assay^2^** |
| --- | --- | --- | --- | --- |
| Argentina |  |  |  |  |
|  | Debes et al., 2016 | GP, TPG | IgG | Dia.Pro |
|  | Munne et al., 2014 | BD, GP, TPG | IgG, IgM | Wantai, Dia.Pro |
|  | Rey et al., 1997 | BD, TPG, Ch | IgG | Abbott |
| Australia |  |  |  |  |
|  | Moaven et al., 1995 | BD, TPG, IR | IgG, IgM | MP Biomedical (Genelabs) |
|  | Shrestha et al., 2014 | BD | IgG, IgM | Wantai |
|  |  |  |  |  |
| Austria | Fischer et al., 2015 | BD | IgG, IgM | Wantai |
|  | Lagler et al., 2014 | AF | IgG | Fortress Diagnostics |
| Canada |  |  |  |  |
|  | Fearon et al., 2017 | BD | IgG, IgM | Wantai |
| Croatia |  |  |  |  |
|  | Vilibic-Cavlek et al., 2016 | GP, TPG, IR | IgG, IgM | Euroimmun |
|  | Miletic Lovric et al., 2014 | BD | IgG, IgM | Dia.Pro |
| Czech Republic |  |  |  |  |
|  | Strakova et al., 2014 | GP | IgG | Dia.Pro |
|  | Nemecek et al., 2015 | GP | IgG | Dia.Pro |
| Denmark |  |  |  |  |
|  | Christensen et al., 2008 | BD, IR | IgG | In-house assay |
|  | Holm et al., 2015 | BD | IgG | Wantai, In-house |
| France |  |  |  |  |
|  | Boutrouille et al., 2007 | BD | IgG | MP Biomedical (Genelabs) |
|  | Carpentier et al., 2012 | GP, IR | IgG, IgM | MP Biomedical (Genelabs) |
|  | Chaussade et al., 2013 | GP, IR | IgG, IgM | MP Biomedical (Genelabs) |
|  | Coursaget al., 1994 | GP | IgG | In-house |
|  | Gallian et al., 2014 | BD | IgG, IgM | Wantai |
|  | Izopet et al., 2015 | BD, TPG | IgG | Wantai |
|  | Mansuy et al., 2016 | BD | IgG, IgM | Wantai |
|  | Mansuy et al., 2015 | BD | IgG, IgM | Wantai |
|  | Mansuy et al., 2011 | BD, Ch | IgG | Wantai |
|  | Mansuy et al., 2008 | BD | IgG | MP Biomedical (Genelabs) |
|  | Dalton et al., 2010 | BD, Ch | IgG | Wantai |
| Germany |  |  |  |  |
|  | Baylis et al., 2010 | BD | IgG, IgM | MP Biomedical (Genelabs) |
|  | Dawson et al., 1992 | BD, TPG, Ch | IgG | In-house assay |
|  | Dremsek et al., 2012 | BD, IR | IgG | Mikrogen recomLine |
|  | Faber et al., 2012 | GP | IgG | Mikrogen recomLine |
|  | Juhl et al., 2014 | BD | IgG, IgM | Mikrogen recomWell |
|  | Krumbholz et al., 2014b | GP, IR | IgG, IgM | Mikrogen recomWell |
|  | Krumbholz et al., 2012 | BD, IR | IgG, IgM | Mikrogen recomLine |
|  | Pischke et al., 2014 | TPG, BD, GP | IgG | MP Biomedical (Genelabs) |
|  | Pischke et al., 2011 | BD, GP | IgG | Abbott Laboratories |
|  | Reinheimer et al., 2012 | BD, TPG | IgG | Mikrogen recomWell |
|  | Vollmer et al., 2012 | BD | IgG, IgM | Mikrogen recomLine |
|  | Wenzel et al., 2013 | GP | IgG, IgM | MP Biomedical (Genelabs); Wantai, Mikrogen recomLine |
|  | Wichmann et al., 2008 | GP | IgM | Mikrogen |
|  | Pischke et al., 2010b | BD, GP | IgG | Abbott Laboratories |
|  | Pischke et al., 2013 | GP, TPG | IgG | MP Biomedical (Genelabs) |
| Greece |  |  |  |  |
|  | Dalekos et al., 1998 | BD, IR, TPG, Ch | IgG, IgM | Abbott Laboratories |
|  | Pittaras et al., 2014 | BD | IgG | EIAgen (Adaltis) |
|  | Psichogiou et al., 1996 | GP, TPG, IR | IgG, IgM | Abbott Laboratories |
|  | Zervou et al., 2015 | BD, TPG | IgG, IgM | MP Biomedical (Genelabs) |
| Hong Kong |  |  |  |  |
|  | Lok et al., 1992 | GP, TPG | IgG, IgM | MP Biomedical (Genelabs) |
|  | Wong et al., 2004 | GP | IgG | MP Biomedical (Genelabs) |
| Iceland |  |  |  |  |
|  | Löve et al., 2018 | GP, IR | IgG | Dia.Pro, Wantai |
| Ireland |  |  |  |  |
|  | O'Riordan et al., 2016 | BD | IgG, IgM | Wantai |
|  | Hickey et al., 2016 | GP | IgG | Wantai |
| Israel |  |  |  |  |
|  | Mor et al., 2015 | BD, TPG | IgG | DS EIA |
|  | Keretny et al., 1996 | BD, NB | IgG | In-house assay |
| Italy |  |  |  |  |
|  | Caruso et al., 2016 | GP, IR | IgG | Wantai |
|  | Gessoni et al., 1996 | GP, TPG, IR | IgG | Abbott Laboratories |
|  | Lucarelli et al., 2016 | BD | IgG, IgM | Wantai |
|  | Masia et al., 2009 | BD, IR | IgG | DiaPro |
|  | Pavia et al., 1998 | BD | IgG | Abbott Laboratories, International Immunodiagnostics |
|  | Puttini et al., 2015 | BD, TPG | IgG | EIAgen (Adaltis) |
|  | Rapicetta et al., 2013 | BD, IR | IgG | DS EIA, DSI |
|  | Ricco et al., 2016 | BD, TPG | IgG, IgM | Wantai, Dia.Pro |
|  | Scotto et al., 2014 | BD, GP, IR, TPG | IgG, IgM | Dia.Pro |
|  | Scotto et al., 2012 | BD | IgG, IgM | Dia.Pro |
|  | Zanetti et al., 1994 | BD, GP, IR, TPG | IgG, IgM | Abbott Laboratories |
|  | Gessoni et al., 1998 | BD, IR, TPG | IgG | Abbott Laboratories |
| Japan |  |  |  |  |
|  | Fukuda et al., 2007 | BD | IgG, IgM | In-house assay |
|  | Fukuda et al., 2004 | BD | IgG, IgM | In-house assay |
|  | Gotanda et al., 2007 | BD | IgG, IgM | In-house assay |
|  | Li et al., 2000 | GP | IgG, IgM | In-house assay |
|  | Mitsui et al., 2005 | GP, TPG | IgG, IgM | In-house |
|  | Sakata et al., 2008 | BD | IgG, IgM | In-house |
|  | Takahashi et al., 2010 | GP | IgG, IgM | In-house |
|  | Takahashi et al., 2005 | BD, TPG | IgG, IgM | In-house |
|  | Takeda et al., 2010 | BD | IgG | In-house |
|  | Tanaka et al., 2005 | GP | IgG |  |
|  | Tei et al., 2004 | GP, IR | IgG | Viragent |
|  | Toyoda et al., 2008b | GP, IR | IgG | In-house |
|  | Ding et al., 2003 | GP, TPG, IR, Ch | IgG, IgM | In-house |
|  | Fukae et al., 2016 | GP, TPG | IgG, IgM | Institute of Immunology |
| South Korea |  |  |  |  |
|  | Ahn et al., 2005 | GP | IgG | MP Biomedical (Genelabs) |
|  | Choi et al., 2003 | GP | IgG | MP Biomedical (Genelabs) |
|  | Park et al., 2012 | GP | IgG | Wantai, Genelabs |
|  | Yoon et al., 2014 | GP | IgG | Wantai |
| Netherlands |  |  |  |  |
|  | Bouwknegt et al., 2008 | GP, IR | IgG, IgM | MP Biomedical (Genelabs)  Abbott Laboratories |
|  | Herremans et al., 2007a | GP, TPG | IgG, IgM | MP Biomedical (Genelabs) |
|  | Herremans et al., 2007b | BD, TPG | IgG, IgM | MP Biomedical (Genelabs) |
|  | Hogema et al., 2014 | BD | IgG | Wantai |
|  | Slot et al., 2013 | BD | IgG, IgM | Wantai |
|  | Verhoef et al., 2012 | GP | IgG | MP Biomedical (Genelabs) |
|  | Zaaijer et al., 1995 | BD, TPG | IgG | Abbott Laboratories |
|  | Zaaijer et al., 1992 | BD, TPG | IgG | MP Biomedical (Genelabs) |
|  | Sadik et al., 2016 | GP | IgG, IgM | Wantai |
|  | Van den Berg et al., 2014 | GP | IgG, IgM | Wantai |
| New Zealand |  |  |  |  |
|  | Dalton et al., 2007 | BD | IgG | Wantai |
| Norway |  |  |  |  |
|  | Andenaes et al., 2000 | GP, IR | IgG | Abbott Laboratories |
|  | Lange et al., 2017 | BD, AF, IR | IgG, IgM | Wantai |
| Poland |  |  |  |  |
|  | Bukowska et al., 2016 | GP, TPG, IR | IgG, IgM | Euroimmun |
|  | Sulkowska et al., 2016 | BD | IgG, IgM | Wantai |
| Portugal |  |  |  |  |
|  | Mesquita et al., 2014 | GP, IR | IgG | Wantai |
|  | Sargento et al., 2016 | BD, TPG, IR | IgG, IgM | Euroimmun |
|  | Pereira et al., 2016 | GP | IgG, IgM | Mikrogen recomWell |
|  | Sargento et al., 2014 | BD, TPG | IgG, IgM | Annagene |
|  | Teixeira et al., 2017 | GP, IR | IgG | Mikrogen recomWell |
| Qatar |  |  |  |  |
|  | Nasrallah et al., 2016 | BD | IgG, IgM | Wantai |
| Russia |  |  |  |  |
|  | Obriadina et al., 2002 | BD | IgG | In-house |
| Saudi Arabia |  |  |  |  |
|  | Abdelaal et al., 1998 | BD | IgG | In house assay |
|  | Arif et al., 1994 | GP | IgG | Abbott Laboratories |
|  | Ayoola et al., 2002 | GP, TPG | IgG, IgM | Abbott Laboratories, AMRAD |
|  | Johargy et al., 2013 | BD | IgG, IgM | Bioelisa |
|  | Paul et al., 1994 | BD | IgG, IgM | In-house |
|  | Elsheikh et al., 2012 | GP | IgG, IgM | Diagnotic Automation |
| Spain |  |  |  |  |
|  | Buti et al., 2006 | GP | IgG | Bioelisa |
|  | Buti et al., 1995 | GP, TPG | IgG, IgM | Abbott Laboratories |
|  | Fogeda et al., 2012 | GP | IgG, IgM | Diagnostic Bioprobes |
|  | Mateos et al., 1999 | BD, TPG, Ch | IgG, IgM | Abbott Laboratories |
|  | Medrano et al., 1995 | BD, TPG | IgG | Abbott Laboratories |
|  | Meng et al., 2002 | BD, IR | IgG | In-house assays |
|  | Riveiro-Barciela et al., 2014 | GP, TPG | IgG | Bioelisa |
|  | Tarrago et al., 2000 | BD, IR | IgG, IgM | Abbott Laboratories |
|  | Lopez-Fabal et al., 2015 | GP |  | Bioelisa |
| Sweden |  |  |  |  |
|  | Norder et al., 2016 | BD, TPG | IgG, IgM | Mikrogen recomWell, DS EIA, Euroimmun, Axiom, dia.Pro, |
|  | Olsen et al., 2006 | GP, IR | IgG | Abbott Laboratories |
|  | Sylvan et al., 1998 | GP, TPG | IgG | Abbott Laboratories |
| Switzerland |  |  |  |  |
|  | Kaufmann et al., 2011 | BD | IgG | MP Biomedical |
|  | Lavanchy et al., 1994 | BD, TPG, PW, IR | IgG | Abbott Laboratories |
|  | Niederhauser et al., 2016 | BD | IgG | Wantai |
| UK |  |  |  |  |
|  | Beale et al., 2011 | BD | IgG | Wantai |
|  | Bendall et al., 2010 | BD | IgG | Wantai, MPBiomedical |
|  | Cleland et al., 2013 | BD | IgG, IgM | Wantai |
|  | Dalton et al., 2011 | GP, TPG | IgG | Wantai |
|  | Dalton et al., 2008 | BD | IgG | Wantai |
|  | Ijaz et al., 2009 | GP | IgG | Fortress Diagnostics |
| USA |  |  |  |  |
|  | Atiq et al., 2009 | GP, TPG | IgG, IgM | MP Biomedical |
|  | Dawson et al., 1992 | BD,GP | IgG | In-house assay |
|  | Ditah et al., 2014 | GP | IgG | DSI |
|  | Dong et al., 2011 | BD | IgG | In-house assay, DSI |
|  | Engle et al., 2002 | BD, IR | IgG | In-house assay |
|  | Karetnyi et al., 1999 | BD, IR, TPG | IgG, IgM | In-house assay |
|  | Kuniholm et al., 2009 | GP | IgG | In-house assay |
|  | Mast et al., 1997 | BD | IgG | In-house assay, MP Biomedical (Genelabs) |
|  | Meng et al., 2002 | BD, IR | IgG | In-house assay |
|  | Obriadina et al., 2002 | BD | IgG | In-house assay |
|  | Ooi et al., 1999 | GP, IR | IgG, IgM | In-house assay |
|  | Stramer et al., 2016 | BD | IgG, IgM | MP Biomedical (Genelabs) |
|  | Xu et al., 2013 | BD, TPG | IgG, IgM | Wantai |
|  | Teshale et al., 2015 | GP | IgG | DSI |

**^1^** BD = blood donors; GP = general population; IR = group potentially at increased risk of exposure to HEV; TPG = targeted patient group ^2^  DSI = DSI s.r.l.
