# Supplementary material for: Systematic review and meta-analysis of the seroprevalence of hepatitis E virus in the general population across non-endemic countries
Source: PLoS One. 2019 Jun 7;14(6):e0216826. doi: 10.1371/journal.pone.0216826 (PMC6555507; doi:10.1371/journal.pone.0216826)
Supplement: S2 Table — (DOCX) [file pone.0216826.s002.docx]

# S2 Table. Risk of Bias assessment of studies investigating HEV IgG seroprevalence in blood donors or the general population

| **Country** | **References** | **Sample storage adequate^1^** | **Representative of study group justified^1^** | **Individual sampling strategy^1^** | **ROB from selective reporting^3^** | **ROB from confounding** | **Overall ROB** |
| --- | --- | --- | --- | --- | --- | --- | --- |
| Argentina |  |  |  |  |  |  |  |
|  | Debes et al., 2016 | N | Y | Con | U | U | U |
|  | Munne et al., 2014 | N | NR | Con | L | H | H |
|  | Rey et al., 1997 | N | Y | Con | L | U | U |
| Australia |  |  |  |  |  |  |  |
|  | Moaven et al., 1995 | N | Y | Con | L | H | H |
|  | Shrestha et al., 2014 | N | NR | Con | L | U | L |
| Austria |  |  |  |  |  |  |  |
|  | Fischer et al., 2015 | N | NR | Ran | L | U | L |
|  | Lagler et al., 2014 | N | Y | Con | L | U | U |
| Canada |  |  |  |  |  |  |  |
|  | Fearon et al., 2017 | N | Y | Con | L | U | L |
| Croatia |  |  |  |  |  |  |  |
|  | Vilibic-Cavlek et al., 2016 | N | NR | Con | L | U | U |
|  | Miletic Lovric et al., 2014 | N | NR | Con | L | U | L |
| Czech Republic |  |  |  |  |  |  |  |
|  | Strakova et al., 2014 | N | Y | RR | U | U | U |
|  | Nemecek et al., 2015 | N | NR | Con | L | U | L |
| Denmark |  |  |  |  |  |  |  |
|  | Christensen et al., 2002 | N | Y | Con | L | L | L |
|  | Holm et al., 2015 | N | NR | Con | L | U | L |
| France |  |  |  |  |  |  |  |
|  | Boutrouille et al., 2007 | N | NR | Con | L | U | L |
|  | Carpentier et al., 2012 | N | N | Con | L | U | L |
|  | Chaussade et al., 2013 | N | NR | Con | U | U | U |
|  | Coursaget al., 1994 | N | NR | Con | U | U | U |
|  | Gallian et al., 2014 | N | NR | WR | L | U | L |
|  | Izopet et al., 2015 | N | NR | Con | L | U | L |
|  | Mansuy et al., 2016 | Y | NR | Con | L | U | L |
|  | Mansuy et al., 2015 | N | NR | RR | L | U | L |
|  | Mansuy et al., 2011 | N | NR | Con | L | U | L |
|  | Mansuy et al., 2008 | N | Y | Con | L | U | L |
|  | Dalton et al., 2010 | N | NR | Con | L | U | L |
| Germany |  |  |  |  |  |  |  |
|  | Baylis et al., 2010 | N | NR | Con | L | U | L |
|  | Dawson et al., 1992 | N | Y | Con | L | U | U |
|  | Dremsek et al., 2012 | N | NR | Con | L | L | L |
|  | Faber et al., 2012 | Y | NR | WR | L | L | L |
|  | Juhl et al., 2014 | N | Y | Con | L | U | L |
|  | Krumbholz et al., 2014b | N | NR | RR | L | U | L |
|  | Krumbholz et al., 2012 | N | NR | Con | L | U | U |
|  | Pischke et al., 2014 | N | NR | Con | L | H | H |
|  | Pischke et al., 2011 | N | NR | Con | L | H | H |
|  | Reinheimer et al., 2012 | N | NR | Con | L | U | L |
|  | Vollmer et al., 2012 | N | NR | Con | L | U | L |
|  | Wenzel et al., 2013 | N | NR | Con | L | U | U |
|  | Pischke et al., 2010b | N | NR | Con | L | U | L |
|  | Pischke et al., 2013 | N | NR | Con | L | U | L |
| Greece |  |  |  |  |  |  |  |
|  | Dalekos et al., 1998 | N | Y | Con | L | U | L |
|  | Pittaras et al., 2014 | N | NR | Con | L | U | L |
|  | Psichogiou et al., 1996 | N | NR | Con | H | U | H |
|  | Zervou et al., 2015 | N | NR | Con | L | U | L |
| Hong Kong |  |  |  |  |  |  |  |
|  | Lok et al., 1992 | N | Y | Con | U | U | U |
|  | Wong et al., 2004 | Y | NR | RR | L | L | L |
| Iceland |  |  |  |  |  |  |  |
|  | Löve et al., 2018 | N | NR | Con | U | U | U |
| Ireland |  |  |  |  |  |  |  |
|  | O'Riordan et al., 2016 | N | Y | Con | L | U | L |
|  | Hickey et al., 2016 | Y | NR | RR | L | U | U |
| Israel |  |  |  |  |  |  |  |
|  | Mor et al., 2015 | N | NR | Con | L | H | H |
|  | Keretnyi et al., 1996 | N | NR | Con | L | U | L |
| Italy |  |  |  |  |  |  |  |
|  | Caruso et al., 2016 | N | Y | Con | L | U | L |
|  | Gessoni et al., 1996 | N | N | Con | L | U | L |
|  | Lucarelli et al., 2016 | N | NR | Con | L | U | L |
|  | Masia et al., 2009 | N | NR | Con | L | U | L |
|  | Pavia et al., 1998 | N | Y | WR | L | U | L |
|  | Puttini et al., 2015 | N | Y | Con, RR | H | U | H |
|  | Rapicetta et al., 2013 | N | NR | Con | H | L | H |
|  | Ricco et al., 2016 | N | Y | Con | L | U | L |
|  | Scotto et al., 2014 | N | NR | Con | L | U | **U** |
|  | Scotto et al., 2012 | N | NR | Con | U | U | U |
|  | Zanetti et al., 1994 | N | Y | Con | L | U | U |
|  | Gessoni et al., 1998 | N | Y | Con | L | U | L |
| Japan |  |  |  |  |  |  |  |
|  | Fukuda et al., 2007 | N | NR | Con | L | U | L |
|  | Fukuda et al., 2004 | N | NR | S | L | U | L |
|  | Gotanda et al., 2007 | N | NR | WR | U | U | L |
|  | Li et al., 2000 | N | NR | Con | U | U | U |
|  | Mitsui et al., 2005 | N | NR | Con | L | U | L |
|  | Sakata et al., 2008 | N | Y | WR | L | U | L |
|  | Takahashi et al., 2010 | Y | NR | WR | L | U | L |
|  | Takahashi et al., 2005 | N | NR | Con | L | U | U |
|  | Takeda et al., 2010 | Y | Y | WR | L | U | L |
|  | Tanaka et al., 2005 | NR | N | RR | L | U | U |
|  | Tei et al., 2004 | N | Y | Con | L | U | H |
|  | Toyoda et al., 2008b | N | NR | Con | U | U | U |
|  | Ding et al., 2003 | N | Y | Con | L | U | L |
|  | Fukae et al., 2016 | Y | Y | Con | L | U | U |
| South Korea |  |  |  |  |  |  |  |
|  | Ahn et al., 2005 | N | Y | Con | L | U | L |
|  | Choi et al., 2003 | Y | Y | RR | U | U | U |
|  | Park et al., 2012 | Y | Y | R | L | U | L |
|  | Yoon et al., 2014 | Y | Y | RR | L | L | L |
| Netherlands |  | N | Y | Con | L | U | U |
|  | Bouwknegt et al., 2008 | Y | Y | Con | L | L | L |
|  | Herremans et al., 2007a | N | NR | Con | L | U | L |
|  | Herremans et al., 2007b | N | NR | Con | L | U | L |
|  | Hogema et al., 2014 | N | N | Con | L | U | L |
|  | Sadik et al., 2016 | N | Y | RR | L | L | L |
|  | Slot et al., 2013 | Y | NR | Con | L | U | L |
|  | Van den Berg et al., 2014 | N | Y | Con | L | U | U |
|  | Verhoef et al., 2012 | Y | Y | RR | L | L | L |
|  | Zaaijer et al., 1995 | N | NR | RR | L | U | L |
|  | Zaaijer et al., 1992 | N | NR | Con | U | U | U |
| New Zealand |  |  |  |  |  |  |  |
|  | Dalton et al., 2007 | N | NR | Con | L | U | L |
| Norway |  |  |  |  |  |  |  |
|  | Andenaes et al., 2000 | Y | NR | Con | L | U | L |
|  | Lange et al., 2017 | N | Y | Con | L | U | U |
| Poland |  |  |  |  |  |  |  |
|  | Bukowska et al., 2016 | N | NR | Con | L | U | L |
|  | Sulkowska et al., 2016 | N | NR | Con | L | U | L |
| Portugal |  |  |  |  |  |  |  |
|  | Mesquita et al., 2014 | N | NR | Con | L | U | U |
|  | Sargento et al., 2016 | N | NR | Con | L | U | L |
|  | Teixeira et al., 2017 | N | NR | Con | L | U | U |
|  | Pereira et al., 2016 | Y | NR | Con | L | U | L |
|  | Sargento et al., 2014 | N | NR | Con | L | U | L |
| Qatar |  |  |  |  |  |  |  |
|  | Nasrallah et al., 2016 | N | NR | Con | L | U | L |
| Russia |  |  |  |  |  |  |  |
|  | Obriadina et al., 2002 | N | NR | Con | L | U | L |
| Saudi Arabia |  |  |  |  |  |  |  |
|  | Abdelaal et al., 1998 | Y | Y | s | L | L | L |
|  | Arif et al., 1994 | Y | Y | Con | L | L | L |
|  | Ayoola et al., 2002 | N | NR | Con | U | L | L |
|  | Johargy et al., 2013 | N | Y | RR | L | L | L |
|  | Paul et al., 1994 | N | NR | Con | L | H | H |
|  | Elsheikh et al., 2012 | N | NR | Con | L | U | L |
| Spain |  |  |  |  |  |  |  |
|  | Buti et al., 2006 | N | N | RR | L | L | L |
|  | Buti et al., 1995 | N | NR | Con | L | U | L |
|  | Fogeda et al., 2012 | Y | N | WR | L | U | L |
|  | Mateos et al., 1999 | N | NR | Con | H | H | H |
|  | Medrano et al., 1995 | N | NR | Con | L | U | L |
|  | Riveiro-Barciela et al., 2014 | N | NR | WR | L | U | L |
|  | Sauleda et al., 2015 | N | Y | Con | L | U | L |
|  | Tarrago et al., 2000 | N | NR | Con | U | U | U |
|  | Lopez-Fabal et al., 2015 |  |  |  |  |  |  |
|  | Sauleda et al. |  |  |  |  |  |  |
| Sweden |  |  |  |  |  |  |  |
|  | Norder et al., 2016 | N | Y | S | L | U | L |
|  | Olsen et al., 2006 | N | NR | Con | L | H | H |
|  | Sylvan et al., 1998 | Y | Y | Con | L | U | L |
| Switzerland |  |  |  |  |  |  |  |
|  | Kaubfman et al., 2011 |  |  |  |  |  |  |
|  | Schnegg et al., 2013 | N | Y | Con | L | U | L |
|  | Lavanchy et al., 1994 | N | NR | Con | L | U | U |
|  | Niederhauser et al., 2016 | N | NR | Con | L | U | L |
| UAE |  |  |  |  |  |  |  |
|  | Chibber et al., 2004 | Y | Y | WR | L | U | L |
|  | Kumar et al., 2001 | Y | Y | Con | L | L | L |
| UK |  |  |  |  |  |  |  |
|  | Beale et al., 2011 | N | NR | Con | U | U | U |
|  | Bendall et al., 2010 | N | NR | Con | L | U | L |
|  | Cleland et al., 2013 | N | NR | Con | L | U | L |
|  | Dalton et al., 2011 | N | NR | Con | L | L | L |
|  | Dalton et al., 2008a | N | Y | Con | L | L | L |
|  | Ijaz et al., 2009 | N | NR | Con | L | U | L |
| USA |  |  |  |  |  |  |  |
|  | Atiq et al., 2009 | N | NR | Con | L | L | L |
|  | Dawson et al., 1992 | N | NR | Con | L | U | U |
|  | Ditah et al., 2014 | Y | NR | R | L | L | L |
|  | Dong et al., 2011 | N | NR | Con | L | U | L |
|  | Engle et al., 2002 | N | NR | Con | L | U | L |
|  | Karetnyi et al., 1999 | N | NR | Con | L | U | L |
|  | Kuniholm et al., 2009 | Y | NR | WR | L | L | L |
|  | Mast et al., 1997 | N | NR | S | H | L | H |
|  | Meng et al., 2002 | N | NR | Con | L | U | U |
|  | Obriadina et al., 2002 | N | NR | Con | L | U | L |
|  | Ooi et al., 1999 | N | Y | Con | L | H | H |
|  | Stramer et al., 2016 | Y | Y | RR | L | U | U |
|  | Xu et al., 2013 | N | NR | WR | L | L | L |
|  | Teshale et al., 2015 | Y | NR | RR | L | L | L |

**^1^** Y = Yes, N = No, NR = Not reported

**^2^** Con = Convenience, R = Random, RR= Reported random, S = systematic

^3^ L = Low H – High U = Unclear
