# Supplementary material for: Systematic review and meta-analysis of the seroprevalence of hepatitis E virus in the general population across non-endemic countries
Source: PLoS One. 2019 Jun 7;14(6):e0216826. doi: 10.1371/journal.pone.0216826 (PMC6555507; doi:10.1371/journal.pone.0216826)
Supplement: S1 File — (DOCX) [file pone.0216826.s003.docx]

# S1 File. Study protocol

**1. Title:** **Protocol for the systematic review of human hepatitis E virus (HEV) seroprevalence and its predictors, in selected locations**

**2. Authors**: Lisa Waddell, Judy Greig, Ian Young, Barb Wilhelm

**3. Background**

Since Balayan et al. (1990) reported the successful propagation of hepatitis E virus (HEV) in pigs, animals have been investigated as potential human exposure sources. Subsequent work identified four widely recognized genotypes, with genotypes 1 and 2 detected only in humans, generally occurring in large waterborne outbreaks in countries with limited public health infrastructure. In contrast, genotypes 3 and 4, and more recently, proposed genotype 7, have been detected in humans and a variety of other animals including pigs, wild boar, deer, rodents, and camels (Lee et al., 2015; Meng, 2010).

Human sero-surveys suggest that particularly in countries in which locally acquired (as opposed to travel-related) HEV infection may be by potentially zoonotic genotypes (e.g. genotypes 3, 4, or 7), asymptomatic infection is much more common than clinical disease (Petrik et al., 2016). Over the past five years the potential for asymptomatically infected humans to donate blood while HEV viremic and potentially contaminate the blood supply, has been increasingly investigated globally. These human sero-surveys show a proportion of the population has been exposed to HEV as demonstrated by the presence of HEV IgG antibodies; this proportion seems to vary across study locations (Petrik et al., 2016). While pigs and pork are widely cited as potential human HEV exposure sources, the sources of human HEV infection and reasons for apparent variation in human HEV exposure across countries, and even regions within countries, remain unclear (Petrik et al., 2016).

The interpretation of [HEV] seroprevalence data is immensely challenging for several reasons. These challenges include the lack of comparability of results from the different assays, high seroprevalence in populations where disease is rare or never reported, the presence of multiple genotypes with different disease patterns and inability of serological tests to distinguish between genotypes, and lack of data for reliable mathematical modelling to determine disease burden from seroprevalence. Furthermore, the majority of seroprevalence studies do not involve a representative sample of any population making it difficult to infer prevalence and trends to the population.

(Hepatitis E vaccine working group of the World Health Organization, 2014)

**4. Objectives**

**4.1 Research Question**

**Does the reported seroprevalence of HEV in humans in locations in which locally acquired human HEV infections are potentially zoonotic (hereafter called study locations) vary significantly, and how much of this variation can be explained by the literature on sources of HEV exposure for humans?**

**4.2 Sub-questions**

**i. What is the seroprevalence of human HEV IgG or IgM antibodies in study locations, and does this vary significantly across individual study locations?**

Issue 1: defining study locations. This review will define study locations as those countries which are categorized as ‘very high human development’ by the United Nations (UN) Development Program (Appendix 1).

Analysis plan: Human HEV sero-survey data will be extracted from relevant papers, and the effects of location/ sampling frame characteristics/time /assay will be examined as explanatory variables for heterogeneity between survey results.

Issue 2: The studies prioritized for analysis will be sero-prevalence surveys of healthy populations. Surveys of other groups such as liver patients or the immune-compromised may be appraised to provide context but will not directly provide evidence supporting the research question.

**ii. What evidence exists on the prevalence of potentially zoonotic HEV genotypes in potential human exposure sources?**

- What are the reported potential exposure sources (e.g. HEV-contaminated pork) of human HEV infection in these locations?
- What is the prevalence of HEV contamination or shedding, for these exposures?

**iii. Does the prevalence of HEV shedding/contamination, density (e.g. swine), or consumption patterns (e.g. pork) of potential sources of human HEV exposure explain the variation in human HEV seroprevalence across study locations?**

- Is there evidence for a significant association between presence or prevalence of any of the potential exposures, and human HEV sero-prevalence in the same study location? (This could include risk factor studies examining associations with various HEV exposures for humans.)

**iv. What are the data gaps encountered in trying to understand the relationship between HEV exposure in humans and potential sources of HEV?**

**4.3 Outputs**

1. Systematic review and meta-analysis summary estimates (where appropriate) of human HEV seroprevalence in HEV study locations.
2. Systematic review and MA estimates of HEV detection in human exposure sources, across study locations.
3. Investigation of potential association between seroprevalence and prevalence of putative exposure sources.
4. A summary of findings fact sheet reporting findings of sub-questions i and ii.
5. A repository and dataset of all relevant literature captured in this study.

**5.0 Methods**

**5.1 Review team and responsibilities**

| **Member** | **Organization** | **Project Role** |
| --- | --- | --- |
| Lisa Waddell | RISK - Guelph | Research synthesis expert |
| Judy Greig | RISK - Guelph | Research synthesis expert |
| Ian Young | Ryerson University, Toronto | Research synthesis expert |
| Anton Andonov | National Microbiology Laboratory, Winnipeg | Topic expert- HEV diagnostic assays |
| Margaret Fearon | Canadian Blood Services | Topic expert- human blood collection/donation and sampling frames |
| Barb Wilhelm | Big Sky Health Analytics | Research synthesis expert and topic expert |

**5.2 Question scope defined using the CoCoPop acronym**

**Population:** the target population is the general population of a given study location. Study populations may include blood donors, patients with or without clinical hepatitis, or high-risk groups, both for HEV sero-conversion (e.g. farm workers), and for development of clinical Hepatitis E (e.g. immune-compromised patients). Travellers, recent immigrants, and traveling members of armed forces were excluded from the SR due to the difficulty in establishing the country of origin of infection in these groups. Liver patients were categorically excluded from this SR, as were groups consisting of only Hepatitis E patients. Data from defined sub-groups of the general population, potentially differing from the general population regarding their probability of HEV exposure, (e.g. farmers or targeted patient groups such as hemophiliacs), were captured in the overall SR, but will be analysed and reported separately from the general population.

**Condition** (outcome of interest): Measurement of HEV IgG antibodies was deemed relevant. Total HEV antibodies, IgM antibodies, and detection of HEV RNA (e.g. using RT-PCR) were deemed not relevant outcomes for this review. Included studies were required to employ a defined, reproducible assay.

**Context:** Environmental factors can have a substantial impact on the prevalence or incidence of a condition. Some demographic descriptors have been associated with odds of HEV sero-positivity including socio-economic status [23], occupation [24], recreational activities [9], dietary preferences [11], and rural, relative to urban, residence [25]. Therefore, these parameters were captured when reported by investigators. A complete list of contextual parameters captured is listed in the data extraction tool available in S3.

**Study designs:** Relevant designs include prevalence surveys, longitudinal prevalence studies, cohort, cross-sectional and case-control studies, intervention studies in which a control group did not receive the intervention of interest and exposure to HEV was not controlled, but was assayed for an outcome of interest, investigations of relevant diagnostic tests in which relevant populations were assayed**.**

**Inclusion criteria:** primary research published in English, French, or Spanish, reporting the investigation of a group of human subjects for HEV seroprevalence or sources of HEV exposure within included study locations.

**Exclusion criteria:** Animal studies; laboratory studies focusing on pathogenesis of disease; studies which pooled data on multiple groups and data from various groups could not be separated; studies reporting inadequate detail regarding methods (see screening tools), and studies only examining HEV genetic sequence. In addition, multi-country studies where contribution of specimens from each country to the study is unclear; studies among travelers from one country to other countries (except when people from one country had clearly travelled to another country as a group and had been studied as such); Studies reporting the same data already reported in another published study. Studies originating from countries not categorized ‘very high human development’ by the United Nations (UN) Development Programme (UN, 2015).

**5.3 Search strategy**

The literature search strategy will include the evaluation of all citations captured through the implementation of the search algorithm in several electronic bibliographic databases, a grey literature search and search verification protocol to ensure that all primary literature is identified for this review. The **grey literature search** will include hand-searching selected conference proceedings and a complementary internet search conducted in Google and Yahoo using the same algorithm used in the bibliographic database search. This will identify relevant research not indexed in peer-reviewed literature bibliographic databases**. Search verification** will be conducted by hand-searching the reference lists of captured reviews and risk assessments for relevant research not identified by the electronic search.

**5.3.1. Algorithms**

A search algorithm specific to each sub-question will be developed and pretested prior to being executed. Therefore an algorithm will be designed to capture studies investigating i) human HEV sero-prevalence, and ii) investigating potential HEV exposures for humans, such as HEV detection in pigs, or retail pork, or other domestic animals, or wildlife.

**5.3.2 Databases**

Selection of appropriate databases could be guided by the topic experts. These will include Pubmed, Scopus, EMBASE, and Web of Science, for question (i), and also CAB, Africola, and Agris for questions (ii) and (iii).

**Electronic databases and grey literature search strategy**

Search Documentation – Nov 29, 2016

("hepatitis E virus" OR "Hepatitis E virus" OR HEV) AND (blood OR serum OR serology OR sero-prevalence OR plasma OR “plasma products”)

1. Embase
2. Pubmed
3. Scopus
4. Global Health
5. Epub Ahead of Print, In-Process & Other Non-Indexed Citations, Ovid MEDLINE(R) Daily and Ovid MEDLINE(R)

**Library/Database:** PubMed

**Date of Search:** November 29, 2016

**Search String:** ("hepatitis E virus" OR "Hepatitis E virus" OR HEV) AND (blood OR serum OR serology OR sero-prevalence OR plasma OR “plasma products”)

**# Hits:** 2618

**Library/Database:** Scopus

**Date of Search:** November 29, 2016

**Search String:** Article Title, Abstract, Keywords("hepatitis E virus" OR "Hepatitis E virus" OR HEV) AND (blood OR serum OR serology OR sero-prevalence OR plasma OR “plasma products”)

**# Hits:2786**

**Library/Database:  Embase**1974 to 2016 November 28**,**[
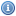
](http://ovidsp.tx.ovid.com/sp-3.22.1b/ovidweb.cgi?&S=OHMDFPKGGCDDCMDFNCHKJAJCEMJAAA00&Database+Field+Guide=9)**Global Health**1973 to 2016 Week 46

**Date of Search:** November 29, 2016

**Search String:** All Fields ("hepatitis E virus" OR "Hepatitis E virus" OR HEV) AND (blood OR serum OR serology OR sero-prevalence OR plasma OR “plasma products”)

**# Hits:**

**Library/Database: Epub Ahead of Print, In-Process & Other Non-Indexed Citations, Ovid MEDLINE(R) Daily and Ovid MEDLINE(R)**1946 to Present

**Date of Search:** November 29, 2016

**Search String:** All Fields ("hepatitis E virus" OR "Hepatitis E virus" OR HEV) AND (blood OR serum OR serology OR sero-prevalence OR plasma OR “plasma products”)

**# Hits:** 3549

**# Hits:**

Total # of citations prior to de-duplication: 12367

Total # of citations after 1^st^ round of de-duplication in Endnote: 4467

Total # of citations after 2^nd^ round of de-duplication in Refworks/DistillerSR:

Total # citations added from search verification =

Total # citations added from the grey literature search =

**5.3.3 Grey literature search strategy**

An internet search will be conducted in Google and Yahoo using the same terms as the database search to identify relevant work not published in peer reviewed journals. Selected conference proceedings will be hand-searched to identify relevant work. The selection of conferences will be guided by the topic experts but could include Annual Meeting of the EASL (European Association for the Study of the Liver), European Congress of Clinical Microbiology and Infectious Diseases, World Conference on Infectious Diseases, and IDWeek.

**5.3.4 Search verification**

The reference lists of 10 or more randomly selected reviews, captured by the search strategy, will be hand searched to identify additional relevant citations. Relevant studies not already captured by the search will be uploaded and reviewed on Distiller.

**5.3.5 Review management**

The search strategy will be compiled and de-duplicated in a RefWorks database. This database will then be exported to DistillerSR, which is a web-based systematic review software designed to manage all stages of conducting systematic reviews. All stages of the scoping study from relevance screening to data extraction will be conducted within this software. The final dataset will be exported into MS Excel, cleaned and prepared for descriptive and analytical analysis in Stata.

Although the review has one over-arching question, it will be executed in two stages, with the first stage implementing the search string and tools relevant to the first sub-question (4.2( i.)) inquiring Does HEV sero-prevalence vary significantly across study locations. If findings support an answer in the affirmative, then the second question pertaining to potential predictors will be investigated.

**5.3.6 Data analysis**

**Part 1** – characterize studies captured by region, country, year, age, other characteristics, assay

- consult re what studies might be pooled

- Stratify

- MA estimates by country

- MR – region/country/assay?

**Part 2** – characterize studies by location, year, sampling frame characteristics (e.g. age, occupation, health status, etc.), assay

- Summarize and potentially analyse the data on predictor prevalence and association data by country
- Depending on the quantity of data we can try to explain the heterogeneity between studies by accounting for predictors such as country/population/assay used etc.

**6.0 References**

Balayan, M.S., R.K. Usmanov, N.A. Zamyatina, D.I. Djumalieva, F.R., Karas, 1990: Brief report: experimental hepatitis E infection in domestic pigs. J. Med. Virol. 32, 58-59.

Lee, G.H., Tan, B.H., Teo, E.C., Lim, S.G., Dan, Y.Y., Wee, A., Aw, P.P., Zhu, Y., Hibberd, M.L., Tan, C.K., Purdy, M.A., Teo, C.G. 2015. [Chronic infection with camelid hepatitis E virus in a liver transplant recipient who regularly consumes camel meat and milk.](https://www.ncbi.nlm.nih.gov/pubmed/26551551) Gastroenterology. 150, 355-357.e3. doi: 10.1053/j.gastro.2015.10.048.

Meng, X.-J. 2010: Hepatitis E virus: animal reservoirs and zoonotic risk. Vet. Microbiol. 140, 256-265.

Petrik, J., M. Lozano, C.R. Seed, H.M. Faddy, A.J. Keller, P.S. Prado Scuracchio, S. Wendel, A. Andonov, M. Fearon, G. Delage, J. Zhang, J.W. Shih, P. Gallian, R. Djoudi ,P. Tiberghien, J. Izopet, J. Dreier, T. Vollmer, C. Knabbe, R. Aggarwal, A. Goel, A.R. Ciccaglione, K. Matsubayashi, M. Satake, K. Tadokoro, S.H. Jeong, H.L. Zaaijer, E. Zhiburt, J. Chay, D. Teo, S.S. Chua, M. Piron, S. Sauleda, J.M. Echevarría, H. Dalton, S.L. Stramer, 2016: Hepatitis E. Vox Sang. 110, 93-130. doi: 10.1111/vox.12285. PMID: 26198159.

United Nations Development Programme. 2015. Human Development Report 2015: Work for human development. Retrieved from: [http://hdr.undp.org/sites/default/files/2015_human_development_report.pdf Accessed 13 January 2017](http://hdr.undp.org/sites/default/files/2015_human_development_report.pdf%20%20%20%20%20%20%20%20%20%20%20%20%20%20%20%20%20%20%20%20%20%20%20%20%20%20%20%20%20%20%20%20%20%20%20%20%20%20Accessed%2013%20January%202017)
