# Supplementary material for: Systematic review and meta-analysis of the seroprevalence of hepatitis E virus in the general population across non-endemic countries
Source: PLoS One. 2019 Jun 7;14(6):e0216826. doi: 10.1371/journal.pone.0216826 (PMC6555507; doi:10.1371/journal.pone.0216826)
Supplement: S2 File — (DOCX) [file pone.0216826.s004.docx]

# S2 File. Systematic Review tools

**1. Relevance screening tool level 1**- performed on citation

| **Question** | **Options** | **Definitions/notes** |
| --- | --- | --- |
| 1. Is the work described in this citation primary research investigating **human HEV serology?** | **a. Yes**. Relevant primary research  b. No- Relevant review  c. No- Relevant risk assessment, commentary  d. No. BUT does describe investigation of potential human HEV exposure source  e. No. None of the above | **Human HEV Serology**- including assay of human whole blood/serum/plasma/plasma products, for HEV IgG or IgM antibodies.  d. Including studies of prevalence of HEV RNA detection in pigs, pork, produce, water, wildlife |

If Q1= a THEN promote to level 2

**2. Relevance screening tool 2 – initial categorization**

Performed on abstract

| **Questions** | **Options** | **Notes** |
| --- | --- | --- |
| **1.** Was this study conducted in a relevant study location (see list)? | a. Yes. (Include)  b. No. Please specify. (Exclude)  c. Can’t tell (neutral) |  |
| **2.** Is the sampling frame derived from a healthy population (e.g. healthy individuals screened for HEV, or healthy blood donors). Please check all that apply. | a. Yes. Potentially representative of general population, or at least not members of other groups listed below. (Include)  b. Yes. Group considered at greater risk of HEV exposure relative to general population. Please specify. (Include)  c. No. Immuno-compromised. Please specify. (Include)  d. No. ONLY patients with liver disease other than cancer. (Exclude)  e. No. ONLY outbreak data (Exclude)  f. No. ONLY other. Please specify. (Exclude) | **a. Potentially representative of general population**: e.g. healthy individuals screened for HEV, or blood donors. Some study groups are likely not perfectly representative of general population (e.g. armed forces employees, pregnant mothers) but cannot be categorized in any of the other options listed based on information presented in the abstract.  **b. Considered high risk for HEV exposure**. E.g. people who work or participate in recreation in the outdoors, people who work with animals alive (farmers) or dead (slaughterhouse workers).  **c. Immuno-compromised**: e.g. patients with lympho-proliferative disorders, transplant patients, subjects infected with HIV.  **d. Only liver patients**: e.g. with acute or chronic liver failure, elevated liver enzymes as defined by authors. However, if the study samples a group of patients, with liver cancer please indicate ‘immuno-compromised’.  **f. ONLY other**. E.g. purposively selected for HEV infection, or for some clinical disease. If all subjects are HEV positive patients of various sorts (e.g.including cancer and liver), please just indicate ‘other’. |
| **3.** Does this study investigate the accuracy of a diagnostic assay for HEV infection, or apply more than one assay targeting the same outcome (e.g. IgM) to the same group of subjects? | a. Yes, antibodies. (Neutral)  b. Yes, RNA. (Neutral)  c. No. (Neutral) | **a.** Studies measuring both IgM and IgG would be considered to be measuring two different outcomes.  Confirmation of ‘positives’ by another test e.g. Western blot would not be considered investigation of the assay.  **c.** If unclear, please indicate c ‘No’. |

**Notes**

**Yes: Andorra, Argentina, Australia, Austria, Bahrain, Belgium, Brunei, Canada, Chile, Croatia, Cyprus, Czech Republic, Denmark, Finland, France, Germany, Greece, Hong Kong, Hungary, Iceland, Ireland, Israel, Italy, Japan, South Korea (aka Republic of Korea), Kuwait, Latvia, Liechtenstein, Lithuania, Luxembourg, Malta, Montenegro, Netherlands, New Zealand, Norway, Poland, Portugal, Qatar, Saudi Arabia, Slovakia, Slovenia, Spain, Sweden, Switzerland, United Arab Emirates, United Kingdom, United States.**

**No = any country not listed under ‘yes’**

If((Q1 =a (Yes)) AND (Q2 = a OR b OR c)) THEN promote to level 3 (DCU).

Pretest refids

4403,4408,4412,4416,6010,6035,6042,6043,6048,6090,6095,6108,6138,6147,9528,9533,9552,9556,9561,9567

**3. Relevance confirmation/categorization and quality assessment tool** performed on full paper

**HEV_sero_DCU 24 April/17**

| **QUESTIONS** | **OPTIONS** | **NOTES** |
| --- | --- | --- |
| **1. Does the work described in this paper investigate human HEV serology?** | a. Yes. Relevant primary research  b. No- Relevant review or commentary  c. No- Relevant risk assessment  d. No. BUT does describe investigation of potential human exposure source  e. No. No potential relevance to overall question | **Human HEV Serology**- including assay of whole blood/serum/plasma/plasma products, for HEV IgG or IgM antibodies  **If ‘a’ , include; all other options exclude** |
| **2. How were human subjects sampled?** | a. One i.e. case report. Please stop now and submit your work.  b. More than one subject sampled. |  |
| **3. What was the continent from which the sampling frame was drawn from?**  (Please check all that apply) | Continent:  a. North America  b. Europe  c. Australasia  d. Central and South America/ Caribbean e. Asia  f. Not stated (exclude) | **North America:** Canada, USA and Mexico  **Central/South America/ Caribbean:** Caribbean, and all of Central/South America.  **Europe:** includes, Belarus, Latvia, Ukraine, Estonia, Cyprus & west (incl. Iceland and Greenland)  **Asia:** Russia, Turkey, middle eastern countries and east  **Australasia** is limited to Australia, New Guinea, New Zealand, New Caledonia, and neighbouring islands, including the Indonesian islands |
| **4. What was the country from which the sampling frame was drawn? Please check all that apply.** | - **Andorra,** - **Argentina,** - **Australia,** - **Austria,** - **Bahrain,** - **Belgium,** - **Brunei,** - **Canada,** - **Chile,** - **Croatia,** - **Cyprus,** - **Czech Republic,** - **Denmark,** - **Estonia** - **Finland,** - **France,** - **Germany,** - **Greece,** - **Hong Kong,** - **Hungary,** - **Iceland,** - **Ireland,** - **Israel,** - **Italy,** - **Japan,** - **South Korea (aka Republic of Korea),** - **Kuwait,** - **Latvia,** - **Liechtenstein,** - **Lithuania,** - **Luxembourg,** - **Malta,** - **Montenegro,** - **Netherlands,** - **New Zealand,** - **Norway,** - **Poland,** - **Portugal,** - **Qatar,** - **Romania** - **Russia** - **Saudi Arabia,** - **Singapore** - **Slovakia,** - **Slovenia,** - **Spain,** - **Sweden,** - **Switzerland,** - **United Arab Emirates,** - **United Kingdom,** - **United States.** - **None of the above (exclude): _____** |  |
| **5. For which of the following HEV outcomes is extractable prevalence data available in this study?** (Please indicate all which apply) | a. IgG antibodies  b. IgM antibodies  c. HEV RNA detection ie PCR  d. Other. Please specify _______  e. no extractable data (exclude) | Studies investigating antibody sero-prevalence may study either or both of IgG/IgM |
| **6. In what language is this work published?** | a. English  b. French  c. Spanish  d. Other (exclude). Please specify _________ |  |
| **7. In what year(s) was the sampling described in this paper performed?**  (Please indicate all which apply) | a. 2015 or later  b. 2010-2014  c. 2005-2009  d. 2000-2004  e. before 2000  f. Can’t tell | This may not be clear from the paper, ergo next question |
| **8. In what year was this paper published?** | a. 2015 or later  b. 2010-2014  c. 2005-2009  d. 2000-2004  e. before 2000 |  |
| **9. Specify the study design.**  (Please indicate all which apply) | a. Prevalence survey  b. Cross-sectional survey  c. Cohort survey  d. Case-control study  e. Human case report or case series  f. Evaluation of the performance of a diagnostic test  g. Intervention study  h. Other. Please specify | **Prevalence survey:** A study that measures outcome (prevalence of organism) at a single point in time.  **Case-control study:** a group of cases, as defined by the investigator, and non-cases, are selected and compared with respect to the presence of the hypothesized risk factor.  **Cross-sectional study:** A study done at a single point in time to investigate the prevalence of organism and hypothesized risk factors within the study population  **Cohort study:** Group exposed to a hypothesized risk factor (exposure), and a group not exposed to the factor are selected and observed over the study period to record contamination with selected microorganism in each group.  **Human case report or case series:** a description of one or more cases, as defined by the investigator.  **Evaluation of diagnostic tests:** study with the expressed purpose of investigating diagnostic test performance. All studies in this review utilised diagnostic tests; few investigated their performance in the study currently reported.  **Intervention** to reduce prevalence/concentration of HEV: study investigating medical or management strategies to reduce viral prevalence or concentration. Please note these interventions should be ready for field use. **Our interest will lie in potential data extraction from the control group.** |
| **10. Were co-infections with other microbes investigated? Please check all that apply.** | a. Yes, HIV  b. Yes, HCV  c. Yes, other. Please specify ______  d. No. Only HEV was assayed. |  |
| **11. Does this study simultaneously sample animals, food, water or other possible local sources of HEV exposure?** | a. Yes, samples animals for HEV exposure (Ig’s)  b. Yes. Samples animals for HEV detection (RT_PCR)  c. Yes, studies genomic evidence comparing animal and human-derived HEV isolates  d. Yes. Other- please specify.__________  e. No. Only human evidence is investigated in this study. |  |
| **10. Which forms must be completed for this paper? Please check all that apply.** | a. QA_populations  b. Diag characterization |  |
| **11. Comments** |  |  |

**HEV sero level 3 QA-DE tool 10mar/17**

| **QUESTIONS** | **OPTIONS** | **NOTES** |
| --- | --- | --- |
| **RELEVANCE CONFIRMATION** |  |  |
|  |  |  |
| **1. Does the work described in this paper investigate human HEV serology?** | a. Yes. Relevant primary research  b. No- Relevant review or commentary  c. No- Relevant risk assessment,  d. No. BUT does describe investigation of potential human exposure source  e. No. No potential relevance to overall question | **Human HEV Serology**- including assay of whole blood/serum/plasma/plasma products, for HEV IgG or IgM antibodies  **If ‘a’ , include; all other options exclude** |
| **2. Please list the continent, country and region the sampling frame was drawn from?**  (Please indicate all which apply)  **Region:** specify ONLY if specified by the author. | Continent:  a. North America  b. Europe  c. Australasia  d. Central and South America/ Caribbean e. Asia  f. Not stated (exclude)  Country:   - **Andorra,** - **Argentina,** - **Australia,** - **Austria,** - **Bahrain,** - **Belgium,** - **Brunei,** - **Canada,** - **Chile,** - **Croatia,** - **Cyprus,** - **Czech Republic,** - **Denmark,** - **Finland,** - **France,** - **Germany,** - **Greece,** - **Hong Kong,** - **Hungary,** - **Iceland,** - **Ireland,** - **Israel,** - **Italy,** - **Japan,** - **South Korea (aka Republic of Korea),** - **Kuwait,** - **Latvia,** - **Liechtenstein,** - **Lithuania,** - **Luxembourg,** - **Malta,** - **Montenegro,** - **Netherlands,** - **New Zealand,** - **Norway,** - **Poland,** - **Portugal,** - **Qatar,** - **Saudi Arabia,** - **Slovakia,** - **Slovenia,** - **Spain,** - **Sweden,** - **Switzerland,** - **United Arab Emirates,** - **United Kingdom,** - **United States.** - **ONLY Not Relevant Country (exclude): _____**   Region:  Region(s) within country______ | **North America:** Canada, USA and Mexico  **Central/South America/ Caribbean:** Caribbean, and all of Central/South America.  **Europe:** includes, Belarus, Latvia, Ukraine, Estonia, Cyprus & west (incl. Iceland and Greenland)  **Asia:** Russia, Turkey, middle eastern countries and east  **Australasia** is limited to Australia, New Guinea, New Zealand, New Caledonia, and neighbouring islands, including the Indonesian islands |
| **3. For which of the following HEV outcomes is extractable prevalence data available in this study?** (Please indicate all which apply) | a. IgG antibodies  b. IgM antibodies  c. HEV RNA detection ie PCR  d. Other. Please specify _______  e. no extractable data (exclude) | Studies investigating antibody sero-prevalence e.g. with ELISA kits such as Wantai may study either or both of IgG/IgM |
| **4. In what language is this work published?** | a. English  b. French  c. Spanish  d. Other (exclude). Please specify _________ |  |
| **5. In what year(s) was the sampling described in this paper performed?**  (Please indicate all which apply) | 2016  2015  Etc  Not reported | *This may not be clear from the paper, ergo next question* |
| **6. In what year was this paper published?** | 2016  2015  etc |  |
| **7. Specify the study design.**  (Please indicate all which apply) | a. Prevalence survey  b. Case-control study  c. Cross-sectional study  d. Cohort study  e. Human case report or case series  f. Evaluation of the performance of a diagnostic test  g. Intervention study  h. Other. Please specify | **Prevalence survey:** A study that measures outcome (prevalence of organism) at a single point in time.  **Case-control study:** a group of cases, as defined by the investigator, and non-cases, are selected and compared with respect to the presence of the hypothesized risk factor.  **Cross-sectional study:** A study done at a single point in time to investigate the prevalence of organism and hypothesized risk factors within the study population  **Cohort study:** Group exposed to a hypothesized risk factor (exposure), and a group not exposed to the factor are selected and observed over the study period to record contamination with selected microorganism in each group.  **Human case report or case series:** a description of one or more cases, as defined by the investigator.  **INVESTIGATION of diagnostic tests:** study with the expressed purpose of investigating diagnostic test performance. All studies in this review utilised diagnostic tests; few investigated their performance in the study currently reported.  **Intervention** to reduce prevalence/concentration of HEV: study investigating medical or management strategies to reduce viral prevalence or concentration. Please note these interventions should be ready for field use. **Our interest will lie in potential data extraction from the control group.**  **Other**. Please specify (EXCLUSION) eg. studies of pathogenesis or pathogenicity. |
| **8. What is the population sampled? Please check all that apply.** | a. Blood donor survey  b. General population survey  c. Targeted patient group(s).  d. Pregnant women  e. Armed forces  f. Increased risk of HEV exposure  h. (Add additional categories as needed) | a. Survey of samples derived from individuals volunteering blood donation  b. Survey is intended to be representative of the study location’s general population  c. examples could include: liver/cell or organ recipient, HIV patients  f. e.g. farmers, veterinarians |
| **Targeted patient groups. Please check all that apply. (DROPDOWN)** | a. Liver patients  b. Cancer patients  c. Transplant patients  d. Hemodialysis patients  e. Frequent blood or blood product recipients  f. HIV positive patients  e. Other. Please specify. |  |
| **Increased risk of HEV exposure. (DROPDOWN)** | a. Professional contact with animals  b. Recreational contact with animals  c. Intravenous drug users  d. Rural residents |  |
| **9. Does this study simultaneously sample animals, food, water or other possible local sources of HEV exposure?** | a. Yes, samples animals for HEV exposure (Ig’s)  b. Yes. Samples animals for HEV detection (RT_PCR)  c. Yes, studies genomic evidence comparing animal and human-derived HEV isolates  d. Yes. Other- please specify.  e. No. Only human evidence is investigated in this study. |  |
| **Were co-infections (i.e. HEV and some other microbe) investigated?** | a. Yes, please specify.__________  b. No, only HEV was assayed. |  |
| **QUALITY ASSESSMENT** |  |  |
| **10. Are raw/unadjusted data or measures of association/effect provided?**  **Please choose all that apply.** | a. Yes- prevalence estimates for at least one group can be extracted.  b. Yes, measures of association (or 2x2 table data) related to demographic attributes can be extracted.  **c**. No unadjusted data or measures of effect reported. (EXCLUDE) | Minimum necessary data:   1. *Prevalence/frequency*:   Following data must be reported   - - Numerator ***and*** denominator, ***or***   - proportion + EITHER numerator or denominator   b. Measures of association/effect:   - OR/RR/IR/RD/PAF/ AFe reported ***and*** its measure of variability (SE, SD, CI) *or* P-value is provided ***and*** sample size (n) reported   ***Please note this question is applicable to all study types*** |
|  |  |  |
| **11. Were laboratory methods sufficiently described to allow replication of the study?** | **Please select one option**  a. Yes  b. No  c. Referenced | **a. Yes:**  Minimum necessary laboratory protocol data reported:  ELISA – manufacturer; kit name  **b. No:**   - Methods are not sufficiently reported - Study cannot be reproduced without contacting the author |
| **12. Were samples stored appropriately AND processed/tested within a reasonable period of time after collection?** | a. Yes b. No  c. Not reported. | **Yes**– samples for viral assay must be kept refrigerated at ~ 4C, up to 6 days allowable. Serum samples, if stored for long periods, must be kept frozen – 6-12 months if stored at -20C, indefinitely if stored at -70C. **No** – it is obvious that samples have not been stored properly **Not reported** – from what I have seen, this will apply to most of the articles. Use of this option will allow these articles to pass; the impact of including/excluding these studies will be dealt with in analysis. |
| **13. Does the study report validation of the representativeness of the sample population with the target population?** | a. Yes. Please specify___________  b. No  c. Not applicable | **Yes:** researchers report investigation of one or more parameters (e.g. age structure) to verify representativeness or similarity between sample and target populations. **No:** no such investigation is reported. **Not applicable**: Findings were not intended to be generalized beyond study group. |
| **14. How were organizations (clinics etc.), sampling sites, or other types of groups of subjects selected to participate in this study?** | a. Whole registry b. Random c. Reported random d. Systematic e. Convenience f. Purposive  g. Not applicable | **Whole registry:** Organizations were chosen through a registry (such as region or disease). **Random:** Computer or random numbers table, a priori, stratified random sample, cluster random sample. **Reported random:** Author indicates random, but randomization is not explained. **Systematic:** Taking n samples at interval of x. **Convenience: Organisations** were identified by personal affiliations, easy accessibility, were the places that agreed to participate, or it was not described in the paper.  **Not applicable:** Organizations/groups were not selected to participate in this study. |
| **15. How were individual subjects selected to participate in this study?** | a. Whole registry b. Random c. Reported random d. Systematic e. Convenience f. Purposive | **Random:** Computer or random numbers table, a priori, stratified random sample, cluster random sample. **Reported random:** Author indicates random, but randomization is not explained. **Systematic:** Taking n samples at interval of x. **Convenience:** Subjects were identified by personal contacts or responded to a survey, or it was not described in the paper. |
| **16. Selective reporting**  *Did the authors report all intended outcomes?* | a. Low  b. High  c. Unclear | **Low:** there is no evidence that outcomes were selectively reported (e.g. all relevant outcomes in the methods section are reported in the results section).  **High:** some important outcomes are omitted from the results.  **Unclear:** insufficient information provided to permit judgement. |
| **17. Other**  *Was the study free of other problems that could put it at a high risk of bias?* | a. Low  b. High  c. Unclear | State any important concerns about bias not addressed in the other domains in the tool (e.g. potential for subjects’ ethnicity to affect results)  **Low:** there is no risk of other biases.  **High:** there is a risk of other biases (please specify details).  **Unclear:** possible risk of other biases but insufficient information provided to permit judgement (please specify details). |
| **18. Overall risk-of-bias for each outcome (within-study summary assessment)** | a. Low ______  b. High ______  c. Unclear _______  Separate risk for different outcomes, specify: | **Low:** plausible bias unlikely to seriously alter the results. Low risk of bias for key domains.  **High:** plausible bias that seriously weakens confidence in the results. High risk of bias for key domains.  **Unclear:** plausible bias that raises some doubt about the results. Unclear risk of bias for key domains.  ***Assessments should be made for each main outcome (or class of outcomes), as appropriate.*** *If more than one answer please specify which outcomes are associated with each answer.* |

**HEV_sero_prevalence _RF_DE_20mar**

Notes: separate form to be completed for each individual line of data in Excel

| **QUESTIONS** | **OPTIONS** | **NOTES** |
| --- | --- | --- |
| **1. What is the assay used for the following data?** | a. Wantai IgG  b. Wantai IgM  C. Wantai IgG/IgM  Etc- this is a dynamic question so we can fill in more options as we go | Key question. Parent Form is DCU/QA form |
| **2. What is the name used by the authors to describe this assay?** | a. ________________________  b. Not reported | Please cut-and-paste the term the authors use. There is more than one Wantai IgG kit, for example. However I am not confident that this data will be consistently reported.  If the authors do not report the specific trade name of the kit, please indicate ‘Not reported’ |
| **2. In what country doe s the following sampling take place?** | - Andorra, - Argentina, - Australia, - Austria, - Bahrain, - Belgium, - Brunei, - Canada, - Chile, - Croatia, - Cyprus, - Czech Republic, - Denmark, - Estonia - Finland, - France, - Germany, - Greece, - Hong Kong, - Hungary, - Iceland, - Ireland, - Israel, - Italy, - Japan, - South Korea (aka Republic of Korea), - Kuwait, - Latvia, - Liechtenstein, - Lithuania, - Luxembourg, - Malta, - Montenegro, - Netherlands, - New Zealand, - Norway, - Poland, - Portugal, - Qatar, - Saudi Arabia, - Slovakia, - Slovenia, - Spain, - Sweden, - Switzerland, - United Arab Emirates, - United Kingdom, - United States. - **ONLY Not Relevant Country (exclude): _____** |  |
| **3. In what region of the above country does sampling take place** | a. ___________  b. Not applicable | b. Most authors will not specify region; there are a couple of EU countries with well defined differences across regions however |
| **4. During what chronological time period does sampling take place?** | Month-year to month-year |  |
| **5. What is the population sampled? Please check all that apply.** | a. Blood donor survey  b. General population survey  c. Targeted patient group(s).  d. Pregnant women  e. Armed forces  f. Increased risk of HEV exposure  g. (Add additional categories as needed) | a. Survey of samples derived from individuals volunteering blood donation  b. Survey is intended to be representative of the study location’s general population  c. examples could include: liver/cell or organ recipient, HIV patients  f. e.g. farmers, veterinarians |
| **Targeted patient groups. Please check all that apply. (DROPDOWN)** | **a. Liver patients**  b. Cancer patients  c. Transplant patients  d. Hemodialysis patients  e. Frequent blood or blood product recipients  f. HIV positive patients  e. Other. Please specify. | **a. Not relevant. Please do not extract data pertaining to liver patients.** |
| **Increased risk of HEV exposure. (DROPDOWN)** | a. Occupational contact with animals  b. Recreational contact with animals  c. Intravenous drug users  d. Rural residents |  |
| **6. Please list any additional specific descriptors used by the authors to describe the population to whom this data pertains.** | Text ______________________. | e.g. if #5 category were ‘occupational animal contact’; here authors might specify butchers |
| **7. What is the stratum of ages of subjects sampled, to which this line of data pertains?** | a. Not specified  b. Children  c. __________ |  |
| **8. Please describe the age range of the population sampled.** | Text ______________________ |  |
| **9. Were co-infections with other microbes measured?** | a. Yes. HIV  b. Yes. Hepatitis C virus (HCV)  c. Yes. Other. Please specify  d. No. No other infections were measured. |  |
| **10. Please record the prevalence data pertaining to the population described in the previous questions.** | \| Num pos \| Num total \| 95% CI  lower \| 95% CI upper \| SE \| \| --- \| --- \| --- \| --- \| --- \| \|  \|  \|  \|  \|  \| |  |
| **11. Please record the association data pertaining to the population described in the previous questions.** | \| Exp  Num pos \| Exp Num total \| Non  Num  pos \| Non  Num  tot \| Measure  Assoc., Magnitude \| 95% CI  low \| 95%  CI  up \| SE \| \| --- \| --- \| --- \| --- \| --- \| --- \| --- \| --- \| \|  \|  \|  \|  \|  \|  \|  \|  \| |  |
| **12. Please describe the interpretation of the measure of association reported in Q11.** | a. _____________________ | E.g. Veterinarians have 3.0 times greater odds of HEV IgG sero-positivity relative to the general population |
