# Supplementary material for: Systematic review and meta-analysis of the seroprevalence of hepatitis E virus in the general population across non-endemic countries
Source: PLoS One. 2019 Jun 7;14(6):e0216826. doi: 10.1371/journal.pone.0216826 (PMC6555507; doi:10.1371/journal.pone.0216826)
Supplement: S3 File — (DOCX) [file pone.0216826.s005.docx]

# S3 File. Relevant study locations

**Hepatitis E virus seroprevalence review: geographic inclusion and exclusion criteria**

**1. Included study locations**

The review outcome of interest is human exposure to HEV genotypes which are potentially zoonotic, as measured by sero-prevalence of HEV IgG or IgM antibodies. Therefore, sampling locations of interest are those in which exposure is unlikely to result from consumption of HEV-contaminated water, AND in which evidence exists that common locally circulating human HEV strains are potentially zoonotic (e.g. genotypes 3 or 4, or camel –associated/gt 7), and NOT genotypes 1 or 2 (considered not to be potentially zoonotic, and also largely associated with waterborne outbreaks).

To identify study locations in which human consumption of HEV contaminated water is unlikely, the review will use the United Nations (UN) categorization of a country having ‘Very High human development’ via the UN human development index, as a proxy indicator for the presence of local public health infrastructure sufficient to prevent transmission of microbial disease via drinking water. [Background: The UN human development index for a specific country is composed of national mean life expectancy at birth, expected years of schooling, mean years of schooling, and gross national income per capita.]

**Methods**: All of the countries categorized as ‘very high human development ’ by the UN were identified (United Nations Development Programme, 2015). Evidence to support the assertion that potentially zoonotic genotypes (3 or 4, or camelid-associated/gt7) are common circulating strains in humans in these countries was investigated, using:

i. Search of citations captured by this review.

ii. Petrik et al. (2016), which described the findings regarding the current national status of human HEV infection in selected countries, derived from an international panel of experts in the field of blood collection and transfusion.

iii. Pubmed and Google search for research originating from the country of interest (which captured a couple of relevant studies not identified by our initial electronic search; most are only available in foreign languages other than French or Spanish, however).

iv. Genbank search for HEV sequences from the country of interest, since not all relevant work is published, but some relevant sequences are identified by country and species of origin, in Genbank.

**Comments**: Evidence that potentially zoonotic HEV genotypes are circulating in humans, was easily obtained for most, but not all, of the countries categorized as ‘very high human development’. However, for a small number of countries (n = 6), although HEV sero-prevalence surveys are reported in a variety of healthy human populations captured by this review, HEV RNA detection from these countries is only reported in animals, mostly pigs. In two countries (Iceland and Saudi Arabia), although HEV sero-prevalence is surveyed in healthy human populations, currently no local HEV sequences have been reported from any animal or human population, either in scientific literature or in the GenBank, to allow genotyping.

The evidence that potentially zoonotic HEV genotypes are circulating in humans in a given ‘very high’ country varies, therefore from:

i. strong – large population-based sero-surveys have been done with genotyping of HEV isolates recovered, as well as genotyping of HEV isolates infecting clinical cases. Consensus of experts regarding the genotype of predominate strains has been presented in reviews/international forum. Example of a country with this level of evidence: Canada.

ii. intermediate- countries with similar public health status (and close geographically; HEV strains tend to be related spatially) to countries having strong evidence, as in (i), and the presence of HEV infection has been reported in local animal populations. E.g. Croatia – close to Italy, where evidence is strong; HEV detected in Croatian pigs.

iii. weak- No sequencing/genotyping of local HEV isolates has been performed but presence of zoonotic strains seems more plausible than genotypes 1 or 2 given public health standards of the country (precluding waterborne outbreaks) and ethnicity of population (not predominately from regions where genotype 1 is common). E.g. Iceland

The Gulf states which are categorized ‘very high’ by the UN, are different from countries in the Americas or Europe similarly categorized, for a couple of reasons:

- Much of the workforce (and ergo the healthy population) are foreign workers. Studies of subjects who are purposively selected because they are foreign/refugees/recent immigrants will be excluded, for all countries, not just the Gulf states (and the review has captured some). However, the origin of the individual subjects may be tough to determine from some blood donor surveys. General impression presented by authors is that many foreign workers employed in the Gulf states are originally from countries where genotype 1 HEV infection is considered widespread (e.g. India, Pakistan, Bangladesh).
- Recent discovery of camel HEV and report of locally acquired case of Hepatitis E associated with camel consumption, in United Arab Emirates, suggests another potential zoonotic exposure in this region.

**Propose:** All ‘very high’ countries are included in the review, including the Gulf states thus categorized. The varying levels of evidence regarding the genotype of local HEV strains will be a point for discussion or further analysis.

**Table 1. Study locations included in the HEV sero-prevalence review.**

**Countries contributing sero-prevalence surveys to this review, with lower levels of evidence for genotype of circulating HEV strains, are indicated in red**

| UN category ‘Very High Human Development’ | Citations from this country captured in HEV sero search? | Potentially zoonotic genotypes (3, 4, or 7/camel-associated) detected in this country, reported in Google/Pubmed/Genbank? | References supporting potentially zoonotic genotype detection |
| --- | --- | --- | --- |
| Andorra | No | No | N/A |
| Argentina | Yes (RS2)- healthy population surveys | Yes - human | Petrik et al., 2016 |
| Australia | Yes (RS2) - healthy population surveys | Yes - human | Petrik et al., 2016 |
| Austria | Yes (RS2) - healthy population surveys | Yes-human | Fischer et al., 2015 |
| Bahrain | No | No | N/A |
| Belgium | Yes (RS2) - healthy population surveys | Yes-human | Thiry et al., 2014* |
| Brunei | No | No | N/A |
| Canada | Yes (RS2) - healthy population surveys | Yes-human | Petrik et al., 2016 |
| Chile | Yes (RS2) - healthy population surveys | Yes-HEV detection pigs | Ibarra et al., 2007 |
| Croatia | Yes (RS2) - healthy population surveys | Yes- HEV detection pigs | Prpic et al., 2015* |
| Cyprus | Yes RS(2) - healthy population surveys | No | N/A |
| Czech Republic | Yes RS(2) - healthy population surveys | Yes | Vasickova et al. (refid 9456) |
| Denmark | Yes RS(2) - healthy population surveys | Yes | Midgley et al., 2014 |
| Finland | No | Yes- single case report in Finnish | Kettunen et al., 2013* |
| France | Yes RS(2) - healthy population surveys | Yes | Petrik et al., 2016 |
| Germany | Yes RS(2) - healthy population surveys | Yes | Petrik et al., 2016 |
| Greece | Yes RS(2) - healthy population surveys | Yes – HEV detection pigs | Siochu et al., 2009 |
| Hong Kong | Yes RS(2) - healthy population surveys | Yes | Tai et al., 2009 |
| Hungary | Yes RS(2) - healthy population surveys | Yes | Reuter et al., 2009 |
| Iceland | Yes (RS2) - healthy population surveys | No | N/A |
| Ireland | Yes RS(2) - healthy population surveys | Yes | GenBank: KT873493.1, unpublished Keane et al., refid 9000 |
| Israel | Yes RS(2) - healthy population surveys | Yes | Ram et al., 2016 |
| Italy | Yes RS(2) - healthy population surveys | Yes | Petrik et al., 2016 |
| Japan | Yes RS(2) - healthy population surveys | Yes | Petrik et al., 2016 |
| Korea (South) | Yes RS(2) - healthy population surveys | Yes | Petrik et al., 2016 |
| Kuwait | Yes RS(2) liver patients | No | N/A |
| Latvia | No | No | N/A |
| Liechtenstein | No | No | N/A |
| Lithuania | No | No | N/A |
| Luxembourg | No | No | N/A |
| Malta | No | No | N/A |
| Montenegro | Yes RS(2)- hepatitis patients | No | No |
| Netherlands | Yes RS(2) - healthy population surveys | Yes | Petrik et al, 2016 |
| New Zealand | Yes RS(2) - healthy population surveys | Yes | Dalton et al., 2007 |
| Norway | Yes RS(2) - healthy population surveys | Yes – HEV seroprevalence pigs | Lange et al., 2017 |
| Poland | Yes RS(2) - healthy population surveys *Sadowska-Todys et al., 2015 | Yes – HEV detection pigs | Bura et al., 2015*– published in Polish |
| Portugal | Yes RS(2) - healthy population surveys | Yes | Breda et al., 2014* Genbank KX073466.1, KX073465.1 |
| Qatar | Yes RS(2) migrant workers | No | N/A |
| Saudi Arabia | Yes RS(2) - healthy population surveys | No | N/A |
| Slovakia | No | No | N/A |
| Slovenia | No | No | N/A |
| Spain | Yes RS(2) - healthy population surveys | Yes | Petrik et al., 2016 |
| Sweden | Yes RS(2) - healthy population surveys | Yes | Petrik et al., 2016 |
| Switzerland | Yes RS(2) - healthy population surveys | Yes | Doerig et al., refid 9242 |
| United Arab Emirates | Yes RS(2) - healthy population surveys | Yes - camels | Lee et al., 2015 |
| United Kingdom | Yes RS(2) - healthy population surveys | Yes | Petrik et al., 2016 |
| United States | Yes RS(2) - healthy population surveys | Yes | Petrik et al., 2016 |

* indicates citation captured after initial electronic search

References

Breda, F., Cochicho, J., Mesquita, J.R., Bento, A., Oliveira, R.P., Louro, E., Nascimento, M.S., Leitao, J., Gomes, H.V., Carvalho, A. 2014. First report of chronic hepatitis E in renal transplant recipients in Portugal. J. Infect Dev. Ctries 8, 1639-1642.

[Bura, M](https://www.ncbi.nlm.nih.gov/pubmed/?term=Bura%20M%5BAuthor%5D&cauthor=true&cauthor_uid=25748623)., [Michalak, M](https://www.ncbi.nlm.nih.gov/pubmed/?term=Michalak%20M%5BAuthor%5D&cauthor=true&cauthor_uid=25748623)., [Chojnicki, M](https://www.ncbi.nlm.nih.gov/pubmed/?term=Chojnicki%20M%5BAuthor%5D&cauthor=true&cauthor_uid=25748623)., [Czajka, A](https://www.ncbi.nlm.nih.gov/pubmed/?term=Czajka%20A%5BAuthor%5D&cauthor=true&cauthor_uid=25748623)., [Kowala-Piaskowska, A](https://www.ncbi.nlm.nih.gov/pubmed/?term=Kowala-Piaskowska%20A%5BAuthor%5D&cauthor=true&cauthor_uid=25748623)., [Mozer-Lisewska, I](https://www.ncbi.nlm.nih.gov/pubmed/?term=Mozer-Lisewska%20I%5BAuthor%5D&cauthor=true&cauthor_uid=25748623). 2015. Seroprevalence of anti-HEV IgG in 182 Polish patients. Post. Epy. Hig. Med. Dosw. 8, 320-632. doi: 10.5604/17322693.1143051.

[Ibarra, V.H](https://www.ncbi.nlm.nih.gov/pubmed/?term=Ibarra%20V%20H%5BAuthor%5D&cauthor=true&cauthor_uid=17989856)., [Riedemann, G.S](https://www.ncbi.nlm.nih.gov/pubmed/?term=Riedemann%20G%20S%5BAuthor%5D&cauthor=true&cauthor_uid=17989856)., [Reinhardt, V.G](https://www.ncbi.nlm.nih.gov/pubmed/?term=Reinhardt%20V%20G%5BAuthor%5D&cauthor=true&cauthor_uid=17989856)., [Calvo, A.M](https://www.ncbi.nlm.nih.gov/pubmed/?term=Calvo%20A%20M%5BAuthor%5D&cauthor=true&cauthor_uid=17989856). 2007. Presence of anti-hepatitis E virus antibodies in swine: is it an animal reservoir for hepatitis E?. Rev. Med. Chil. 135, 997-1001. Epub 2007 Oct 25.

[Lange, H](https://www.ncbi.nlm.nih.gov/pubmed/?term=Lange%20H%5BAuthor%5D&cauthor=true&cauthor_uid=27671461)., [Øverbø, J](https://www.ncbi.nlm.nih.gov/pubmed/?term=%C3%98verb%C3%B8%20J%5BAuthor%5D&cauthor=true&cauthor_uid=27671461)., [Borgen, K](https://www.ncbi.nlm.nih.gov/pubmed/?term=Borgen%20K%5BAuthor%5D&cauthor=true&cauthor_uid=27671461)., [Dudman, S](https://www.ncbi.nlm.nih.gov/pubmed/?term=Dudman%20S%5BAuthor%5D&cauthor=true&cauthor_uid=27671461)., [Hoddevik, G](https://www.ncbi.nlm.nih.gov/pubmed/?term=Hoddevik%20G%5BAuthor%5D&cauthor=true&cauthor_uid=27671461)., [Urdahl, A.M](https://www.ncbi.nlm.nih.gov/pubmed/?term=Urdahl%20AM%5BAuthor%5D&cauthor=true&cauthor_uid=27671461)., [Vold, L](https://www.ncbi.nlm.nih.gov/pubmed/?term=Vold%20L%5BAuthor%5D&cauthor=true&cauthor_uid=27671461)., [Sjurseth, S.K](https://www.ncbi.nlm.nih.gov/pubmed/?term=Sjurseth%20SK%5BAuthor%5D&cauthor=true&cauthor_uid=27671461). 2017. Hepatitis E in Norway: seroprevalence in humans and swine. Epidemiol. Infect. 145, 181-186. Epub 2016 Sep 27.

Lee, G.H., Tan, B.H., Teo, E.C., Lim, S.G., Dan, Y.Y., Wee, A., Aw, P.P., Zhu, Y., Hibberd, M.L., Tan, C.K., Purdy, M.A., Teo, C.G. 2015. [Chronic infection with camelid hepatitis E virus in a liver transplant recipient who regularly consumes camel meat and milk.](https://www.ncbi.nlm.nih.gov/pubmed/26551551) Gastroenterology. 150, 355-357.e3. doi: 10.1053/j.gastro.2015.10.048.

[Petrik, J](https://www.ncbi.nlm.nih.gov/pubmed/?term=Petrik%20J%5BAuthor%5D&cauthor=true&cauthor_uid=26198159)., [Lozano, M](https://www.ncbi.nlm.nih.gov/pubmed/?term=Lozano%20M%5BAuthor%5D&cauthor=true&cauthor_uid=26198159)., [Seed, C.R](https://www.ncbi.nlm.nih.gov/pubmed/?term=Seed%20CR%5BAuthor%5D&cauthor=true&cauthor_uid=26198159)., [Faddy, H.M](https://www.ncbi.nlm.nih.gov/pubmed/?term=Faddy%20HM%5BAuthor%5D&cauthor=true&cauthor_uid=26198159)., [Keller, A.J](https://www.ncbi.nlm.nih.gov/pubmed/?term=Keller%20AJ%5BAuthor%5D&cauthor=true&cauthor_uid=26198159)., [Prado Scuracchio, P.S](https://www.ncbi.nlm.nih.gov/pubmed/?term=Prado%20Scuracchio%20PS%5BAuthor%5D&cauthor=true&cauthor_uid=26198159)., [Wendel, S](https://www.ncbi.nlm.nih.gov/pubmed/?term=Wendel%20S%5BAuthor%5D&cauthor=true&cauthor_uid=26198159)., [Andonov, A](https://www.ncbi.nlm.nih.gov/pubmed/?term=Andonov%20A%5BAuthor%5D&cauthor=true&cauthor_uid=26198159)., [Fearon, M](https://www.ncbi.nlm.nih.gov/pubmed/?term=Fearon%20M%5BAuthor%5D&cauthor=true&cauthor_uid=26198159)., [Delage, G](https://www.ncbi.nlm.nih.gov/pubmed/?term=Delage%20G%5BAuthor%5D&cauthor=true&cauthor_uid=26198159)., [Zhang, J](https://www.ncbi.nlm.nih.gov/pubmed/?term=Zhang%20J%5BAuthor%5D&cauthor=true&cauthor_uid=26198159)., [Shih, J.W](https://www.ncbi.nlm.nih.gov/pubmed/?term=Shih%20JW%5BAuthor%5D&cauthor=true&cauthor_uid=26198159)., [Gallian, P](https://www.ncbi.nlm.nih.gov/pubmed/?term=Gallian%20P%5BAuthor%5D&cauthor=true&cauthor_uid=26198159)., [Djoudi, R](https://www.ncbi.nlm.nih.gov/pubmed/?term=Djoudi%20R%5BAuthor%5D&cauthor=true&cauthor_uid=26198159)., [Tiberghien, P](https://www.ncbi.nlm.nih.gov/pubmed/?term=Tiberghien%20P%5BAuthor%5D&cauthor=true&cauthor_uid=26198159)., [Izopet, J](https://www.ncbi.nlm.nih.gov/pubmed/?term=Izopet%20J%5BAuthor%5D&cauthor=true&cauthor_uid=26198159)., [Dreier, J](https://www.ncbi.nlm.nih.gov/pubmed/?term=Dreier%20J%5BAuthor%5D&cauthor=true&cauthor_uid=26198159)., [Vollmer, T](https://www.ncbi.nlm.nih.gov/pubmed/?term=Vollmer%20T%5BAuthor%5D&cauthor=true&cauthor_uid=26198159)., [Knabbe, C](https://www.ncbi.nlm.nih.gov/pubmed/?term=Knabbe%20C%5BAuthor%5D&cauthor=true&cauthor_uid=26198159)., [Aggarwal, R](https://www.ncbi.nlm.nih.gov/pubmed/?term=Aggarwal%20R%5BAuthor%5D&cauthor=true&cauthor_uid=26198159)., [Goel , A](https://www.ncbi.nlm.nih.gov/pubmed/?term=Goel%20A%5BAuthor%5D&cauthor=true&cauthor_uid=26198159)., [Ciccaglione, A.R](https://www.ncbi.nlm.nih.gov/pubmed/?term=Ciccaglione%20AR%5BAuthor%5D&cauthor=true&cauthor_uid=26198159)., [Matsubayashi, K](https://www.ncbi.nlm.nih.gov/pubmed/?term=Matsubayashi%20K%5BAuthor%5D&cauthor=true&cauthor_uid=26198159)., [Satake, M](https://www.ncbi.nlm.nih.gov/pubmed/?term=Satake%20M%5BAuthor%5D&cauthor=true&cauthor_uid=26198159)., [Tadokoro, K](https://www.ncbi.nlm.nih.gov/pubmed/?term=Tadokoro%20K%5BAuthor%5D&cauthor=true&cauthor_uid=26198159)., [Jeong, S.H](https://www.ncbi.nlm.nih.gov/pubmed/?term=Jeong%20SH%5BAuthor%5D&cauthor=true&cauthor_uid=26198159)., [Zaaijer, H.L](https://www.ncbi.nlm.nih.gov/pubmed/?term=Zaaijer%20HL%5BAuthor%5D&cauthor=true&cauthor_uid=26198159)., [Zhiburt, E](https://www.ncbi.nlm.nih.gov/pubmed/?term=Zhiburt%20E%5BAuthor%5D&cauthor=true&cauthor_uid=26198159)., [Chay, J](https://www.ncbi.nlm.nih.gov/pubmed/?term=Chay%20J%5BAuthor%5D&cauthor=true&cauthor_uid=26198159)., [Teo, D](https://www.ncbi.nlm.nih.gov/pubmed/?term=Teo%20D%5BAuthor%5D&cauthor=true&cauthor_uid=26198159)., [Chua, S.S](https://www.ncbi.nlm.nih.gov/pubmed/?term=Chua%20SS%5BAuthor%5D&cauthor=true&cauthor_uid=26198159)., [Piron, M](https://www.ncbi.nlm.nih.gov/pubmed/?term=Piron%20M%5BAuthor%5D&cauthor=true&cauthor_uid=26198159)., [Sauleda, S](https://www.ncbi.nlm.nih.gov/pubmed/?term=Sauleda%20S%5BAuthor%5D&cauthor=true&cauthor_uid=26198159)., [Echevarría, J.M](https://www.ncbi.nlm.nih.gov/pubmed/?term=Echevarr%C3%ADa%20JM%5BAuthor%5D&cauthor=true&cauthor_uid=26198159)., [Dalton, H](https://www.ncbi.nlm.nih.gov/pubmed/?term=Dalton%20H%5BAuthor%5D&cauthor=true&cauthor_uid=26198159)., [Stramer, S.L](https://www.ncbi.nlm.nih.gov/pubmed/?term=Stramer%20SL%5BAuthor%5D&cauthor=true&cauthor_uid=26198159). 2016. Hepatitis E. Vox. Sang. 110, 93-130. doi: 10.1111/vox.12285. Epub 2015 Jul 21.

Prpić, J., Černi, S., Škorić, D., Keros, T., Brnić, D., Cvetnić, Ž., Jemeršić, L. 2015. [Distribution and molecular characterization of hepatitis E virus in domestic animals and wildlife in Croatia.](https://www.ncbi.nlm.nih.gov/pubmed/25820413) Food Environ. Virol. 7, 195-205. doi: 10.1007/s12560-015-9193-5.

[Ram, D](https://www.ncbi.nlm.nih.gov/pubmed/?term=Ram%20D%5BAuthor%5D&cauthor=true&cauthor_uid=27246446)., [Manor, Y](https://www.ncbi.nlm.nih.gov/pubmed/?term=Manor%20Y%5BAuthor%5D&cauthor=true&cauthor_uid=27246446)., [Gozlan, Y](https://www.ncbi.nlm.nih.gov/pubmed/?term=Gozlan%20Y%5BAuthor%5D&cauthor=true&cauthor_uid=27246446)., [Schwartz, E](https://www.ncbi.nlm.nih.gov/pubmed/?term=Schwartz%20E%5BAuthor%5D&cauthor=true&cauthor_uid=27246446)., [Ben-Ari, Z](https://www.ncbi.nlm.nih.gov/pubmed/?term=Ben-Ari%20Z%5BAuthor%5D&cauthor=true&cauthor_uid=27246446)., [Mendelson, E](https://www.ncbi.nlm.nih.gov/pubmed/?term=Mendelson%20E%5BAuthor%5D&cauthor=true&cauthor_uid=27246446)., [Mor, O](https://www.ncbi.nlm.nih.gov/pubmed/?term=Mor%20O%5BAuthor%5D&cauthor=true&cauthor_uid=27246446). 2016. Hepatitis E Virus genotype 3 in sewage and genotype 1 in acute hepatitis cases, Israel. Am. J. Hygiene Trop. Med. 95, 216-220. doi: 10.4269/ajtmh.15-0925. Epub 2016 May 31.

[Reuter, G](https://www.ncbi.nlm.nih.gov/pubmed/?term=Reuter%20G%5BAuthor%5D&cauthor=true&cauthor_uid=19217346)., [Fodor, D](https://www.ncbi.nlm.nih.gov/pubmed/?term=Fodor%20D%5BAuthor%5D&cauthor=true&cauthor_uid=19217346)., [Forgách, P](https://www.ncbi.nlm.nih.gov/pubmed/?term=Forg%C3%A1ch%20P%5BAuthor%5D&cauthor=true&cauthor_uid=19217346)., [Kátai, A](https://www.ncbi.nlm.nih.gov/pubmed/?term=K%C3%A1tai%20A%5BAuthor%5D&cauthor=true&cauthor_uid=19217346)., [Szucs, G](https://www.ncbi.nlm.nih.gov/pubmed/?term=Szucs%20G%5BAuthor%5D&cauthor=true&cauthor_uid=19217346). 2009. Characterization and zoonotic potential of endemic hepatitis E virus (HEV) strains in humans and animals in Hungary. [J. Clin. Virol.](https://www.ncbi.nlm.nih.gov/pubmed/?term=Characterization+and+zoonotic+potential+of+endemic+hepatitis+E+virus+%28HEV%29+strains+in+humans+and+animals+in+Hungary+G.+Reuter%2C+D.+Fodor%2C+P.+Forgach%2C+A.+Katai%2C+G.+Szucs) 44, 277-281. doi: 10.1016/j.jcv.2009.01.008. Epub 2009 Feb 12.

[Sadkowska-Todys, M](https://www.ncbi.nlm.nih.gov/pubmed/?term=Sadkowska-Todys%20M%5BAuthor%5D&cauthor=true&cauthor_uid=27139343)., [Baumann-Popczyk, A](https://www.ncbi.nlm.nih.gov/pubmed/?term=Baumann-Popczyk%20A%5BAuthor%5D&cauthor=true&cauthor_uid=27139343)., [Wnukowska, N](https://www.ncbi.nlm.nih.gov/pubmed/?term=Wnukowska%20N%5BAuthor%5D&cauthor=true&cauthor_uid=27139343)., [Popczyk, B](https://www.ncbi.nlm.nih.gov/pubmed/?term=Popczyk%20B%5BAuthor%5D&cauthor=true&cauthor_uid=27139343)., [Kucharczyk, B](https://www.ncbi.nlm.nih.gov/pubmed/?term=Kucharczyk%20B%5BAuthor%5D&cauthor=true&cauthor_uid=27139343)., [Gołąb, E](https://www.ncbi.nlm.nih.gov/pubmed/?term=Go%C5%82%C4%85b%20E%5BAuthor%5D&cauthor=true&cauthor_uid=27139343). 2015. Occurrence and prevalence of selected zoonotic agents: *Echinococcus multilocularis*, *Trichinella spiralis* and hepatitis E virus (HEV) in the population of Polish hunters--results of the study conducted in 2010-2012. Przegl. Epidemiol. 69, 673-678, 823-827.

Siochu, A., [Tzika, E](javascript:__doLinkPostBack('','ss~~AR%20%22Tzika%2C%20Elena%22%7C%7Csl~~rl','');)., [Alexopoulos, C.](javascript:__doLinkPostBack('','ss~~AR%20%22Alexopoulos%2C%20C.%22%7C%7Csl~~rl','');) , [Kyriakis, S.C.](javascript:__doLinkPostBack('','ss~~AR%20%22Kyriakis%2C%20S.%20C.%22%7C%7Csl~~rl','');), [Froesner, G.](javascript:__doLinkPostBack('','ss~~AR%20%22Froesner%2C%20G.%22%7C%7Csl~~rl','');) 2009. First report of serological evidence of hepatitis E virus infection in swine in northern Greece. [Acta Veterinaria](javascript:__doLinkPostBack('','mdb~~a9h%7C%7Cjdb~~a9hjnh%7C%7Css~~JN%20%22Acta%20Veterinaria%22%7C%7Csl~~jh','');). 59, 205-211.

[Tai, A.L](https://www.ncbi.nlm.nih.gov/pubmed/?term=Tai%20AL%5BAuthor%5D&cauthor=true&cauthor_uid=19382265)., [Cheng, P.K](https://www.ncbi.nlm.nih.gov/pubmed/?term=Cheng%20PK%5BAuthor%5D&cauthor=true&cauthor_uid=19382265)., [Ip, S.M](https://www.ncbi.nlm.nih.gov/pubmed/?term=Ip%20SM%5BAuthor%5D&cauthor=true&cauthor_uid=19382265)., [Wong, R.M](https://www.ncbi.nlm.nih.gov/pubmed/?term=Wong%20RM%5BAuthor%5D&cauthor=true&cauthor_uid=19382265)., [Lim, W.W](https://www.ncbi.nlm.nih.gov/pubmed/?term=Lim%20WW%5BAuthor%5D&cauthor=true&cauthor_uid=19382265). 2009. Molecular epidemiology of hepatitis E virus in Hong Kong. [J. Med. Virol.](https://www.ncbi.nlm.nih.gov/pubmed/?term=Molecular+epidemiology+of+hepatitis+E+virus+in+Hong+Kong+A.+L.+Tai%2C+P.+K.+Cheng%2C+S.+M.+Ip%2C+R.+M.+Wong%2C+W.+W.+Lim) 81, 1062-1068. doi: 10.1002/jmv.21497.

United Nations Development Programme. 2015. Human Development Report 2015: Work for human development. Retrieved from: [http://hdr.undp.org/sites/default/files/2015_human_development_report.pdf Accessed 13 January 2017](http://hdr.undp.org/sites/default/files/2015_human_development_report.pdf%20%20%20%20Accessed%2013%20January%202017).

**2. Excluded study locations**.

Table 2 contains a list of countries NOT categorized as ‘Very high human development” by the UN development programme.

In general, looking at the map presenting these data (<http://hdr.undp.org/en/countries>) there seems to be strong relationship between UN categorization and circulating HEV genotypes, with ‘low’ and ‘medium’ countries being associated with genotypes 1 or 2.

The ‘high’ category is a little more heterogeneous: several of the countries within this category have reported multiple investigations of HEV sero-prevalence in healthy humans, and also have evidence suggesting that potentially zoonotic genotypes are predominate. These countries are presented in red font and are all categorized as ‘High human development ‘ i.e., they are one category below the one required for inclusion to the review (UN categories include very high–high-medium-low).

Following is a list of countries NOT categorized by the United Nations as having ‘Very high human development’. Columns are only completed for selected countries.

| **Country** | **UN Categorization** | **Relevant studies captured?** | **Genotypes 3 or 4 reported?** | **References** |
| --- | --- | --- | --- | --- |
| Afghanistan |  |  |  |  |
| Albania |  |  |  |  |
| Algeria |  |  |  |  |
| Angola |  |  |  |  |
| Armenia |  |  |  |  |
| Azerbaijan |  |  |  |  |
| Bahamas |  |  |  |  |
| Bangladesh |  |  |  |  |
| Barbados |  |  |  |  |
| Belarus |  |  |  |  |
| Belize |  |  |  |  |
| Benin |  |  |  |  |
| Bhutan |  |  |  |  |
| Bolivia |  |  |  |  |
| Bosnia and Herzegovina |  |  |  |  |
| Botswana |  |  |  |  |
| **Brazil** | High | Yes RS(2) | Yes - humans | Petrik et al., 2016 |
| Bulgaria |  |  |  |  |
| Burkino Faso |  |  |  |  |
| Burundi |  |  |  |  |
| Cabo Verde |  |  |  |  |
| Cambodia |  |  |  |  |
| Cameroon |  |  |  |  |
| Central African Republic |  |  |  |  |
| Chad |  |  |  |  |
| **China** | High | Yes- RS (2) | Yes | Petrik et al., 2016 |
| Colombia |  |  |  |  |
| Comoros |  |  |  |  |
| Congo |  |  |  |  |
| Democratic Republic of Congo |  |  |  |  |
| Costa Rica |  |  |  |  |
| Cuba |  |  |  |  |
| Cote D’Ivoire |  |  |  |  |
| Djibouti |  |  |  |  |
| Dominica |  |  |  |  |
| Dominican Republic |  |  |  |  |
| Ecuador |  |  |  |  |
| Egypt |  |  |  |  |
| El Salvador |  |  |  |  |
| Equatorial Guinea |  |  |  |  |
| Eritrea |  |  |  |  |
| Estonia |  |  |  |  |
| Ethiopia |  |  |  |  |
| Fiji |  |  |  |  |
| Gabon |  |  |  |  |
| Gambia |  |  |  |  |
| Georgia |  |  |  |  |
| Ghana |  |  |  |  |
| Grenada |  |  |  |  |
| Guatemala |  |  |  |  |
| Guinea |  |  |  |  |
| Guinea-Bissau |  |  |  |  |
| Guyana |  |  |  |  |
| Haiti |  |  |  |  |
| Honduras |  |  |  |  |
| India |  |  |  |  |
| Indonesia |  |  |  |  |
| Iran |  |  |  |  |
| Iraq |  |  |  |  |
| Jamaica |  |  |  |  |
| Jordan |  |  |  |  |
| Kazakhstan |  |  |  |  |
| Kenya |  |  |  |  |
| Kiribati |  |  |  |  |
| Kuwait |  |  |  |  |
| Kyrgzstan |  |  |  |  |
| Lao People’s Democratic Republic |  |  |  |  |
| Lebanon |  |  |  |  |
| Lesotho |  |  |  |  |
| Liberia |  |  |  |  |
| Libya |  |  |  |  |
| Lichenstein |  |  |  |  |
| Madagascar |  |  |  |  |
| Malawi |  |  |  |  |
| Malaysia |  |  |  |  |
| Maldives |  |  |  |  |
| Mali |  |  |  |  |
| Malta |  |  |  |  |
| Mauritania |  |  |  |  |
| Mauritius |  |  |  |  |
| **Mexico** | High | Yes RS(2) | Yes – in pigs | No human sequences in Genbank |
| Micronesia |  |  |  |  |
| Moldova |  |  |  |  |
| Mongolia |  |  |  |  |
| Morocco |  |  |  |  |
| Mozambique |  |  |  |  |
| Myanmar |  |  |  |  |
| Namibia |  |  |  |  |
| Nepal |  |  |  |  |
| Nicaragua |  |  |  |  |
| Niger |  |  |  |  |
| Nigeria |  |  |  |  |
| Pakistan |  |  |  |  |
| Palau |  |  |  |  |
| Palestine |  |  |  |  |
| Panama |  |  |  |  |
| Papua New Guinea |  |  |  |  |
| Paraguay |  |  |  |  |
| Peru |  |  |  |  |
| Philippines |  |  |  |  |
| **Romania** | High | Yes RS(2) | Yes- in pigs | GenBank KM042904.1; KM058114.1 |
| Russian Federation |  |  |  |  |
| Rwanda |  |  |  |  |
| Saint Kitts and Nevis |  |  |  |  |
| Saint Lucia |  |  |  |  |
| Saint Vincent |  |  |  |  |
| Samoa |  |  |  |  |
| Sao Tome |  |  |  |  |
| Senegal |  |  |  |  |
| **Serbia** | High | Yes RS(2) | Yes | Widen et al., 2010 |
| Seychelles |  |  |  |  |
| Sierra Leone |  |  |  |  |
| Singapore |  |  |  |  |
| Solomon Islands |  |  |  |  |
| South Africa |  |  |  |  |
| South Sudan |  |  |  |  |
| Sri Lanka |  |  |  |  |
| Sudan |  |  |  |  |
| Surinam |  |  |  |  |
| Swaziland |  |  |  |  |
| Syrian Arab Republic |  |  |  |  |
| Tajikistan |  |  |  |  |
| Tanzania |  |  |  |  |
| **Thailand** | High | Yes RS(2) | Yes - humans | Siripanyaphinyo et al., ‎2014 |
| Former Yugoslav Republic of Macedonia |  |  |  |  |
| Timor-Leste |  |  |  |  |
| Togo |  |  |  |  |
| Tonga |  |  |  |  |
| Trinidad and Tobago |  |  |  |  |
| **Tunisia** | High | Yes RS(2) | Yes - humans | Baji-Hamza et al., 2015 |
| **Turkey** | High | Yes RS(2) | No | N/A |
| Turkmenistan |  |  |  |  |
| Uganda |  |  |  |  |
| Ukraine |  |  |  |  |
| United Arab Emirates |  |  |  |  |
| Uruguay |  |  |  |  |
| Uzbekistan |  |  |  |  |
| Vanuatu |  |  |  |  |
| Venezuela |  |  |  |  |
| Viet Nam |  |  |  |  |
| Yemen |  |  |  |  |
| Zambia |  |  |  |  |
| Zimbabwe |  |  |  |  |

References

[Béji-Hamza, A](https://www.ncbi.nlm.nih.gov/pubmed/?term=B%C3%A9ji-Hamza%20A%5BAuthor%5D&cauthor=true&cauthor_uid=25307960)., [Hassine-Zaafrane, M](https://www.ncbi.nlm.nih.gov/pubmed/?term=Hassine-Zaafrane%20M%5BAuthor%5D&cauthor=true&cauthor_uid=25307960)., [Khélifi-Gharbi ,H](https://www.ncbi.nlm.nih.gov/pubmed/?term=Kh%C3%A9lifi-Gharbi%20H%5BAuthor%5D&cauthor=true&cauthor_uid=25307960)., [Della Libera, S](https://www.ncbi.nlm.nih.gov/pubmed/?term=Della%20Libera%20S%5BAuthor%5D&cauthor=true&cauthor_uid=25307960)., [Iaconelli, M](https://www.ncbi.nlm.nih.gov/pubmed/?term=Iaconelli%20M%5BAuthor%5D&cauthor=true&cauthor_uid=25307960)., [Muscillo, M](https://www.ncbi.nlm.nih.gov/pubmed/?term=Muscillo%20M%5BAuthor%5D&cauthor=true&cauthor_uid=25307960)., [Petricca, S](https://www.ncbi.nlm.nih.gov/pubmed/?term=Petricca%20S%5BAuthor%5D&cauthor=true&cauthor_uid=25307960)., [Ciccaglione, A.R](https://www.ncbi.nlm.nih.gov/pubmed/?term=Ciccaglione%20AR%5BAuthor%5D&cauthor=true&cauthor_uid=25307960)., [Bruni, R](https://www.ncbi.nlm.nih.gov/pubmed/?term=Bruni%20R%5BAuthor%5D&cauthor=true&cauthor_uid=25307960)., [Taffon, S](https://www.ncbi.nlm.nih.gov/pubmed/?term=Taffon%20S%5BAuthor%5D&cauthor=true&cauthor_uid=25307960)., [Aouni, M](https://www.ncbi.nlm.nih.gov/pubmed/?term=Aouni%20M%5BAuthor%5D&cauthor=true&cauthor_uid=25307960)., [La Rosa, G](https://www.ncbi.nlm.nih.gov/pubmed/?term=La%20Rosa%20G%5BAuthor%5D&cauthor=true&cauthor_uid=25307960).2015. Hepatitis E virus genotypes 1 and 3 in wastewater samples in Tunisia**.** Arch. Virol. 160, 183-189. doi: 10.1007/s00705-014-2251-8. Epub 2014 Oct 12.

[Petrik, J](https://www.ncbi.nlm.nih.gov/pubmed/?term=Petrik%20J%5BAuthor%5D&cauthor=true&cauthor_uid=26198159)., [Lozano, M](https://www.ncbi.nlm.nih.gov/pubmed/?term=Lozano%20M%5BAuthor%5D&cauthor=true&cauthor_uid=26198159)., [Seed, C.R](https://www.ncbi.nlm.nih.gov/pubmed/?term=Seed%20CR%5BAuthor%5D&cauthor=true&cauthor_uid=26198159)., [Faddy, H.M](https://www.ncbi.nlm.nih.gov/pubmed/?term=Faddy%20HM%5BAuthor%5D&cauthor=true&cauthor_uid=26198159)., [Keller, A.J](https://www.ncbi.nlm.nih.gov/pubmed/?term=Keller%20AJ%5BAuthor%5D&cauthor=true&cauthor_uid=26198159)., [Prado Scuracchio, P.S](https://www.ncbi.nlm.nih.gov/pubmed/?term=Prado%20Scuracchio%20PS%5BAuthor%5D&cauthor=true&cauthor_uid=26198159)., [Wendel, S](https://www.ncbi.nlm.nih.gov/pubmed/?term=Wendel%20S%5BAuthor%5D&cauthor=true&cauthor_uid=26198159)., [Andonov, A](https://www.ncbi.nlm.nih.gov/pubmed/?term=Andonov%20A%5BAuthor%5D&cauthor=true&cauthor_uid=26198159)., [Fearon, M](https://www.ncbi.nlm.nih.gov/pubmed/?term=Fearon%20M%5BAuthor%5D&cauthor=true&cauthor_uid=26198159)., [Delage, G](https://www.ncbi.nlm.nih.gov/pubmed/?term=Delage%20G%5BAuthor%5D&cauthor=true&cauthor_uid=26198159)., [Zhang, J](https://www.ncbi.nlm.nih.gov/pubmed/?term=Zhang%20J%5BAuthor%5D&cauthor=true&cauthor_uid=26198159)., [Shih, J.W](https://www.ncbi.nlm.nih.gov/pubmed/?term=Shih%20JW%5BAuthor%5D&cauthor=true&cauthor_uid=26198159)., [Gallian, P](https://www.ncbi.nlm.nih.gov/pubmed/?term=Gallian%20P%5BAuthor%5D&cauthor=true&cauthor_uid=26198159)., [Djoudi, R](https://www.ncbi.nlm.nih.gov/pubmed/?term=Djoudi%20R%5BAuthor%5D&cauthor=true&cauthor_uid=26198159)., [Tiberghien, P](https://www.ncbi.nlm.nih.gov/pubmed/?term=Tiberghien%20P%5BAuthor%5D&cauthor=true&cauthor_uid=26198159)., [Izopet, J](https://www.ncbi.nlm.nih.gov/pubmed/?term=Izopet%20J%5BAuthor%5D&cauthor=true&cauthor_uid=26198159)., [Dreier, J](https://www.ncbi.nlm.nih.gov/pubmed/?term=Dreier%20J%5BAuthor%5D&cauthor=true&cauthor_uid=26198159)., [Vollmer, T](https://www.ncbi.nlm.nih.gov/pubmed/?term=Vollmer%20T%5BAuthor%5D&cauthor=true&cauthor_uid=26198159)., [Knabbe, C](https://www.ncbi.nlm.nih.gov/pubmed/?term=Knabbe%20C%5BAuthor%5D&cauthor=true&cauthor_uid=26198159)., [Aggarwal, R](https://www.ncbi.nlm.nih.gov/pubmed/?term=Aggarwal%20R%5BAuthor%5D&cauthor=true&cauthor_uid=26198159)., [Goel , A](https://www.ncbi.nlm.nih.gov/pubmed/?term=Goel%20A%5BAuthor%5D&cauthor=true&cauthor_uid=26198159)., [Ciccaglione, A.R](https://www.ncbi.nlm.nih.gov/pubmed/?term=Ciccaglione%20AR%5BAuthor%5D&cauthor=true&cauthor_uid=26198159)., [Matsubayashi, K](https://www.ncbi.nlm.nih.gov/pubmed/?term=Matsubayashi%20K%5BAuthor%5D&cauthor=true&cauthor_uid=26198159)., [Satake, M](https://www.ncbi.nlm.nih.gov/pubmed/?term=Satake%20M%5BAuthor%5D&cauthor=true&cauthor_uid=26198159)., [Tadokoro, K](https://www.ncbi.nlm.nih.gov/pubmed/?term=Tadokoro%20K%5BAuthor%5D&cauthor=true&cauthor_uid=26198159)., [Jeong, S.H](https://www.ncbi.nlm.nih.gov/pubmed/?term=Jeong%20SH%5BAuthor%5D&cauthor=true&cauthor_uid=26198159)., [Zaaijer, H.L](https://www.ncbi.nlm.nih.gov/pubmed/?term=Zaaijer%20HL%5BAuthor%5D&cauthor=true&cauthor_uid=26198159)., [Zhiburt, E](https://www.ncbi.nlm.nih.gov/pubmed/?term=Zhiburt%20E%5BAuthor%5D&cauthor=true&cauthor_uid=26198159)., [Chay, J](https://www.ncbi.nlm.nih.gov/pubmed/?term=Chay%20J%5BAuthor%5D&cauthor=true&cauthor_uid=26198159)., [Teo, D](https://www.ncbi.nlm.nih.gov/pubmed/?term=Teo%20D%5BAuthor%5D&cauthor=true&cauthor_uid=26198159)., [Chua, S.S](https://www.ncbi.nlm.nih.gov/pubmed/?term=Chua%20SS%5BAuthor%5D&cauthor=true&cauthor_uid=26198159)., [Piron, M](https://www.ncbi.nlm.nih.gov/pubmed/?term=Piron%20M%5BAuthor%5D&cauthor=true&cauthor_uid=26198159)., [Sauleda, S](https://www.ncbi.nlm.nih.gov/pubmed/?term=Sauleda%20S%5BAuthor%5D&cauthor=true&cauthor_uid=26198159)., [Echevarría, J.M](https://www.ncbi.nlm.nih.gov/pubmed/?term=Echevarr%C3%ADa%20JM%5BAuthor%5D&cauthor=true&cauthor_uid=26198159)., [Dalton, H](https://www.ncbi.nlm.nih.gov/pubmed/?term=Dalton%20H%5BAuthor%5D&cauthor=true&cauthor_uid=26198159)., [Stramer, S.L](https://www.ncbi.nlm.nih.gov/pubmed/?term=Stramer%20SL%5BAuthor%5D&cauthor=true&cauthor_uid=26198159). 2016. Hepatitis E. Vox. Sang. 110, 93-130. doi: 10.1111/vox.12285. Epub 2015 Jul 21.

[Siripanyaphinyo, U](https://www.ncbi.nlm.nih.gov/pubmed/?term=Siripanyaphinyo%20U%5BAuthor%5D&cauthor=true&cauthor_uid=24984976)., [Boon-Long, J](https://www.ncbi.nlm.nih.gov/pubmed/?term=Boon-Long%20J%5BAuthor%5D&cauthor=true&cauthor_uid=24984976)., [Louisirirotchanakul, S](https://www.ncbi.nlm.nih.gov/pubmed/?term=Louisirirotchanakul%20S%5BAuthor%5D&cauthor=true&cauthor_uid=24984976)., [Takeda, N](https://www.ncbi.nlm.nih.gov/pubmed/?term=Takeda%20N%5BAuthor%5D&cauthor=true&cauthor_uid=24984976)., [Chanmanee, T](https://www.ncbi.nlm.nih.gov/pubmed/?term=Chanmanee%20T%5BAuthor%5D&cauthor=true&cauthor_uid=24984976)., [Srimee, B](https://www.ncbi.nlm.nih.gov/pubmed/?term=Srimee%20B%5BAuthor%5D&cauthor=true&cauthor_uid=24984976)., [Namsai, A](https://www.ncbi.nlm.nih.gov/pubmed/?term=Namsai%20A%5BAuthor%5D&cauthor=true&cauthor_uid=24984976)., [Pounsawa,t .P](https://www.ncbi.nlm.nih.gov/pubmed/?term=Pounsawat%20P%5BAuthor%5D&cauthor=true&cauthor_uid=24984976), [Khupulsap, K](https://www.ncbi.nlm.nih.gov/pubmed/?term=Khupulsap%20K%5BAuthor%5D&cauthor=true&cauthor_uid=24984976). 2014. Occurrence of hepatitis E virus infection in acute hepatitis in Thailand. J. Med. Virol. 86, 1730-1735. doi: 10.1002/jmv.24011. Epub 2014 Jul 2.

Widen, F., Sundqvist, L., Matyi-Toth, A., Metreveli, G., Belak, S., Hallgren, G. Norder, H. 2010. Molecular epidemiology of hepatitis E virus in humans, pigs and wild boars in Sweden. Epidemiol. Infect. 139, 361-371. doi: 10.1017/S0950268810001342.
