# Supplementary material for: Systematic review and meta-analysis of the seroprevalence of hepatitis E virus in the general population across non-endemic countries
Source: PLoS One. 2019 Jun 7;14(6):e0216826. doi: 10.1371/journal.pone.0216826 (PMC6555507; doi:10.1371/journal.pone.0216826)
Supplement: S4 File — (DOCX) [file pone.0216826.s006.docx]

# S4 File. Summary of preliminary analysis of selected country datasets, in ‘metafor’

| **Country** | **Number studies**  **(Number surveys)** | **Crude prevalence**  **(95% Cis)** | **Median age**  **(Num studies)** | **Prop male**  **(Num studies)** | **Year** | **Assay** | **Population** |
| --- | --- | --- | --- | --- | --- | --- | --- |
| **France** | 11 (11) | 0.252 | NS (4) | NS (4) | S | S | NS |
| **Germany** | 11(22) | 0.089 | NS (7) | NS (11) | NS | S | NS |
| **Italy** | 9 (11) | 0.046 | NS (9) | NS (8) | S | 0.05<p<0.1 | NS |
| **Japan** | 14 (18) | 0.041 | NS (12) | NS (13) | NS | NS | NS |
| **Netherlands** | 9 (16) | 0.121 | NS (2) | NS (4) | S | S | NS |
| **UK** | 6 (10) | 0.109 | NS (1) | NS (5) | 0.05<p<0.1 | S | NS |
|  |  |  |  |  |  |  |  |
| **USA** | 14 (21) | 0.077 | n/a (1) | NS (6) | NS | NS | NS |

Robumeta generates a warning code for each indicated computation " do not trust if df>4"- which is true for all of these country datasets. Only one study in the US dataset reported age in a usable form for our purposes in multilevel analysis in metafor, none of these datasets met criteria for properly specified model based on likelihood plots.

**Description of variables *a priori* identified for the modeling strategy of HEV IgG seroprevalence among general population and blood donor samples.**

| **Univariate analysis** | | | |
| --- | --- | --- | --- |
|  | Nature of variable | Metrics captured from individual studies | Significance (p<0.20)^1^ |
| Age | Continuous | Median | Ns |
| Sex | continuous | proportion male | Ns |
| Ethnicity | Categorical | Proportion of study population from ethnic groups as described by the author | Sig |
| Location | Categorical | Country, regions within country | Sig |
| Sample population | Categorical | Blood donor vs. general population | Ns |
| Assay | Categorical | Brands of commercial kits, or ‘in-house’ developed assays | Sig |
| Chronological time | Continuous | Year of sampling minus 1990 and centred^2^ | Sig |

^1^ Ns = non-significant S = Significant
