# Supplementary material for: Systematic review and meta-analysis of the seroprevalence of hepatitis E virus in the general population across non-endemic countries
Source: PLoS One. 2019 Jun 7;14(6):e0216826. doi: 10.1371/journal.pone.0216826 (PMC6555507; doi:10.1371/journal.pone.0216826)
Supplement: S5 File — (DOCX) [file pone.0216826.s007.docx]

# S5 File. Meta-analysis datasets

| **refid** | **Citation** | **Year** | **country** | **Population^1^** | **Assay^2^** | **Pos^3^** | **total** |
| --- | --- | --- | --- | --- | --- | --- | --- |
| 4524 | Atiq et al., 2009 | 2009 | USA | GP | MP Biomedical | 4 | 38 |
| 4886 | Dawson et al., 1992 | 1992 | USA | GP | In-house | 4 | 90 |
| 4931 | Ditah et al., 2014 | 2014 | USA | GP | Other | 490 | 8814 |
| 4935 | Dong et al., 2011 | 2011 | USA | BD | In-house | 36 | 372 |
| 4935 | Dong et al., 2011 | 2011 | USA | BD | Other | 33 | 372 |
| 4993 | Engle et al., 2002 | 2002 | USA | BD | In-house | 35 | 230 |
| 5450 | Karetnyi et al., 1999 | 1999 | USA | BD | In-house | 11 | 332 |
| 5450 | Karetnyi et al., 1999 | 1999 | USA | BD | In-house | 0 | 111 |
| 5565 | Kuniholm et al., 2009 | 2009 | USA | GP | In-house | 3926 | 18695 |
| 5776 | Mast et al., 1997 | 1997 | USA | BD | In-house | 59 | 5000 |
| 5776 | Mast et al., 1997 | 1997 | USA | BD | MP Biomedical | 70 | 5000 |
| 5823 | Meng et al., 2002 | 2002 | USA | BD | In-house | 73 | 400 |
| 5823 | Meng et al., 2002 | 2002 | USA | BD | In-house | 66 | 400 |
| 5962 | Obriadina et al., 2002 | 2002 | USA | BD | In-house | 11 | 92 |
| 5962 | Obriadina et al., 2002 | 2002 | USA | BD | In-house | 30 | 200 |
| 5962 | Obriadiet al., 2002 | 2002 | USA | BD | In-house | 13 | 75 |
| 5983 | Ooi et al., 1999 | 1999 | USA | GP | In-house | 9 | 384 |
| 6503 | Stramer et al., 2016 | 2016 | USA | BD | MP Biomedical | 329 | 4499 |
| 8572 | Tesh et al., 2015 | 2015 | USA | GP | Other | 653 | 4721 |
| 8572 | Tesh et al., 2015 | 2015 | USA | GP | Other | 303 | 6058 |
| 6862 | Wang et al., 2013 | 2013 | USA | BD | Wantai | 200 | 916 |
| 6862 | Wang et al., 2013 | 2013 | USA | BD | Wantai | 164 | 1023 |
| 4576 | Beale et al., 2011 | 2011 | UK | BD | Wantai | 31 | 262 |
| 4588 | Bendall et al., 2010 | 2010 | UK | BD | Wantai | 81 | 500 |
| 4588 | Bendall et al., 2010 | 2010 | UK | BD | MP Biomedical | 18 | 500 |
| 4805 | Cleland et al., 2013 | 2013 | UK | BD | Wantai | 73 | 1559 |
| 4805 | Cleland et al., 2013 | 2013 | UK | BD | Wantai | 30 | 528 |
| 4872 | Dalton et al., 2011 | 2011 | UK | GP | Wantai | 64 | 464 |
| 4873 | Dalton et al., 2008 | 2008 | UK | BD | Wantai | 77 | 487 |
| 4874 | Dalton et al., 2008 | 2008 | UK | BD | Wantai | 80 | 500 |
| 5325 | Ijaz et al., 2009 | 2009 | UK | GP | Wantai | 148 | 1140 |
| 5325 | Ijaz et al., 2009 | 2009 | UK | GP | Wantai | 215 | 1591 |
| 5460 | Kaufmann , 2011 | 2011 | Switzerland | BD | MP Biomedical | 27 | 550 |
| 6349 | Schnegg et al., 2013 | 2013 | Switzerland | BD | MP Biomedical | 27 | 550 |
| 6349 | Schnegg et al., 2013 | 2013 | Switzerland | BD | Dia.Pro | 23 | 550 |
| 6349 | Schnegg et al., 2013 | 2013 | Switzerland | BD | Wantai | 120 | 550 |
| 8524 | Lavanchy et al., 1994 | 1994 | Switzerland | BD | Abbott | 3 | 94 |
| 8982 | Niederhauser, 2016 | 2016 | Switzerland | BD | Wantai | 704 | 3609 |
| 5947 | Norder et al., 2016 | 2016 | Sweden | BD | Mikrogen | 33 | 500 |
| 5947 | Norder et al., 2016 | 2016 | Sweden | BD | DSI | 51 | 500 |
| 5947 | Norder et al., 2016 | 2016 | Sweden | BD | Other | 24 | 500 |
| 5947 | Norder et al., 2016 | 2016 | Sweden | BD | Axiom | 80 | 500 |
| 5947 | Norder et al., 2016 | 2016 | Sweden | BD | Dia.Pro | 95 | 500 |
| 5979 | Olsen et al., 2006 | 2006 | Sweden | GP | Abbott | 10 | 108 |
| 6530 | Sylvan et al., 1998 | 1998 | Sweden | GP | Abbott | 18 | 349 |
| 4686 | Buti et al., 2006 | 2006 | Spain | GP | Bioelisa | 96 | 1280 |
| 4688 | Buti et al., 1995 | 1995 | Spain | GP | Abbott | 3 | 72 |
| 5045 | Fogeda et al., 2012 | 2012 | Spain | GP | Other | 50 | 2305 |
| 5715 | Lopez-Fabal , 2015 | 2015 | Spain | GP | Genelabs | 24 | 200 |
| 5779 | Mateos et al., 1999 | 1999 | Spain | BD | Abbott | 25 | 863 |
| 5813 | Medrano et al., 1995 | 1995 | Spain | BD | Abbott | 20 | 483 |
| 6224 | Riveiro-Barciela | 2014 | Spain | GP | Bioelisa | 7 | 200 |
| 6331 | Sauleda et al., 2015 | 2015 | Spain | BD | Wantai | 216 | 1082 |
| 6331 | Sauleda et al., 2015 | 2015 | Spain | BD | Mikrogen | 116 | 1082 |
| 4403 | Abdelaal et al., 1998 | 1998 | Saudi | BD | In-house | 100 | 593 |
| 4510 | Arif et al., 1994 | 1994 | Saudi | GP | Abbott | 66 | 788 |
| 4510 | Arif et al., 1994 | 1994 | Saudi | GP | Abbott | 94 | 630 |
| 4531 | Ayoola et al., 2002 | 2002 | Saudi | GP | Abbott L | 43 | 400 |
| 5393 | Johargy et al., 2013 | 2013 | Saudi | BD | Bioelisa | 168 | 900 |
| 6035 | Paul et al., 1994 | 1994 | Saudi | BD | In-house | 82 | 861 |
| 6035 | Paul et al., 19942 | 1994 | Saudi | BD | In-house | 113 | 7963 |
| 8621 | Elsheikh et al., 2012 | 2012 | Saudi | GP | Other | 1 | 720 |
| 5962 | Obriadina , 2002 | 2002 | Russia | BD | In-house | 33 | 185 |
| 8992 | Nasrallah et al., 2016 | 2016 | Qatar | BD | Wantai | 1019 | 5042 |
| 5831 | Mesquita, 2014 | 2014 | Portugal | GP | Wantai | 16 | 120 |
| 8987 | Sargento et al., 2016 | 2016 | Portugal | BD | Other | 5 | 238 |
| 9005 | Teixeira et al., 2017 | 2017 | Portugal | GP | Mikrogen | 160 | 804 |
| 9020 | Pereira et al., 2016 | 2016 | Portugal | GP | Mikrogen | 149 | 1656 |
| 9223 | Sargento et al., 2014 | 2014 | Portugal | BD | Mikrogen | 6 | 231 |
| 8986 | Sulkowska , 2016 | 2016 | Poland | BD | Wantai | 1053 | 2408 |
| 8984 | Bukow et al., 2016 | 2016 | Poland | GP | Other | 9 | 261 |
| 4877 | Dalton et al., 2007 | 2007 | NZ | BD | Wantai | 11 | 265 |
| 4468 | Andenaes , 2000 | 2000 | Norway | GP | Abbott | 1 | 199 |
| 4468 | Andenaes , 2000 | 2000 | Norway | GP | Abbott | 0 | 204 |
| 5588 | Lange et al., 2016 | 2016 | Norway | BD | Wantai | 162 | 1200 |
| 5469 | Bouwknegt et al., 2008 | 2008 | Netherlands |  | Abbott Laboratories | 11 | 648 |
| 5469 | Bouwknegt et al., 2008 | 2008 | Netherlands |  | MP Biomedical | 27 | 648 |
| 5236 | Herremans, 2007 | 2007 | Netherlands | GP | MP Biomedical | 12 | 167 |
| 5238 | Herremans , 2007 | 2007 | Netherlands | BD | MP Biomedical | 3 | 50 |
| 5261 | Hogema et al., 2014 | 2014 | Netherlands | BD | Wantai | 23 | 116 |
| 5261 | Hogema et al., 2014 | 2014 | Netherlands | BD | Wantai | 5 | 116 |
| 5261 | Hogema et al., 2014 | 2014 | Netherlands | BD | Wantai | 7 | 100 |
| 5261 | Hogema et al., 2014 | 2014 | Netherlands | BD | Wantai | 23 | 181 |
| 6263 | Sadik et al., 2016 | 2016 | Netherlands | GP | Wantai | 459 | 1199 |
| 6449 | Slot et al., 2013 | 2013 | Netherlands | BD | Wantai | 1401 | 5239 |
| 6703 | van den Berg, 2014 | 2014 | Netherlands | GP | Wantai | 77 | 201 |
| 6727 | Verhoef et al., 2012 | 2012 | Netherlands | GP | MP Biomedical | 107 | 5642 |
| 6917 | Zaaijer et al., 1995 | 1995 | Netherlands | BD | Abbott | 5 | 1275 |
| 6918 | Zaaijer et al., 1992 | 1992 | Netherlands | BD | MP Biomedical | 5 | 275 |
| 4442 | Ahn et al., 2005 | 2005 | South Korea | GP | MP Biomedical | 43 | 361 |
| 4786 | Choi et al., 2003 | 2003 | South Korea | BD | MP Biomedical | 17 | 96 |
| 6025 | Park et al., 2012 | 2012 | South Korea | GP | Wantai | 34 | 147 |
| 6025 | Park et al., 2012 | 2012 | South Korea | GP | MP Biomedical | 21 | 147 |
| 6908 | Yoon et al., 2014 | 2014 | South Korea | GP | Wantai | 144 | 2450 |
| 5064 | Fukuda et al., 2007 | 2007 | Japan | BD | In-house | 168 | 3185 |
| 5064 | Fukuda et al., 2007 | 2007 | Japan | BD | In-house | 31 | 594 |
| 5064 | Fukuda et al., 2007 | 2007 | Japan | BD | In-house | 9 | 156 |
| 5064 | Fukuda et al., 2007 | 2007 | Japan | BD | In-house | 5 | 116 |
| 5065 | Fukuda et al., 2004 | 2004 | Japan | BD | In-house | 200 | 5343 |
| 5133 | Gotanda et al., 2007 | 2007 | Japan | BD | In-house | 479 | 6700 |
| 5656 | Li et al., 2000 | 2000 | Japan | GP | In-house | 49 | 900 |
| 5852 | Mitsui et al., 2005 | 2005 | Japan | GP | In-house | 6 | 266 |
| 6281 | Sakata et al., 2008 | 2008 | Japan | BD | In-house | 6 | 1062 |
| 6546 | Takahashi , 2010 | 2010 | Japan | GP | In-house | 1167 | 22027 |
| 6552 | Takahashi, 2005 | 2005 | Japan | BD | In-house | 16 | 675 |
| 6557 | Takeda et al., 2010 | 2010 | Japan | BD | In-house | 431 | 12600 |
| 6601 | Tei et al., 2004 | 2004 | Japan | GP | Other | 1 | 45 |
| 6643 | Toyoda et al., 2008 | 2008 | Japan | GP | In-house | 26 | 526 |
| 6643 | Toyoda et al., 2008 | 2008 | Japan | GP | In-house | 42 | 266 |
| 7081 | Ding et al., 2003 | 2003 | Japan | GP | In-house | 6 | 200 |
| 8971 | Fukae et al., 2016 | 2016 | Japan | GP | Other | 1 | 60 |
| 4710 | Caruso et al., 2016 | 2016 | Italy | GP | Wantai | 1 | 73 |
| 5112 | Gessoni et al., 1996 | 1996 | Italy | GP | Abbott | 49 | 1889 |
| 5708 | Lucarelli et al., 2016 | 2016 | Italy | BD | Wantai | 153 | 313 |
| 5772 | Masia et al., 2009 | 2009 | Italy | BD | Diagnostic Bioprob | 20 | 402 |
| 6039 | Pavia et al., 1998 | 1998 | Italy | BD | Abbott | 6 | 361 |
| 6039 | Pavia et al., 1998 | 1998 | Italy | BD | Other | 4 | 361 |
| 6133 | Puttini et al., 2015 | 2015 | Italy | BD | EIAgen (Adaltis) | 12 | 132 |
| 6166 | Rapicetta et al., 2013 | 2013 | Italy | BD | DSI | 5 | 100 |
| 6209 | Ricco et al., 2016 | 2016 | Italy | BD | Wantai | 9 | 199 |
| 6365 | Scotto et al., 2014 | 2014 | Italy | GP | Dia.Pro | 12 | 450 |
| 6365 | Scotto et al., 2014 | 2014 | Italy | BD | Dia.Pro | 2 | 151 |
| 6922 | Zanetti et al., 1994 | 1994 | Italy | BD | Abbott | 9 | 948 |
| 6922 | Zanetti et al., 1994 | 1994 | Italy | GP | Abbott | 12 | 1629 |
| 5875 | Mor et al., 2015 | 2015 | Israel | BD | DSI | 77 | 729 |
| 8775 | Keretnyi et al., 1996 | 1996 | Israel | BD | In-house | 7 | 48 |
| 5958 | O'Riordan , 2016 | 2016 | Ireland | BD | Wantai | 57 | 1076 |
| 7364 | Hickey et al., 2016 | 2016 | Ireland | GP | Wantai | 16 | 198 |
| 9023 | Love et al., 2018 | 2018 | Iceland | GP | Dia.Pro | 18 | 195 |
| 9023 | Love et al., 2018 | 2018 | Iceland | GP | Wantai | 12 | 195 |
| 5695 | Lok et al., 1992_1 | 1992 | Hong Kong | GP | MPBiomedical | 57 | 355 |
| 6823 | Wong et al., 2004 | 2004 | Hong Kong | GP | MPBiomedical | 176 | 934 |
| 4867 | Dalekos et al., 1998 | 1998 | Greece | BD | Abbott | 6 | 2636 |
| 4867 | Dalekos et al., 1998 | 1998 | Greece | BD | Abbott | 2 | 380 |
| 6090 | Pittaras et al., 2014 | 2014 | Greece | BD | EIAgen (Adaltis) | 25 | 265 |
| 9143 | Zervou et al., 2016 | 2015 | Greece | BD | MP Biomedical | 36 | 1200 |
| 6120 | Psichogiou et al., 1996 | 1996 | Greece | GP | Abbott | 7 | 316 |
| 4574 | Baylis et al., 2010 | 2010 | Germany | BD | MP Biomedical | 6 | 109 |
| 4886 | Dawson et al., 1992 | 1992 | Germany | BD | In-house | 11 | 151 |
| 4886 | Dawson et al., 1992 | 1992 | Germany | BD | In-house | 9 | 386 |
| 4948 | Dremsek et al., 2012 | 2012 | Germany | BD | Mikrogen | 33 | 298 |
| 4948 | Dremsek et al., 2012 | 2012 | Germany | BD | In-house | 37 | 298 |
| 5005 | Faber et al., 2012 | 2012 | Germany | GP | Mikrogen | 731 | 4352 |
| 5407 | Juhl et al., 2014 | 2014 | Germany | BD | Mikrogen | 84 | 1019 |
| 5544 | Krumbholz , 2014 | 2014 | Germany | GP | Mikrogen | 74 | 537 |
| 5544 | Krumbholz, 2014 | 2014 | Germany | GP | MP Biomedical | 22 | 537 |
| 5544 | Krumbholz, 2014 | 2014 | Germany | GP | Axiom | 150 | 537 |
| 5544 | Krumbholz, 2014 | 2014 | Germany | GP | In-house | 58 | 537 |
| 5546 | Krumbholz,2014 | 2012 | Germany | BD | Mikrogen | 18 | 116 |
| 6083 | Pischke et al., 2014 | 2014 | Germany | BD | MP Biomedical | 11 | 537 |
| 6086 | Pischke et al., 2011 | 2011 | Germany | BD | Abbott | 11 | 537 |
| 6189 | Reinheimer, 2012 | 2012 | Germany | BD | Mikrogen | 4 | 50 |
| 6750 | Vollmer et al., 2012 | 2012 | Germany | BD | Mikrogen | 8 | 136 |
| 6750 | Vollmer et al., 2012 | 2012 | Germany | BD | Mikrogen | 12 | 200 |
| 6798 | Wentz et al., 2013 | 2013 | Germany | GP | MP Biomedical | 9 | 200 |
| 6798 | Wentz et al., 2013 | 2013 | Germany | GP | Wantai | 59 | 200 |
| 6798 | Wentz et al., 2013 | 2013 | Germany | GP | Mikrogen | 36 | 200 |
| 8355 | Pischke et al., 2010 | 2010 | Germany | GP | Abbott | 3 | 168 |
| 4651 | Boutrouille , 2007 | 2007 | France | BD | MP Biomedical | 64 | 1998 |
| 4708 | Carpentier , 2012 | 2012 | France | GP | MP Biomedical | 26 | 135 |
| 4747 | Chaussade , 2013 | 2013 | France | GP | MP Biomedical | 84 | 322 |
| 4835 | Coursaget , 1994 | 1994 | France | GP | In-house | 1 | 278 |
| 5074 | Gallian et al., 2014 | 2014 | France | BD | Wantai | 203 | 861 |
| 5352 | Izopet et al., 2015 | 2015 | France | BD | Wantai | 351 | 1031 |
| 5752 | Mansuy et al., 2016 | 2016 | France | BD | Wantai | 2371 | 10569 |
| 5753 | Mansuy et al., 2015 | 2015 | France | BD | Wantai | 1311 | 3353 |
| 5754 | Mansuy et al., 2011 | 2011 | France | BD | Wantai | 268 | 512 |
| 5756 | Mansuy et al., 2008 | 2008 | France | BD | MP Biomedical | 88 | 529 |
| 8366 | Dalton et al., 2010 | 2010 | France | BD | Wantai | 269 | 512 |
| 4792 | Christensen, 2008 | 2008 | Denmark | BD | In-house | 55 | 167 |
| 4792 | Christensen, 2008 | 2008 | Denmark | BD | In-house | 94 | 456 |
| 5262 | Holm et al., 2015 | 2015 | Denmark | BD | In-house | 54 | 504 |
| 5262 | Holm et al., 2015 | 2015 | Denmark | BD | Wantai | 100 | 504 |
| 6502 | Strakova et al., 2014 | 2014 | Czech | GP | Dia.Pro | 13 | 230 |
| 9087 | Nemecek et al., 2015 | 2015 | Czech | GP | Other | 43 | 1719 |
| 6736 | Vilibic-Cavlek , 2016 | 2016 | Croatia | GP | Other | 1 | 37 |
| 8985 | Miletic Lovric , 2016 | 2016 | Croatia | BD | Dia.Pro | 210 | 1036 |
| 4905 | Debes et al., 2016 | 2016 | Argentina | GP | Dia.Pro | 19 | 433 |
| 5888 | Munne et al., 2014 | 2014 | Argentina | BD | Wantai | 4 | 24 |
| 5888 | Munne et al., 2014 | 2014 | Argentina | GP | Dia.Pro | 9 | 95 |
| 5888 | Munne et al., 2014 | 2014 | Argentina | GP | Wantai | 4 | 28 |
| 5888 | Munne et al., 2014 | 2014 | Argentina | GP | Wantai | 4 | 27 |
| 7225 | Rey et al., 1997 | 1997 | Argentina | BD | Abbott | 39 | 2157 |
| 5866 | Moaven et al., 1995 | 1995 | Australia | BD | MP Biomedical | 1 | 279 |
| 6409 | Shrestha et al., 2014 | 2014 | Australia | BD | Wantai | 194 | 3237 |
| 5037 | Fischer et al., 2015 | 2015 | Austria | BD | Wantai | 163 | 1203 |
| 9390 | Lagler et al., 2013 | 2013 | Austria | GP | Wantai | 143 | 997 |
| 9202 | Fearon et al., 2017 | 2017 | Canada | BD | Wantai | 241 | 4102 |

**^1^** BD = blood donors; GP = general population; IR = group potentially at increased risk of exposure to HEV; TPG = targeted patient group ^2^  DSI = DSI s.r.l. ^3^ Pos = subjects categorised as Positive
