# Supplementary material for: Systematic review and meta-analysis of the seroprevalence of hepatitis E virus in the general population across non-endemic countries
Source: PLoS One. 2019 Jun 7;14(6):e0216826. doi: 10.1371/journal.pone.0216826 (PMC6555507; doi:10.1371/journal.pone.0216826)
Supplement: S6 File — (DOCX) [file pone.0216826.s008.docx]

# S6 File. List of included studies

Abdelaal M, Zawawi TH, al Sobhi E, Jeje O, Gilpin C, Kinsara A, et al. Epidemiology of hepatitis E virus in male blood donors in Jeddah, Saudi Arabia. 1998. Ir J Med Sci. 1998;167(2): 94-96.

Ahn JM, Kang SG, Lee DY, Shin SJ, Yoo HS. Identification of novel human hepatitis E virus (HEV) isolates and determination of the seroprevalence of HEV in Korea. J Clin Microbiol. 2005;43(7): 3042–3048. doi:10.1128/JCM.43.7.3042–3048.2005.

## Andenaes S, Lie A, Degre M. Prevalence of hepatitis A, B, C, and E antibody in flying airline personnel. Aviat Space Environ Med. 2000;71(12): 1178-1180.

## Arif M, Qattan I, al-Faleh F, Ramia S. Epidemiology of hepatitis E virus (HEV) infection in Saudi Arabia. Ann Trop Med Parasitol. 1994;88(2): 163-168.

## Atiq M, Shire NJ, Barrett A, Rouster SD, Sherman KE, Shata MT. Hepatitis E virus antibodies in patients with chronic liver disease. Emerg Infect Dis. 2009;15(3): 479-481. doi: 10.3201/eid1503.080740.

## Ayoola EA, Want MA, Gadour MOEH, Al-Hazmi MH, Hamza MKM. Hepatitis E virus infection in haemodialysis patients: a case-control study in Saudi Arabia. J Med Virol. 2002;66(3): 329-334.

Baylis SA, Nick S, Blumel J, Nubling CM. Hepatitis E virus and blood donors in Germany. Vox Sang. 2010;98: 479. DOI: 10.1111/j.1423-0410.2009.01258.x.

Beale MA, Tettmar K, Szypulska R, Tedder RS, Ijaz S. Is there evidence of recent hepatitis E virus infection in English and North Welsh blood donors? Vox Sang. 2011;100(3): 340–342. DOI: 10.1111/j.1423-0410.2010.01412.x.

Begg CB, Mazumdar M. Operating characteristics of a rank correlation test for publication bias. Biometrics. 1994;50(4): 1088–1101. DOI: 10.2307/2533446.

## Bendall R, Ellis V, Ijaz S, Ali R, Dalton H. A comparison of two commercially available anti-HEV IgG kits and a re-evaluation of anti-HEV IgG seroprevalence data in developed countries. J Med Virol. 2010;82(5): 799-805. DOI 10.1002/jmv.21656.

Boutrouille A, Bakkali-Kassimi L, Cruciere C, Pavio N. Prevalence of anti-hepatitis E virus antibodies in French blood donors. J Clin Microbiol. 2007;45(6): 2009–2010. DOI: 10.1128/JCM.00235-07.

[Bouwknegt M](https://www.ncbi.nlm.nih.gov/pubmed/?term=Bouwknegt%20M%5BAuthor%5D&cauthor=true&cauthor_uid=17578603), [Engel B](https://www.ncbi.nlm.nih.gov/pubmed/?term=Engel%20B%5BAuthor%5D&cauthor=true&cauthor_uid=17578603), [Herremans MM](https://www.ncbi.nlm.nih.gov/pubmed/?term=Herremans%20MM%5BAuthor%5D&cauthor=true&cauthor_uid=17578603), [Widdowson MA](https://www.ncbi.nlm.nih.gov/pubmed/?term=Widdowson%20MA%5BAuthor%5D&cauthor=true&cauthor_uid=17578603), [Worm HC](https://www.ncbi.nlm.nih.gov/pubmed/?term=Worm%20HC%5BAuthor%5D&cauthor=true&cauthor_uid=17578603), [Koopmans MP](https://www.ncbi.nlm.nih.gov/pubmed/?term=Koopmans%20MP%5BAuthor%5D&cauthor=true&cauthor_uid=17578603), et al. Bayesian estimation of hepatitis E virus seroprevalence for populations with different exposure levels to swine in The Netherlands. Epidemiol Infect. 2008;136: 567-576. doi:10.1017/S0950268807008941.

Bukowska A, Piersiala K, Olbromski H, Skalisz H. Evaluation of the prevalence of anti-HEV IgM and IgG antibodies in 3 groups: A group of healthy people, a group of professional foresters and a group of HIV-infected people. Vox Sang. 2016;111(S1): 200-201.

Buti M, Dominguez A, Plans P, Jardi R, Schaper M, Espunes J, et al. Community-based seroepidemiological survey of hepatitis E virus infection in Catalonia, Spain. Clin Vaccine Immunol. 2006;13(12): 1328–1332. doi:10.1128/CVI.00255-06.

## Buti M, Jardi R, Cotrina M, Rodriguez-Frias F, Troonen H, Viladomiu L, et al. Hepatitis E virus infection in acute hepatitis in Spain. J Virol Methods. 1995;55(1): 49-54.

Carpentier A, Chaussade H, Rigaud E, Rodriguez J, Berthault C, Boue F, et al. 2012. High hepatitis E virus seroprevalence in forestry workers and in wild boars in France. J Clin Microbiol. 2012;50(9): 2888–2893. doi: 10.1128/JCM.00989-12.

Caruso C, Peletto S, Rosamilia A, Modesto P, Chiavacci L, Sona B, et al. Hepatitis E virus: a cross-sectional serological and virological study in pigs and humans at zoonotic risk within a high-density pig farming area. Transbound Emerg Dis. 2017;64(5): 1443-1453. doi:10.1111/tbed.12533.

Caspari G, Barbara JA. Anti-HEV in blood donors: what is test and what is virus? Vox Sang. 2000;78(3): 198-199. DOI: 10.1159/000031180.

Chaussade H, Rigaud E, Allix A, Carpentier A, Touze A, Delzescaux D, et al. Hepatitis E virus seroprevalence and risk factors for individuals in working contact with animals. J Clin Virol. 2013;58(3): 504-508. doi: 10.1016/j.jcv.2013.08.030.

## Choi IS, Kwon HJ, Shin NR, Yoo HS. Identification of swine hepatitis E virus (HEV) and prevalence of anti-HEV antibodies in swine and human populations in Korea. J Clin Microbiol. 2003;41(8): 3602-3608. DOI: 10.1128/JCM.41.8.3602–3608.2003.

Christensen PB, Engle RE, Hjort C, Homburg KM, Vach W, Georgsen J, et al. Time trend of the prevalence of hepatitis E antibodies among farmers and blood donors: a potential zoonosis virus in Denmark. Clin Infect Dis. 2008;47(8): 1026-1031. doi: 10.1086/591970.

Cleland A, Smith L, Crossan C, Blatchford O, Dalton HR, Scobie L, et al. Hepatitis E virus in Scottish blood donors. Vox Sang. 2013;105: 283–289. DOI: 10.1111/vox.12056.

Coursaget P, Depril N, Buisson Y, Molinie C, Roue R. Hepatitis type E in a French population: detection of anti-HEV by a synthetic peptide-based enzyme-linked immunosorbent assay. Res Virol. 1994;145(1): 51-57.

Dalekos GN, Zervou E, Elisaf M, Germanos N, Galanakis E, Bourantas K, et al. Antibodies to hepatitis E virus among several populations in Greece: increased prevalence in an hemodialysis unit. Transfusion. 1998;38: 589-595.

Dalton HR, Bendall RP, Rashid M, Ellis V, Ali R, Ramnarace R, et al. Host risk factors and autochthonous hepatitis E infection. Eur J Gastroenterol Hepatol. 2011;23(12): 1200-1205. DOI: 10.1097/MEG.0b013e32834ca4da.

Dalton HR, Stableforth W, Hazeldine S, Thurairajah P, Ramnarace R, Warshow U, et al. Autochthonous hepatitis E in Southwest England: a comparison with hepatitis A. Eur J Clin Microbiol Infect Dis. 2008;27(7): 579–585. DOI 10.1007/s10096-008-0480-z.

Dalton HR, Fellows HJ, Gane EJ, Wong P, Gerred S, Schroeder B, et al. Hepatitis E in New Zealand. J Gastroenterol Hepatol. 2007;22(8): 1236–1240. doi:10.1111/j.1440-1746.2007.04894.x.

## Dalton H, Mansuy JM, Bendall R, Legrand-Abravanel F, Calot JP, Kamar N, et al. Hepatitis E virus is highly endemic in South West France. Gut. 2010;59: A43. doi:10.1136/gut.2010.223362.105.

Dawson GJ, Chau KH, Cabal CM, Yarbough PO, Reyes GR, Mushahwar IK. Solid-phase enzyme-linked immunosorbent assay for hepatitis E virus IgG and IgM antibodies utilizing recombinant antigens and synthetic peptides. J Virol Methods. 1992;38: 175-186.

## Debes JD, Martinez Wassaf M, Pisano MB, Isa MB, Lotto M, Marianelli LG, et al. Increased hepatitis E virus seroprevalence correlates with lower CD4+ cell counts in HIV-infected persons in Argentina. PLoS ONE 2016;11(7): e0160082. doi:10.1371/journal.pone.0160082.

## Ding X, Li TC, Hayashi S, Masaki N, Tran TTH, Hirano M, et al. Present state of hepatitis E virus epidemiology in Tokyo, Japan. Hepatol Res. 2003;27(3): 169-173. doi:10.1016/S1386-6346(03)00233-X.

## Ditah I, Ditah F, Devaki P, Ditah C, Kamath PS, Charlton M. Current epidemiology of hepatitis E virus infection in the United States: low seroprevalence in the National Health and Nutrition Evaluation Survey. Hepatology. 2014;60(3): 815-822. doi: 10.1002/hep.27219.

Dong C, Meng J, Dai X, Liang JH, Feagins AR, Meng XJ, et al. Restricted enzooticity of hepatitis E virus genotypes 1 to 4 in the United States. J Clin Microbiol. 2011;49(12): 4164–4172. doi:10.1128/JCM.05481-11.

Dremsek P, Wenzel JJ, Johne R, Ziller M, Hofmann J, Groschup MH, et al. Seroprevalence study in forestry workers from eastern Germany using novel genotype 3- and rat hepatitis E virus-specific immunoglobulin G ELISAs. Med Microbiol Immunol. 2012;201: 189–200. DOI 10.1007/s00430-011-0221-2.

Elsheikh AA, Alqurashi AM. Seroprevelence of hepatitis E virus in human and animals in southwestern Saudi Arabia. J Am Sci. 2012;8(11): 674-677.

Engle RE, Yu C, Emerson SU, Meng XJ, Purcell RH. Hepatitis E virus (HEV) capsid antigens derived from viruses of human and swine origin are equally efficient for detecting anti-HEV by enzyme immunoassay. J Clin Microbiol. 2002;40(12): 4576–4580. DOI: 10.1128/JCM.40.12.4576–4580.2002.

## Faber MS, Wenzel JJ, Jilg W, Thamm M, Hohle M, Stark K. Hepatitis E virus seroprevalence among adults, Germany. Emerg Infect Dis. 2012;18(10): 1654-1657. DOI: http://dx.doi.org/10.3201/eid1810.111756.

## Fearon MA, O'Brien SF, Delage G, Scalia V, Bernier F, Bigham M, et al. Hepatitis E in Canadian blood donors. Transfus*.* 2017;57(6): 1420-1425. https://doi.org/10.1111/trf.14089.

## Fischer C, Hofmann M, Danzer M, Hofer K, Kaar J, Gabriel C. Seroprevalence and incidence of hepatitis E in blood donors in Upper Austria. PLoS ONE 2015;10(3): e0119576. doi:10.1371/journal.pone.0119576.

## Fogeda M, Avellon A, Echevarria JM. Prevalence of specific antibody to hepatitis E virus in the general population of the community of Madrid, Spain. J Med Virol. 2012;84(1): 71–74. doi: 10.1002/jmv.22270.

Freeman MF, Tukey JW. Transformations related to the angular and the square root. Ann Math Stat. 1950;21(4): 607-611.

## Fukae J, Tsugawa J, Ouma S, Umezu T, Kusunoki S, Tsuboi Y. Guillain–Barré and Miller Fisher syndromes in patients with anti-hepatitis E virus antibody: a hospital-based survey in Japan. Neurol Sci. 2016;37(11): 1849-1851. DOI 10.1007/s10072-016-2644-4.

## Fukuda S, Ishikawa M, Ochiai N, Suzuki Y, Sunaga J, Shinohara N, et al. Unchanged high prevalence of antibodies to hepatitis E virus (HEV) and HEV RNA among blood donors with an elevated alanine aminotransferase level in Japan during 1991-2006. Arch Virol. 2007;152: 1623–1635. DOI 10.1007/s00705-007-0996-z

## Fukuda S, Sunaga J, Saito N, Fujimura K, Itoh Y, Sasaki M, et al. Prevalence of antibodies to hepatitis E virus among Japanese blood donors: identification of three blood donors infected with a genotype 3 hepatitis E virus. J Med Virol. 2004;73: 554–561. DOI 10.1002/jmv.20125.

## Gallian P, Lhomme S, Piquet Y, Saune K, Abravanel F, Assal A, et al. Hepatitis E virus infections in blood donors, France. Emerg Infect Dis. 2014;20(11): 1914-1917. DOI: http://dx.doi.org/10.3201/eid2011.140516

## Gessoni G, Manoni F. Hepatitis E virus infection in north-east Italy: serological study in the open population and groups at risk. J Viral Hepat. 1996;3: 197-202.

## Gotanda Y, Iwata A, Ohnuma H, Yoshikawa A, Mizoguchi H, Endo K, et al. Ongoing subclinical infection of hepatitis E virus among blood donors with an elevated alanine aminotransferase level in Japan. J Med Virol. 2007;79: 734–742. DOI 10.1002/jmv.20834.

Herremans M, Vennema H, Bakker J, van der Veer B, Duizer E, Benne CA, et al. Swine-like hepatitis E viruses are a cause of unexplained hepatitis in the Netherlands. J Viral Hepat. 2007a;14(2): 140–146. doi:10.1111/j.1365-2893.2006.00786.x.

Herremans M, Bakker J, Duizer E, Vennema H, Koopmans MPG. Use of serological assays for diagnosis of hepatitis E virus genotype 1 and 3 infections in a setting of low endemicity. Clin Vaccine Immunol. 2007b;14(5): 562–568. doi:10.1128/CVI.00231-06.

Hickey C, Spillane D, Benson J, Levis J, Fanning LJ, Cryan B, et al. Hepatitis E virus (HEV) infection in Ireland. Ir Med J. 2016;109(8): 451.

Hogema BM, Molier M, Slot E, Zaaijer HL. Past and present of hepatitis E in the Netherlands. Transfusion. 2014;54(12): 3092-3096. doi: 10.1111/trf.12733.

Holm DK, Moessner BK, Engle RE, Zaaijer HL, Georgsen J, Purcell RH, et al. Declining prevalence of hepatitis E antibodies among Danish blood donors. Transfusion. 2015;55(7): 1662–1667. doi: 10.1111/trf.13028.

## Ijaz S, Vyse AJ, Morgan D, Pebody RG, Tedder RS, Brown D. Indigenous hepatitis E virus infection in England: more common than it seems. J Clin Virol. 2009;44(4): 272–276. doi:10.1016/j.jcv.2009.01.005.

Izopet J, Labrique AB, Basnyat B, Dalton HR, Kmush B, Heaney CD, et al. Hepatitis E virus seroprevalence in three hyperendemic areas: Nepal, Bangladesh and southwest France. J Clin Virol. 2015;70: 39-42. doi: 10.1016/j.jcv.2015.06.103.

## Johargy AK, Mahomed MF, Khan MM, Kabrah S. Anti hepatitis E virus seropositivity in a group of male blood donors in Makkah, Saudi Arabia. Anti hepatitis E virus seropositivity in a group of male blood donors in Makkah, Saudi Arabia. J Pak Med Assoc. 2013;63(2): 185-189.

Juhl D, Baylis SA, Blumel J, Gorg S, Hennig H. Seroprevalence and incidence of hepatitis E virus infection in German blood donors. Transfusion. 2014;54: 49-56**.** doi: 10.1111/trf.12121.

## Karetnyi YV, Favorov MO, Khudyakova NS, Bar-Shani S, Dagan R, Fields HA, et al. Populations with high prevalence of antibody against hepatitis E virus in Israel. Clin Diagn Virol. 1996;6(1): 73-76.

## Karetnyi YV, Gilchrist MJR, Naides SJ. Hepatitis E virus infection prevalence among selected populations in Iowa. J Clin Virol. 1999;14(1): 51-55.

## Kuniholm MH, Purcell RH, McQuillan GM, Engle RE, Wasley A, Nelson KE. Epidemiology of hepatitis E virus in the United States: results from the Third National Health and Nutrition Examination Survey, 1988-1994. J Infect Dis. 2009;200: 48–56. DOI: 10.1086/599319.

## Krumbholz A, Joel S, Dremsek P, Neubert A, Johne R, Durrwald R, et al. Seroprevalence of hepatitis E virus (HEV) in humans living in high pig density areas of Germany. Med Microbiol Immunol*.* 2014;203: 273–282. doi:10.1007/s00430-014-0336-3.

Khudyakov Y, Kamili S. Serological diagnostics of hepatitis E virus infection. Virus Res. 2011;161(1): 84-92. doi: 10.1016/j.virusres.2011.06.006.

Lagler H, Poeppl W, Winkler H, Herkner H, Faas A, Mooseder G, et al. Hepatitis E virus seroprevalence in Austrian adults: a nationwide cross-sectional study among civilians and military professionals. PloS One 2013;9(2): e87669. doi:10.1371/journal.pone.0087669.

Lange H, Overbo J, Borgen K, Dudman S, Hoddevik G, Urdahl AM, et al. Hepatitis E in Norway: seroprevalence in humans and swine. Epidemiol Infect. 2017;145: 181-186. doi:10.1017/S0950268816002144.

## Lavanchy, D, Morel B, Frei PC. Seroprevalence of hepatitis E virus in Switzerland. Lancet. 1994;344(8924): 747-748.

## Li TC, Zhang J, Shinzawa H, Ishibashi M, Sata M, Mast EE, et al. Empty virus-like particle-based enzyme-linked immunosorbent assay for antibodies to hepatitis E virus. J Med Virol. 2000;62(3): 327–333.

Lok ASF, Kwan WK, Moeckli R, Yarbough PO, Chan RT, Reyes GR, et al. Seroepidemiological survey of hepatitis E in Hong Kong by recombinant-based enzyme immunoassays. Lancet. 1992;340: 1205-1208.

Love A, Björnsdottir TB, Olafsson S, Bjornsson ES. Low prevalence of hepatitis E in Iceland: a seroepidemiological study. Scand J Gastroenterol. 2018;53(3): 293-296. DOI: 10.1080/00365521.2017.1420218.

Lucarelli C, Spada E, Taliani G, Chionne P, Madonna E, Marcantonio C, et al. High prevalence of anti-hepatitis E virus antibodies among blood donors in central Italy, February to March 2014. Euro Surveill. 2016;21(30): pii=30299. DOI: http://dx.doi.org/10.2807/1560-7917.ES.2016.21.30.30299.

Mansuy JM, Gallian P, Dimeglio C, Saune K, Arnaud C, Pelletier B, et al. A nationwide survey of hepatitis E viral infection in French blood donors. Hepatology. 2016;63(4): 1145-1154. doi: 10.1002/hep.28436.

Mansuy JM, Saune K, Rech H, Abravanel F, Mengelle C, Lhomme S, et al. Seroprevalence in blood donors reveals widespread, multi-source exposure to hepatitis E virus, southern France, October 2011. Euro Surveill. 2015;20(19): 27-34. PMID: 25990359.

[Mansuy](http://www.ncbi.nlm.nih.gov/pubmed/?term=Mansuy%20JM%5Bauth%5D) JM, [Bendall](http://www.ncbi.nlm.nih.gov/pubmed/?term=Bendall%20R%5Bauth%5D) R, [Legrand-Abravanel](http://www.ncbi.nlm.nih.gov/pubmed/?term=Legrand-Abravanel%20F%5Bauth%5D) F, [Sauné](http://www.ncbi.nlm.nih.gov/pubmed/?term=Saun%26%23x000e9%3B%20K%5Bauth%5D) K, [Miédouge](http://www.ncbi.nlm.nih.gov/pubmed/?term=Mi%26%23x000e9%3Bdouge%20M%5Bauth%5D) M, [Ellis](http://www.ncbi.nlm.nih.gov/pubmed/?term=Ellis%20V%5Bauth%5D) V, et al. Hepatitis E virus antibodies in blood donors, France. Emerg Infect Dis. 2011;17(12): 2309–2312. DOI: http://dx.doi.org/10.3201/eid1712.110371

Mansuy JM, Legrand-Abravanel F, Calot JP, Peron JM, Alric L, Agudo S, et al. High prevalence of anti-hepatitis E virus antibodies in blood donors from South West France. J Med Virol. 2008;80(2): 289–293. DOI: 10.1002/jmv.21056.

## Masia G, Orru G, Liciardi M, Desogus G, Coppola RC, Murru V, et al. Evidence of hepatitis E virus (HEV) infection in human and pigs in Sardinia, Italy. J Prev Med Hyg. 2009;50(4): 227-231.

## Mast EE, Kuramoto IK, Favorov MO, Schoening VR, Burkholder BT, Shapiro CN, et al. Prevalence of and risk factors for antibody to hepatitis E virus seroreactivity among blood donors in Northern California. J Infect Dis. 1997;176(1): 34-40.

## Mateos ML, Camarero C, Lasa E, Teruel JL, Mir N, Baquero F. Hepatitis E virus: relevance in blood donors and risk groups. Vox Sang. 1999;76(2): 78-80.

Meng XJ. Swine hepatitis E virus: cross-species infection and risk in xenotransplantation. Curr Top Microbiol Immunol. 2003;278: 185-216. PMID: 12934945.

Meng XJ, Wiseman B, Elvinger F, Guenette DK, Toth TE, Engle RE, et al. Prevalence of antibodies to hepatitis E virus in veterinarians working with swine and in normal blood donors in the United States and other countries. J Clin Microbiol. 2002;40(1): 117–122. DOI: 10.1128/JCM.40.1.117–122.2002.

## Mesquita JR, Valente-Gomes G, Conceicao-Neto N, Nascimento MSJ. Pet veterinarians have no increased risk of hepatitis E compared to the general population. J Med Virol. 2014;86(6): 954–956. Doi: https://doi.org/10.1002/jmv.23927.

Miletic Lovric M, Stojic Vidovic M, Hecimovic A, Mihaljevic I, Jemersic L, Strauss-Patko M, et al. Seroprevalence of hepatitis E among Croatian blood donors. Vox Sang. 2016;111(S1): 199.

## Mitsui T, Tsukamoto Y, Suzuki S, Yamazaki C, Masuko K, Tsuda F, et al. Serological and molecular studies on subclinical hepatitis E virus infection using periodic serum samples obtained from healthy individuals. J Med Virol. 2005;76(4): 526–533. DOI: 10.1002/jmv.20393.

Moaven L, Locarnini SA, van Asten M, Crofts N. Seroepidemiology of hepatitis E in selected Australian populations. J Med Virol*.* 1995;45(3): 326-330. https://doi.org/10.1002/jmv.1890450316.

Moher D, Liberati A, Tetzlaff J, Altman DG, The PRISMA Group. Preferred reporting items for systematic reviews and meta-analyses: The PRISMA Statement. PLoS Med. 2009;6: e1000097. doi:10.1371/journal.pmed.1000097.

Mor O, Bassal R, Michaeli M, Wax M, Ram D, Cohen-Ezra O, et al. Prevalence of hepatitis E virus antibodies, Israel, 2009–2010. Emerg Infect Dis. 2015;21(4): 692-694. DOI: http://dx.doi.org/10.3201/eid2104.140245.

Munn Z, Moola S, Lisy K, Riitano D, Tufanaru C. Methodological guidance for systematic reviews of observational epidemiological studies reporting prevalence and cumulative incidence data. Int J Evid Based Healthc. 2015;13(3): 147-153. doi: 10.1097/XEB.0000000000000054.

Munne MS, Altabert NR, Otegui MLO, Vladimirsky SN, Moreiro R, Espul MP, et al. Updating the knowledge of hepatitis E: new variants and higher prevalence of anti-HEV in Argentina. Ann Hepatol. 2014;13(5): 496-502.

Nasrallah G, Al Absi E, Ali N, Ghandour R, Taleb S, Hedaya L, et al. Is it time to start hepatitis E testing-donor centre perspective. Vox Sang. 2016;111(S1): 9-10.

Nemecek V, Dite P, Smejkalova P, Maly M, Kriz B. Serological survey of Hepatitis E in the Czech Republic. J Clin Virol*.* 2015;70(S1): S116-S117. https://doi.org/10.1016/j.jcv.2015.07.270.

Niederhauser C, Widmer N, Hotz M, Gowland P. Seroprevalance of hepatitis E virus (HEV) in the Swiss blood donors: Basis for future strategy for preventing HEV transmission to at risk individuals. Vox Sang. 2016;111(S1): 175.

Norder H, Karlsson M, Mellgren Å, Konar J, Sandberg E, Lasson A, et al. Diagnostic performance of five assays for anti-hepatitis E virus IgG and IgM in a large cohort study. J Clin Microbiol. 2016;54(3): 549–555. doi: 10.1128/JCM.02343-15.

Obriadina A, Meng JH, Ulanova T, Trinta K, Burkov A, Fields HA, et al. A new enzyme immunoassay for the detection of antibody to hepatitis E virus. J Gastroenterol Hepatol. 2002;17(S3): S360-S364.

Olsen B, Axelsson-Olsson D, Thelin A, Weiland O. Unexpected high prevalence of IgG-antibodies to hepatitis E virus in Swedish pig farmers and controls. Scand J Infect Dis. 2006;38(1): 55-58. DOI: 10.1080/00365540500321470.

Ooi WW, Gawoski JM, Yarbough PO, Pankey GA. Hepatitis E seroconversion in United States travelers abroad. Am J Trop Med Hyg. 1999;61(5): 822–824. DOI: https://doi.org/10.4269/ajtmh.1999.61.822.

O’Riordan J, Boland F, Williams P, Donnellan J, Hogema BM, Ijaz S, et al. Hepatitis E virus infection in the Irish blood donor population. Transfusion. 2016;56: 2868-2876. doi:10.1111/trf.13757.

Park HK, Jeong SH, Kim JW, Woo BH, Lee DH, Kim HY, et al. Seroprevalence of anti-hepatitis E virus (HEV) in a Korean population: comparison of two commercial anti-HEV assays. BMC Infect Dis. 2012;12: 142. DOI: 10.1186/1471-2334-12-142.

Paul DA, Knigge MF, Ritter A, Gutierrez R, Pilot-Matias T, Chau KH, et al. Determination of hepatitis E virus seroprevalence by using recombinant fusion proteins and synthetic peptides. J Infect Dis. 1994;169(4): 801-806.

Pavia M, Iiritano E, Veratti MA, Angelillo IF. Prevalence of hepatitis E antibodies in healthy persons in southern Italy. Infection. 1998;26(1): 32-35.

Pereira SS, Teixeira J, Abreu-Silva J, Oliveira RMS, Mesquita JR, Nascimento MSJ. A nationwide serosurvey of hepatitis E virus in the general population of Portugal. J Hepatol. 2016;64(2): S206. DOI: https://doi.org/10.1016/S0168-8278(16)01724-4.

Pischke S, Hiller J, Lutgehetmann M, Polywka S, Rybczynski M, Ayuk F, et al. Blood-borne hepatitis E virus transmission: a relevant risk for immunosuppressed patients. Clin Infect Dis. 2016;63(4): 569-570. doi: 10.1093/cid/ciw309.

Pischke S, Greer M, Hardtke S, Bremer B, Gisa A, Lehmann P, et al. Course and treatment of chronic hepatitis E virus infection in lung transplant recipients. Transpl Infect Dis. 2014;16: 333–339. DOI: 10.1111/tid.12183.

Pischke S, Heim A, Bremer B, Raupach R, Horn-Wichmann R, Ganzenmueller T, et al. Hepatitis E: an emerging infectious disease in Germany? Z Gastroenterol*.* 2011;49: 1255–1257. DOI: http://dx.doi.org/10.1055/s-0031-1273394

Pischke S, Malinski P, Suneetha PV, Lehner F, Heiringhoff KH, Barg-Hock H, et al. Should all patients with autoimmune hepatitis be screened for HEV-infection? J Hepatol. 2010;52: S135.

Pittaras T, Valsami S, Mavrouli M, Kapsimali V, Tsakris A, Politou M. Seroprevalence of hepatitis E virus in blood donors in Greece. Vox Sang. 2014;106: 387. DOI: 10.1111/vox.12122.

Psichogiou M, Tzala E, Boletis J, Zakopoulou N, Loutradi A, Maliori M, et al. Hepatitis E virus infection in individuals at high risk of transmission of non-A, non-B hepatitis and sexually transmitted diseases. Scand J Infect Dis. 1996;28: 443-445.

Puttini C, Riccio ML, Redi D, Tordini G, Cenerini M, Romanello F, et al. Seroprevalence of hepatitis E virus (HEV) infection in blood donors and renal transplant recipients: a retrospective study from central Italy. Infez Med. 2015;23(3): 253-256.

Rapicetta M, Monarca R, Kondili LA, Chionne P, Madonna E, Madeddu G, et al. Hepatitis E virus and hepatitis A virus exposures in an apparently healthy high-risk population in Italy. Infection. 2013;41(1): 69-76. DOI 10.1007/s15010-012-0385-8.

Reinheimer C, Allwinn R, Berger A. Hepatitis E: are psychiatric patients on special risk? Med Microbiol Immunol. 2012;201: 171–175. DOI 10.1007/s00430-011-0218-x.

Rey JA, Findor JA, Daruich JR, Velazco CC, Igartua EB, Schmee E, et al. Prevalence of IgG anti-HEV in Buenos Aires, a nonendemic area for hepatitis E. J Travel Med. 1997;4(2): 100-101.

Ricco G, Bonino F, Lanza M, Scatena F, Alfieri CM, Messa P, et al. New immunoassays for total, IgA and IgM antibodies against hepatitis E virus: Prevalence in Italian blood donors and patients with chronic liver or kidney diseases. Dig Liver Dis. 2016;48(5): 536-541. Doi: http://dx.doi.org/10.1016/j.dld.2016.01.007.

Riveiro-Barciela M, Buti M, Homs M, Campos-Varela I, Cantarell C, Crespo M, et al. Cirrhosis, liver transplantation and HIV infection are risk factors associated with hepatitis E virus infection. PLoS ONE 2014;9(7): e103028. doi:10.1371/journal.pone.0103028.

Sadik S, van Rijckevorsel GGC, van Rooijen MS, Sonder GJB, Bruisten SM. Seroprevalence of hepatitis E virus differs in Dutch and first generation migrant populations in Amsterdam, the Netherlands: a cross-sectional study. BMC Infect Dis. 2016;16: 659. DOI 10.1186/s12879-016-2007-z.

Sakata H, Matsubayashi K, Takeda H, Sato S, Kato T, Hino S, et al. A nationwide survey for hepatitis E virus prevalence in Japanese blood donors with elevated alanine aminotransferase. Transfusion. 2008;48(12): 2568-2576. doi: 10.1111/j.1537-2995.2008.01910.x.

Sargento C, Achando P, Silva E, Ferreira C, Tomaz J. Seroprevalence of antibodies and RNA for Hepatitis E virus in volunteer blood donors and patients with other viral hepatitis. Vox Sang. 2014;107(S1): 165.

Sargento C, Achando P, Ferreira C, Silva E, Tomaz J. Hepatitis E virus-should we reconsider its role in blood donor screening? Vox Sang. 2016;111(S1): 198.

Sauleda S, Ong E, Bes M, Janssen A, Cory R, Babizki M, et al. Seroprevalence of hepatitis E virus (HEV) and detection of HEV RNA with a transcription-mediated amplification assay in blood donors from Catalonia (Spain). Transfusion. 2015;55(5): 972–979. doi: 10.1111/trf.12929.

Schnegg A, Burgisser P, Andre C, Kenfak-Foguena A, Canellini G, Moradpour D, et al. An analysis of the benefit of using HEV genotype 3 antigens in detecting anti-HEV IgG in a European population. PLoS ONE. 2013;8(5): e62980. doi:10.1371/journal.pone.0062980.

Scotto G, Martinelli D, Centra M, Querques M, Vittorio F, Delli Carri P, et al. Epidemiological and clinical features of HEV infection: a survey in the district of Foggia (Apulia, Southern Italy). Epidemiol Infect. 2014;142(2): 287–294. doi:10.1017/S0950268813001167.

Shrestha AC, Seed CR, Flower RL, Rooks KM, Keller AJ, Harley RJ, et al. Hepatitis E virus and implications for blood supply safety, Australia. Emerg Infect Dis. 2014;20(11): 1940-1942. doi: 0.3201/eid2011.140412.

Slot E, Hogema BM, Riezebos-Brilman A, Kok TM, Molier M, Zaaijer HL. Silent hepatitis E virus infection in Dutch blood donors, 2011 to 2012. Euro Surveill. 2013;18(31): pii=20550.

Strakova P, Kriz B, Rudolf I, Hubalek Z. Seroprevalence study of hepatitis E virus infection in two districts of the Czech Republic. Epidemiol Mikrobiol Imunol. 2014;63: 92-94.

Stramer SL, Moritz ED, Foster GA, Ong E, Linnen JM, Hogema BM, et al. Hepatitis E virus: seroprevalence and frequency of viral RNA detection among US blood donors. Transfusion. 2016;56(2): 481–488. doi:10.1111/trf.13355.

Sulkowska E, Kubicka-Russel D, Liszewski G, Kopacz A, Letowska M, Grabarczyk P. Molecular and serological markers of hepatitis E virus infection (HEV) in Polish blood donors. Vox Sang. 2016;111(Suppl.1): 198-199.

## Sylvan SPE, Jacobson SH, Christenson B. Prevalence of antibodies to hepatitis E virus among hemodialysis patients in Sweden. J Med Virol. 1998;54(1): 38–43. https://doi.org/10.1002/(SICI)1096-9071(199801)54:1<38::AID-JMV6>3.0.CO;2-Q.

## Takahashi M, Tamura K, Hoshino Y, Nagashima S, Yazaki Y, Mizuo H, et al. A nationwide survey of hepatitis E virus infection in the general population of Japan. J Med Virol. 2010;82(2): 271–281. DOI: 10.1002/jmv.21678.

Takahashi M, Kusakai S, Mizuo H, Suzuki K, Fujimura K, Masuko K, et al. Simultaneous detection of immunoglobulin A (IgA) and IgM antibodies against hepatitis E virus (HEV) is highly specific for diagnosis of acute HEV infection. J Clin Microbiol. 2005;43(1): 49–56. doi:10.1128/JCM.43.1.49–56.2005.

Takeda H, Matsubayashi K, Sakata H, Sato S, Kato T, Hino S, et al. A nationwide survey for prevalence of hepatitis E virus antibody in qualified blood donors in Japan. Vox Sang. 2010;99(4): 307–313. DOI: 10.1111/j.1423-0410.2010.01362.x.

## Tarrago, D., Lopez-Velez, R., Turrientes, C., Baquero, F., Mateos M.L. 2000. Prevalence of hepatitis E antibodies in immigrants from developing countries. Eur. J. Clin. Microbiol. Infect. Dis.19, 309–311.

## Tei S, Kitajima N, Ohara S, Inoue Y, Miki M, Yamatani T, et al. Consumption of uncooked deer meat as a risk factor for hepatitis E virus infection: an age- and sex-matched case-control study. J Med Virol. 2004;74(1): 67–70. DOI 10.1002/jmv.20147.

## Teixeira J, Mesquita JR, Pereira SS, Oliveira RMS, Abreu-Silva J, Rodrigues A, et al. Prevalence of hepatitis E virus antibodies in workers occupationally exposed to swine in Portugal. Med Microbiol Immunol. 2017;206(1): 77–81. DOI 10.1007/s00430-016-0484-8.

Teshale EH, Denniston MM, Drobeniuc J, Kamili S, Teo CG, Holmberg SD. Decline in hepatitis E virus antibody prevalence in the United States from 1988-1994 to 2009-2010. J Infect Dis. 2015;211(3): 366-373. DOI: 10.1093/infdis/jiu466.

Toyoda K, Furusyo N, Takeoka H, Murata M, Sawayama Y, Hayashi J. Epidemiological study of hepatitis E virus infection in the general population of Okinawa, Kyushu, Japan. J Gastroenterol Hepatol. 2008a;23(12): 1885–1890. doi:10.1111/j.1440-1746.2008.05568.x.

van den Berg B, van der Eijk AA, Pas SD, Hunter JG, Madden RG, Tio-Gillen AP, et al. Guillain-Barré syndrome associated with preceding hepatitis E virus infection. Neurology. 2014;82(6): 491-497. doi: 10.1212/WNL.0000000000000111.

Verhoef L, Koopmans M, Duizer E, Bakker J, Reimerink J, van Pelt W. Seroprevalence of hepatitis E antibodies and risk profile of HEV seropositivity in The Netherlands, 2006–2007. Epidemiol Infect. 2012;140(10): 1838-1847. doi:10.1017/S0950268811002913.

Vilibic-Cavlek T, Vilibic M, Kolaric B, Jemersic L, Kucinar J, Barbic K, et al. Seroepidemiology of Hepatitis E in selected population groups in Croatia: a prospective pilot study. Zoonoses Public Health. 2016;63(6): 494–502. doi: 10.1111/zph.12254.

Vollmer T, Diekmann J, Johne R, Eberhardt M, Knabbe C, Dreier J. Novel approach for detection of hepatitis E virus infection in German blood donors. J Clin Microbiol. 2012;50(8): 2708–2713. doi:10.1128/JCM.01119-12.

Wenzel JJ, Preiss J, Schemmerer M, Huber B, Jilg W. Test performance characteristics of Anti-HEV IgG assays strongly influence hepatitis E seroprevalence estimates. J Infect Dis. 2013;207: 497–500. DOI: 10.1093/infdis/jis688.

Wenzel JJ, Sichler M, Schemmerer M, Behrens G, Leitzmann MF, Jilg W. Decline in hepatitis E virus antibody prevalence in southeastern Germany, 1996-2011. Hepatology. 2014;60(4): 1180-1186. doi: 10.1002/hep.27244

Wong KH, Liu YM, Ng PSP, Young BWY, Lee SS. Epidemiology of hepatitis A and hepatitis E infection and their determinants in adult Chinese community in Hong Kong. J Med Virol. 2004;72: 538-544. DOI: 10.1002/jmv.20040.

Xu C, Wang RY, Schechterly CA, Ge S, Shih JW, Xia NS, et al. An assessment of hepatitis E virus (HEV) in US blood donors and recipients: no detectable HEV RNA in 1939 donors tested and no evidence for HEV transmission to 362 prospectively followed recipients. Transfusion. 2013;53(10): 2505-2511. doi: 10.1111/trf.12326.

Yoon Y, Jeong HS, Yun H, Lee H, Hwang YS, Park B, et al. Hepatitis E virus (HEV) seroprevalence in the general population of the Republic of Korea in 2007-2009: a nationwide cross-sectional study. BMC Infect Dis. 2014;14: 517. doi: 10.1186/1471-2334-14-517.

Zaaijer HL, Yin MF,Lelie PN. Seroprevalence of hepatitis E in the Netherlands. Lancet. 1992;340(8820): 681. DOI: 10.1016/0140-6736(92)92224-4.

Zaaijer HL, Mauser-Bunschoten EP, ten Veen JH, Kapprell HP, Kok M, van den Berg HM, et al. Hepatitis E virus antibodies among patients with hemophilia, blood donors, and hepatitis patients. J Med Virol. 1995;46(3): 244-246.

Zanetti AR, Dawson GJ; the Study Group of Hepatitis E. Hepatitis type E in Italy: a seroepidemiological survey. J Med Virol. 1994;42: 318-320.

Zervou EZ, Politis CP, Hassapopoulou EH, Vini MV, Parara MP, Kavallierou LK, et al. Prevalence of hepatitis E virus (HEV) infection in blood donors and multi-transfused patients in Greece. Vox sang. 2015;109(Suppl. 1): 242-243.
